# Crystal structure of the collagen prolyl 4-hydroxylase (C-P4H) catalytic domain complexed with PDI: Toward a model of the C-P4H α_2_β_2_ tetramer

**DOI:** 10.1016/j.jbc.2022.102614

**Published:** 2022-10-18

**Authors:** Abhinandan V. Murthy, Ramita Sulu, Andrey Lebedev, Antti M. Salo, Kati Korhonen, Rajaram Venkatesan, Hongmin Tu, Ulrich Bergmann, Janne Jänis, Mikko Laitaoja, Lloyd W. Ruddock, Johanna Myllyharju, M. Kristian Koski, Rik K. Wierenga

**Affiliations:** 1 Faculty of Biochemistry and Molecular Medicine, University of Oulu, Oulu, Finland; 2 Biocenter Oulu, University of Oulu, Oulu, Finland; 3 Scientific Computing Department, STFC Rutherford Appleton Lab., RCaH, Harwell Campus, Didcot, United Kingdom; 4 Department of Chemistry, University of Eastern Finland, Joensuu, Finland

**Keywords:** collagen, crystallography, structure–function, thioredoxin, 2-oxoglutarate-dependent dioxygenase, intersubunit disulfide bridge, BCO, Biocenter Oulu, CAT, catalytic domain of the α-subunit of C-P4H, C-P4H, collagen prolyl 4-hydroxylase, Cr-P4H, *Chlamydomonas reinhardtii* P4H isoform 1, DSBH, double-stranded β-helix, HIF-P4H-2, hypoxia-inducible factor P4H, type-2, LSSR, local structural similarity restraint, MALS, multi-angle light scattering, MS, mass spectrometry, MTP, microsomal triglyceride transfer protein, NEM, *N*-ethylmaleimide, PDB, Protein Data Bank, PDI, protein disulfide isomerase, P4H-TM, transmembrane P4H, PSB, peptide-substrate-binding domain, SEC, size-exclusion chromatography

## Abstract

Collagen prolyl 4-hydroxylases (C-P4H) are α_2_β_2_ tetramers, which catalyze the prolyl 4-hydroxylation of procollagen, allowing for the formation of the stable triple-helical collagen structure in the endoplasmic reticulum. The C-P4H α-subunit provides the N-terminal dimerization domain, the middle peptide-substrate-binding (PSB) domain, and the C-terminal catalytic (CAT) domain, whereas the β-subunit is identical to the enzyme protein disulfide isomerase (PDI). The structure of the N-terminal part of the α-subunit (N-terminal region and PSB domain) is known, but the structures of the PSB-CAT linker region and the CAT domain as well as its mode of assembly with the β/PDI subunit, are unknown. Here, we report the crystal structure of the CAT domain of human C-P4H-II complexed with the intact β/PDI subunit, at 3.8 Å resolution. The CAT domain interacts with the **a**, **b’**, and **a’** domains of the β/PDI subunit, such that the CAT active site is facing bulk solvent. The structure also shows that the C-P4H-II CAT domain has a unique N-terminal extension, consisting of α-helices and a β-strand, which is the edge strand of its major antiparallel β-sheet. This extra region of the CAT domain interacts tightly with the β/PDI subunit, showing that the CAT-PDI interface includes an intersubunit disulfide bridge with the **a’** domain and tight hydrophobic interactions with the **b’** domain. Using this new information, the structure of the mature C-P4H-II α_2_β_2_ tetramer is predicted. The model suggests that the CAT active-site properties are modulated by α-helices of the N-terminal dimerization domains of both subunits of the α_2_-dimer.

Collagen prolyl 4-hydroxylases (C-P4Hs; Enzyme Commission no.: 1.14.11.2), localized in the lumen of the endoplasmic reticulum, catalyze a vital cotranslational and post-translational modification of procollagen polypeptides required for the assembly of the collagen triple helix (1, 2, 3, 4). C-P4Hs are Fe(II) and 2-oxoglutarate-dependent dioxygenases, which use molecular oxygen to hydroxylate proline residues that are present at the Y position of the -X-Y-Gly- repeats of the procollagen chain. The Fe(II) ion is bound in the active site to a fully conserved His-X-Asp--His sequence motif, being important for activating the O_2_ molecule. The catalysis involves oxidative decarboxylation of 2-oxoglutarate to succinate and carbon dioxide and the hydroxylation of procollagen peptidyl prolines into 4-hydroxyprolines. This modification is needed to provide stability for the triple helical collagen molecules. Subsequently, the collagens are secreted into the extracellular matrix where they are further modified and assembled into various supramolecular structures, and where they are involved in, for example, cell adhesion, cell migration, and cell remodeling during growth, differentiation, and wound healing (2, 4, 5, 6). Collagens play also important roles in many pathological states, like fibrosis and cancer (2, 7, 8, 9). C-P4Hs are α_2_β_2_ heterotetramers (1, 2, 3). The α-subunit contains the catalytic site for the C-P4H activity (10), and the β-subunit is identical to protein disulfide isomerase (PDI, Enzyme Commission no.: 5.3.4.1), which is an enzyme and chaperone that functions in protein folding in the endoplasmic reticulum (11, 12, 13). In mammals, there are three isoforms of the α-subunit (Fig. 1) (14, 15), which are complexed with the same β-subunit, giving rise to three C-P4H tetramers, referred to as C-P4H-I, C-P4H-II, and C-P4H-III according to their α-subunit. Two splice variants have been characterized for both α(I) and α(II) (16, 17) (Fig. S1). These splice variants have small sequence differences in the catalytic domain. C-P4H-I is the major isoenzyme, and its inactivation in mouse leads to early death during embryonic development, whereas mice lacking C-P4H-II are almost normal, but in combination with reduced amounts of C-P4H-I, show specific phenotypic abnormalities (18, 19). C-P4H-I and C-P4H-II have different affinities for procollagen-like substrate peptides (20, 21). C-P4H-III is far less characterized than the first two isoforms (15), and its *in vivo* role is as yet unknown.

**Figure 1:**
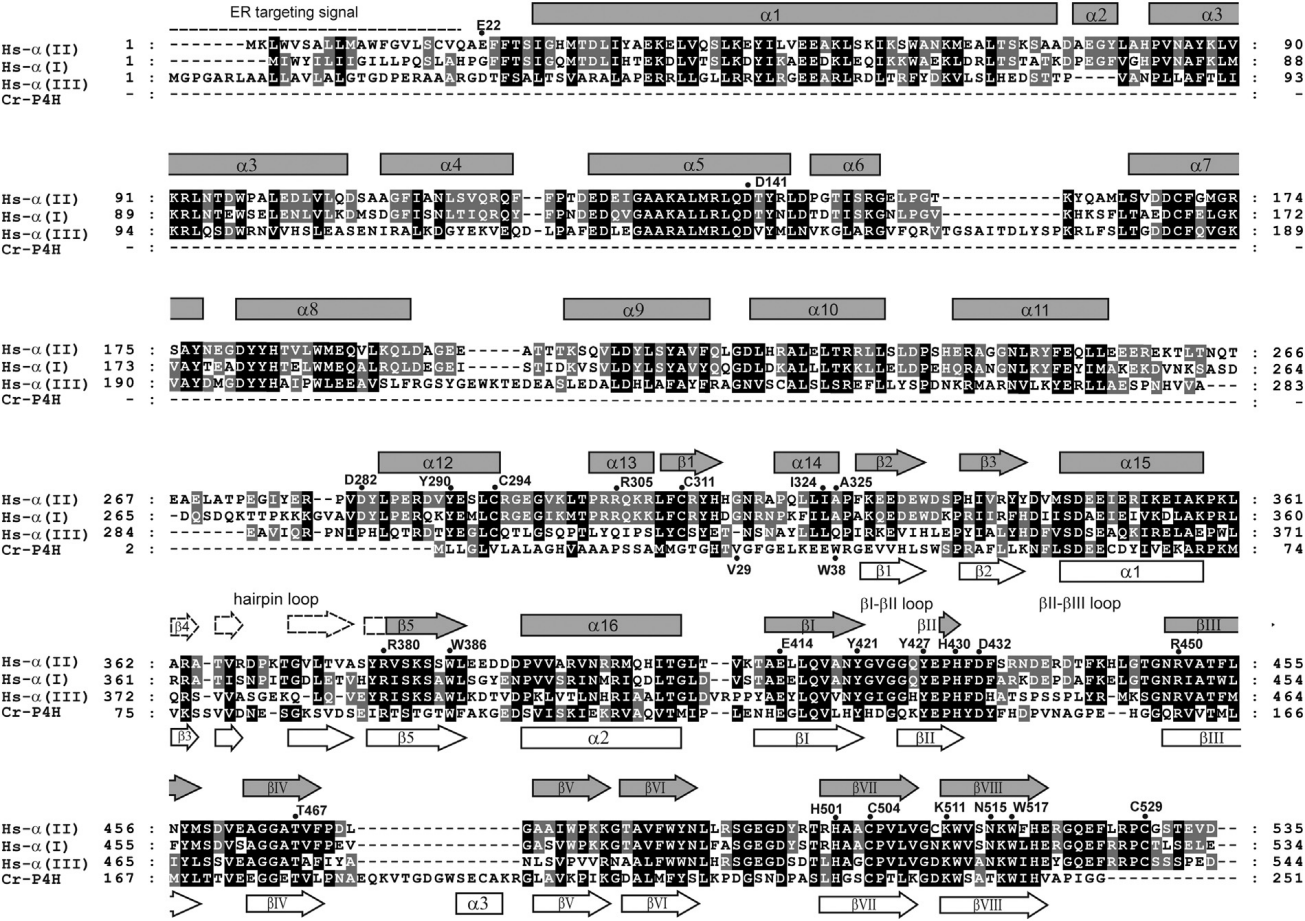
**Sequence alignment of the α-subunits of C-P4H-II, C-P4H-I, and C-P4H-III (labeled as Hs-α(II), Hs-α(I), and Hs-α(III)) as well as of Cr-P4H.** The C-P4H sequences include the endoplasmic reticulum–targeting sequences (this region is shown with a *dashed line* above the sequences). Cr-P4H concerns the algal *Chlamydomonas reinhardtii* isoform-1 prolyl 4-hydroxylase. Helices and β-strands are indicated by *cylinders* and *arrows*. The annotations above and below the sequences concern, respectively, Hs-α(II) and Cr-P4H. The nomenclature of the secondary structure elements of Cr-P4H is the same as used previously (26). V29 marks the beginning of the Cr-P4H construct, and W38 identifies the first ordered residue of the Cr-P4H structure (Protein Data Bank entry: 3GZE). The secondary structure annotation of Hs-α(II) refers to the structure of the double-domain construct (22) and of the C-P4H-II–Δ281 complex (this study). The *dotted secondary structure elements* of the Hs-α(II) hairpin loop (which is disordered in the structure of the C-P4H-II–Δ281 complex) is assigned as observed in the Cr-P4H structure. The residues highlighted above the Hs-α(II) sequence refer to important residues mentioned in the text. D141, D282, R305, and A325 identify the N-terminal residue of the C-P4H-II-Δ140, C-P4H-II-Δ281, C-P4H-II-Δ304, and C-P4H-II-Δ324 constructs, respectively, which have been investigated in these studies.

The α-subunits of human C-P4H-I and C-P4H-II share a sequence identity of about 65%, whereas α(I) and α(II) share about 35% sequence identity with α(III). The C-P4H α-subunit of each of the isoforms consists of four parts: the N-terminal (dimerization) domain, the peptide-substrate-binding (PSB) domain (middle domain), a linker region (L, of unknown structure and function), and the C-terminal catalytic domain (Fig. 2) (21, 22, 23). Crystal structures of the PSB domains of human C-P4H-I and C-P4H-II (24, 25), with and without bound proline-rich peptides, show the mode of binding of these peptides in a groove lined by highly conserved tyrosine residues. Furthermore, a crystal structure of a construct consisting of the N-terminal (dimerization) domain and the PSB domain, which is referred as the double-domain construct (Fig. 2), shows how the N-terminal domain forms a protein–protein dimer interface *via* a coiled-coil helical dimerization motif, whereas the PSB domains point away from this interface (22).

**Figure 2:**
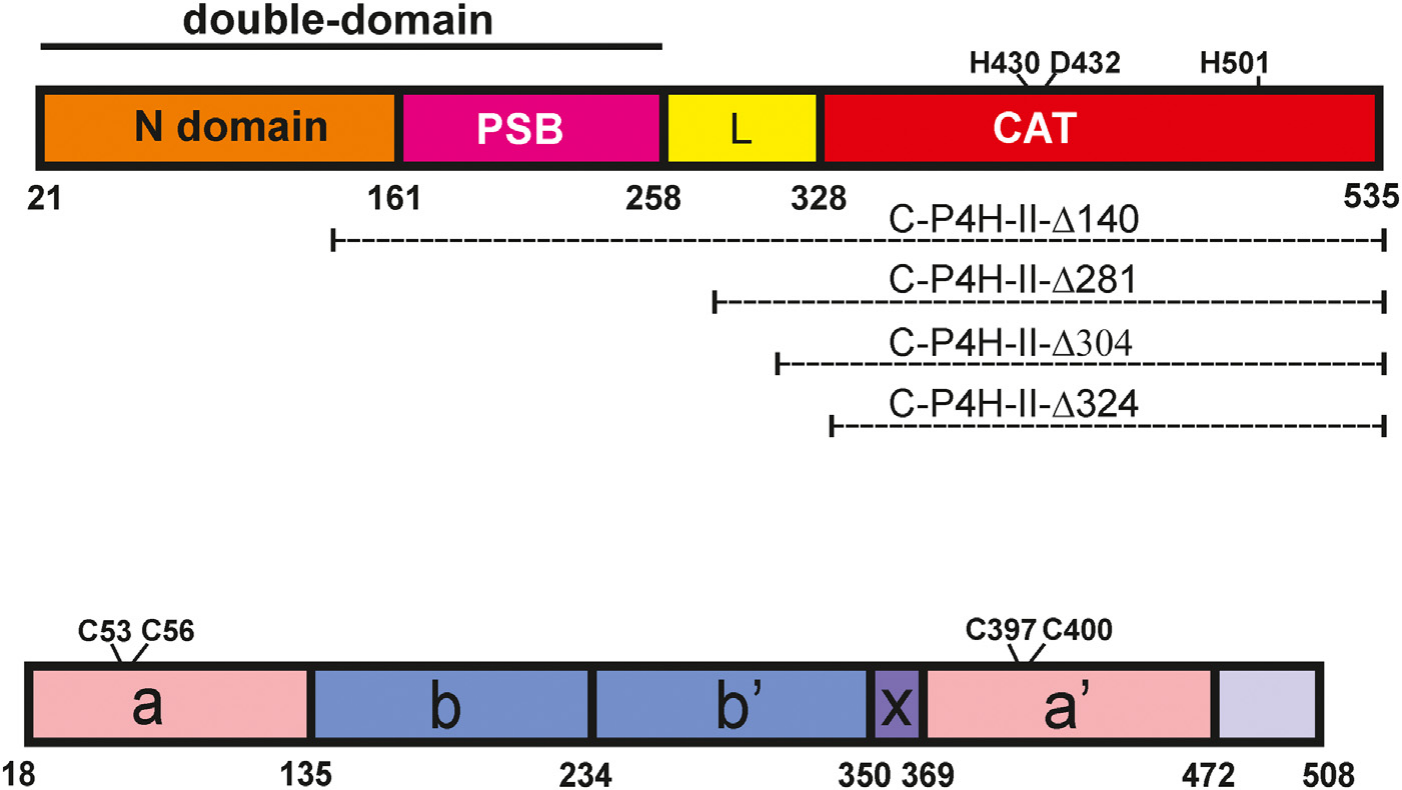
**Schematic diagrams of the domain organization of the α and β/PDI subunits of C-P4H.***Top*, domain structure of the α-subunit. The construct that includes the N-terminal dimerization domain and the middle peptide-substrate-binding (PSB) domain is referred to as the double-domain construct of which the structure is known (22). The truncation variants of the α-subunit, being studied here, are identified below the schematic figure. The structure and function of the linker region, residues 258 to 328, labeled L, are unknown from previous studies, but the studies reported here show that its C-terminal region, residues 284 to 328, forms an integral part of the catalytic (CAT) domain. The catalytic residues of the CAT domain are identified above the schematic figure. These residues are fully conserved in the P4H family of enzymes (Fig. S1). *Bottom*, domain structure of the β/PDI subunit. The **a**, **b**, **b’**, and **a’** domains adopt the thioredoxin fold. The **a** and **a’** domains are also referred to as the catalytic domains, and their catalytic cysteines are identified. The proximal cysteines are Cys53 and Cys397. C-P4H, collagen prolyl 4-hydroxylase; PDI, protein disulfide isomerase.

The structure of the human C-P4H CAT domain is as yet unknown, but crystal structures of a monomeric *Chlamydomonas reinhardtii* P4H isoform 1 (Cr-P4H) (about 35% sequence identity with the human C-P4H-I CAT domain (Fig. 1)), both in its unliganded form (without peptide) and complexed with a peptide substrate, (Ser-Pro)_5_, have been solved (26, 27). Like in all 2-oxoglutarate-dependent dioxygenases, the core of the fold consists of eight antiparallel β-strands (labeled as βI to βVIII), having the spiral topology of the double-stranded β-helix fold (the DSBH-fold) (28, 29) and forming a major and a minor β-sheet. In the 2-oxoglutarate-dependent dioxygenases, the cofactor 2-oxoglutarate is bound in a deeply buried cavity between these two sheets, shielded from bulk solvent by the bound substrate molecule (28, 29). In the structure of Cr-P4H complexed with its proline-rich substrate peptide, the peptide is bound in a tunnel shaped by two substrate-binding loops, being the hairpin loop and the βII-βIII loop (27). In the unliganded Cr-P4H structure, the substrate-binding loops are disordered and/or adopt different conformations (26). In addition, in the unliganded structure, the βI-βII catalytic loop (residues Tyr134 to Tyr140) adopts a different conformation when comparing liganded and unliganded structures. The conformational flexibility properties of the two substrate-binding loops and the catalytic loop are important for the catalytic function of this enzyme (27).

PDI has a four-domain structure, referred to as the **a**, **b**, **b’**, and **a’** domains (Fig. 2) (11). These domains have a thioredoxin fold. The **a** domain and **a’** domain of PDI are also referred to as its catalytic domains, as they both have the conserved CGHC-sequence motif (Figs. 2 and S2). The cysteines of this motif are the CAT residues, needed to catalyze the formation, breaking, and isomerization of disulfide bridges of the PDI substrate proteins in their folding pathway. Studies have shown that the β/PDI subunit is required for providing the soluble and catalytically competent conformation of the α-subunit, but it is not understood how it is assembled with the α-subunit, how it is involved in intersubunit disulfide bridges, and what its role is in the procollagen hydroxylation reaction mechanism of the mature C-P4H α_2_β_2_ tetramer (30, 31, 32, 33, 34). The crystal structure of human PDI is known in oxidized (having a disulfide bond between the two cysteines of the CGHC-motif) and reduced (the cysteines of the CGHC motif are reduced) forms (35). Also known is the structure of the human heterodimer microsomal triglyceride transfer protein (MTP), which includes PDI as its β-subunit (36). The α-subunit of MTP is a lipid-binding protein, and its amino acid sequence and fold are not related to the α-subunit of C-P4H. In addition, the crystal structure of ERp57 (a PDI family member, present in the endoplasmic reticulum), complexed with tapasin (not related to the α-subunits of C-P4H and MTP), is known (37).

Small-angle X-ray scattering studies of mature C-P4H-I suggest that in the mature α_2_β_2_ C-P4H-I tetramer, the CAT domains of the two α-subunits point away from the α_2_-dimer interface (formed by the two N-terminal dimerization domains) and are capped by the β/PDI subunit, positioned at both ends of the elongated tetramer, suggesting that the β/PDI subunit interacts solely with the CAT domain (23). These small-angle X-ray scattering studies have provided also the shape information of proteolytically truncated forms of C-P4H-I, which were copurified together with the mature recombinant C-P4H-I. One of these truncated forms was a heterodimer complex of a truncated α-chain (consisting of the PSB and CAT domains but lacking the N-terminal dimerization domain) complexed with the intact β/PDI subunit. The truncation site of this heterodimer is near residue Asp139 of the α-subunit of C-P4H-I (just before the PSB domain) (Fig. 1) (23). The more detailed characterization of such a complex can provide key information on the structure of the CAT domain and its assembly with the β/PDI subunit, which is currently completely lacking. However, the optimal α-subunit truncation site for obtaining a heterodimer that has only the CAT domain of the α-subunit, complexed with the intact β/PDI subunit, is not clear, as it depends on the unknown structural and functional role of the linker region (Fig. 2).

Here, we report on the characterization of two truncated human C-P4H-II variants in which the α-subunit is lacking either the N-terminal dimerization domain (C-P4H-II-Δ140) or the dimerization domain and the PSB domain (C-P4H-II-Δ281). These truncated variants of C-P4H-II are stable and soluble heterodimer complexes. The C-P4H-II-Δ281 complex could be crystallized, and its crystal structure reveals the structure of the CAT domain as well as its interactions with the β/PDI subunit. This crystal structure, together with the AlphaFold2 (DeepMind company and EMBL-EBI) (38) model of the complete α-subunit dimer, enabled the prediction of the mode of assembly of the C-P4H-II α_2_β_2_ heterotetramer, which is also discussed.

## Results and discussion

### Expression, purification, and properties of mature C-P4H-II and two truncated C-P4H-II variants

Considerable proteolytic degradation has been a problem in previous experiments on recombinant production of human C-P4H-I in the *Escherichia coli* Origami strain (23). Therefore, the *E. coli* CyDisCo expression system (39, 40), which has been used for the expression of MTP for crystallization (36), was tested for production of human C-P4H-I and C-P4H-II. For this purpose, codon-optimized constructs of the α-subunit of C-P4H-I and C-P4H-II (Table S1) were coexpressed with β/PDI, being part of the CyDisCo expression vector. The complexes were expressed and purified as described in the Experimental procedures section. The C-P4H-I and C-P4H-II samples were pure after the size-exclusion chromatography (SEC) step. The yield of C-P4H-II was better, and proteolytic degradation was less than for C-P4H-I. Mass spectrometric (MS) peptide mapping of C-P4H-II confirmed that the purified C-P4H-II includes the N-terminal and C-terminal peptides of both subunits (Table S2). Therefore, subsequent experiments, aimed at finding the best possible truncation sites of the α-subunit that would allow for the recombinant generation and purification of a complex of the CAT domain assembled with β/PDI, were done with C-P4H-II. Four truncated constructs of the α-subunit were tested, referred to as C-P4H-II-Δ140, C-P4H-II-Δ281, C-P4H-II-Δ304, and C-P4H-II-Δ324, in which the first 140, 281, 304, and 324 residues, respectively, were deleted (Fig. 2). The truncated α-subunits were expressed with a 6× His tag at the N terminus, whereas the coexpressed β/PDI subunit was without a tag, identical as used for the expression of the mature C-P4H-I and C-P4H-II complexes (Table S1). Coexpression of PDI with the C-P4H-II-Δ140 construct (encoding for PSB and CAT, Fig. 2) as well as with the C-P4H-II-Δ281 construct (encoding for CAT only, Fig. 2) resulted in soluble complexes, whereas the C-P4H-II-Δ304 and C-P4H-II-Δ324 constructs were only expressed as insoluble proteins, which could not be purified. Apparently, the residues between 281 and 304 include amino acids that are required to form a soluble complex of the CAT domain, assembled with β/PDI. The C-P4H-II-Δ140 and C-P4H-II-Δ281 complexes were purified using the same protocols as used for mature C-P4H-I and C-P4H-II. The SDS-PAGE gel analysis showed that these samples are highly pure, and truncated α and intact β-subunits are present, which was also confirmed by MS characterization. Characterization by multiangle light scattering (MALS) (Fig. S3 and Table 1) confirmed that the purified C-P4H-II-Δ140 and C-P4H-II-Δ281 complexes are heterodimers. The CD spectra (Fig. S4*A*) show that wildtype C-P4H-II and its two truncated variants have the expected secondary structure properties. The CD melting curves of the truncated complexes (Fig. S4*B*) show *T*_m_ values of 50.2 and 53.2 °C for, respectively, C-P4H-II-Δ140 and C-P4H-II-Δ281, slightly higher than the *T*_m_ of mature C-P4H-II (*T*_m_ = 46.5 °C) (Table 1), which confirms that the truncated complexes indeed are stable proteins. Activity assays show that the two truncated variants have C-P4H activity, although to a lesser extent than mature C-P4H-II (Table 1). Subsequently, crystallization experiments were carried out with these purified complexes, which resulted in diffraction quality crystals of the C-P4H-Δ281 complex, as outlined in the next section.

**Table 1:** The molecular weights, the *T*_m_ values, and the activity assay results of mature C-P4H-II and its truncated variants

Construct	Molecular weight (SEC–MALS^a^)	*T*_m_ (CD^b^)	Indirect assay^c^ with 2-oxo[1-^14^C]glutarate and (PPG)_10_ substrate, measuring the hydroxylation coupled amount of formed^14^CO_2_	Direct assay^d^ with [^14^C]proline-labeled procollagen substrate, measuring the amount of formed 4-hydroxy[^14^C]proline	
	(kDa)	(C)	DPM/(nmole active site)	DPM/(nmole active site)	
				Experiment 1	Experiment 2
C-P4H-II	230	46.5 ± 0.2	320,500 ± 20,900	83,700	114,500
C-P4H-II–Δ140	101	50.2 ± 0.2	44,300 ± 3100	7600	5800
C-P4H-II–Δ281	85	53.2 ± 0.2	25,700 ± 1900	1000	800

a The SEC–MALS graphs are shown in Fig. S3.

b The CD melting curves are shown in Fig. S4*B*.

c The mean values of the results of three experiments are provided.

d The results of two experiments are reported. The listed activity values obtained by the indirect and direct assays cannot be compared directly.

### The determination of the crystal structure of the C-P4H-II-Δ281 variant

**Figure 3:**
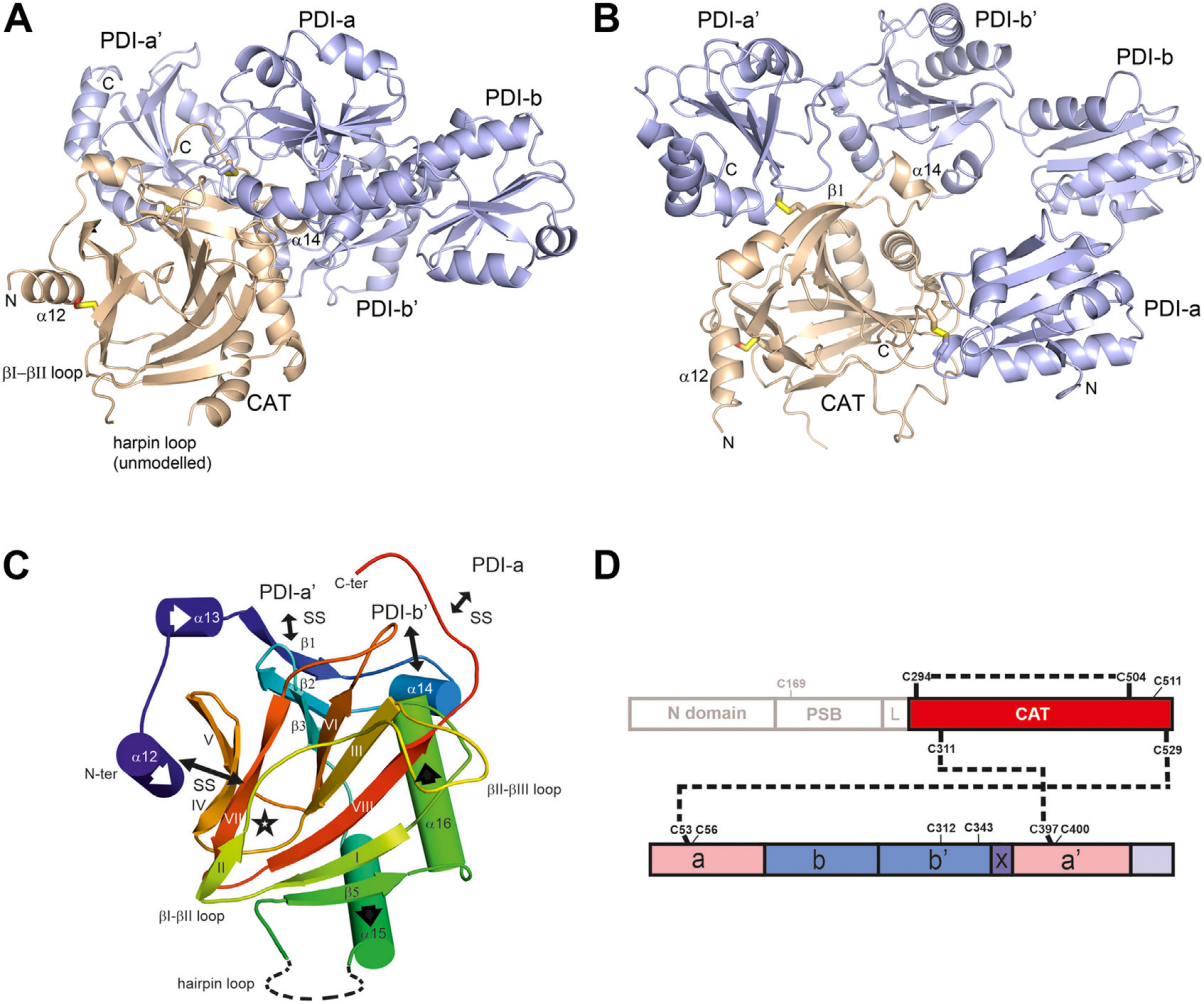
**The structureof the C-P4H-II-Δ281 complex.***A*, standard view, showing that the CAT active site (*brown*, near the βI-βII loop) faces the bulk solvent. In this view, the β/PDI subunit (*blue*) is located at the back side of the CAT domain. *B*, *top view*, rotated by 90^°^ around the horizontal with respect to panel (*A*), visualizing the mode of assembly of the CAT domain with the domains of the β/PDI subunit. The sulfur atoms of the intersubunit and intrasubunit disulfide bridges are shown in *yellow* color. *C*, ribbon diagram of the fold of the CAT domain of the C-P4H-II-Δ281 complex (*standard view*). The DSBH-fold is formed by β-strands βI to βVIII; the minor sheet is shown on the *left* side, and the major sheet is shown on the *right* side. The *star* identifies the catalytic site. The *ribbon diagram* is color coded by color ramping from N terminus (*dark blue*) to C terminus (*red*). The substrate-binding loops (hairpin loop and βII-βIII loop) and the catalytic loop (βI-βII loop) are labeled. The secondary structure elements α12, α13, β1, and α14 form a unique N-terminal extension of the C-P4H CAT domain, not present in previously reported structures of P4Hs (Fig. S1). SS identifies a disulfide bridge. *D*, schematic visualization of the C-P4H-II-Δ281 construct, visualizing also the observed disulfide bridges in the crystal structure of this complex, also referred to as the CAT-PDI complex. CAT, catalytic domain of the α-subunit of C-P4H; C-P4H, collagen prolyl 4-hydroxylase; DSBH, double-stranded β-helix; PDI, protein disulfide isomerase.

The C-P4H-II-Δ281 complex was crystallized in space group R3. A complete dataset was collected at the P14 beamline (of PETRA III, operated by the EMBL outstation at DESY, Hamburg) at 3.8 Å resolution (Table 2). The structure was solved by molecular replacement using as search models the crystal structures of the monomeric algal Cr-P4H and of the **a**, **b’**, and **a’** domains of human PDI, as described in the Experimental procedures section. The asymmetric unit consists of two C-P4H-II-Δ281 complexes, which are formed by the CAT domain of the α subunit, complexed with the complete β/PDI subunit (Fig. 3). This complex is also referred to as the CAT-PDI complex. At the end of the refinement protocol, the main-chain and side-chain conformations of both copies of the CAT-PDI complex are well defined by the electron density map (Figs. S5–S7), and there are no significant structural differences between these two CAT-PDI complexes. The CAT domains are better ordered than the β/PDI subunits. At the N terminus of both α-subunits, the model starts at residue Leu284 and the β/PDI subunits start at Glu21. Not included in the model of the CAT domain are also the C-terminal residues of the α-subunit (residues 534–535) and residues of the hairpin loop (known from the Cr-P4H studies to be important for substrate binding, Fig. 1). The highly acidic C-terminal region of PDI (residues 477–498) is also completely disordered and not seen in the electron density maps. The latter finding agrees with the notion that the PDI C-terminal KDEL sequence functions as the endoplasmic reticulum retention signal (31) and that the C-terminal region of PDI is not needed for the tetramer assembly (32).

**Table 2:** Data collection and statistics of the data processing and refinement of the structure of the C-P4H-II–Δ281 complex

Data collection	
Beamline	P14, PETRA III (DESY, EMBL Hamburg)
Wavelength (Å)	0.9763
Detector	DECTRIS EIGER X 16M
Temperature (K)	100
Data processing software	XDS/Aimless

**Data processing statistics**	

Unit cell parameters (Å,°)	a = 252.9
	b = 252.9
	c = 89.4
	α, β = 90
	γ = 120
Space group	R3
Resolution range (Å)^a^	46.69–3.85 (3.99–3.85)
*V*_m_ (Å^3^/Da)	3.2
Molecules per asymmetric unit	Two heterodimer complexes
Number of observations	107,514 (10,863)
Multiplicity	5.3 (5.5)
Completeness (%)	99.9 (100)
I/σ(I)	8.2 (0.7)
*R*_merge_ (%)	6.2 (167.3)
CC(1/2)	0.996 (0.495)
*R*_pim_ (%)	4.7 (123.5)

**Refinement statistics**	

Resolution (Å)	46.69–3.85
*R*_work_ (%)	24.4
*R*_free_ (%)	27.8
Number of unique reflections	20,135
**Number of atoms**^b^	
All protein atoms	11,088
α-subunit 1 (chain A)	1920
α-subunit 2 (chain B)	1897
β-subunit 1 (chain C)	3631
β-subunit 2 (chain D)	3640
Sulfate (SO_4_)	10
Rmsd bond length (Å)	0.011
Rmsd bond angle (°)	1.6
**Average*****B*****-factors (Å^2^)**	
All atoms	73.2
α-subunit 1 (chain A)	49.1
α-subunit 2 (chain B)	41.7
β-subunit 1 (chain C)	89.3
β-subunit 2 (chain D)	86.4
The two sulfate solutes	24.5
**Ramachandran plot**^c^	
Favored (%)	96.5
Allowed (%)	2.7
Outliers (%)	0.8
**PDB entry**	7ZSC

a The numbers in parentheses refer to the outer shell.

b Nonhydrogen atoms.

c Calculated by MolProbity.

### The overall structure of the CAT domain and its mode of assembly with the β/PDI subunit

The CAT domain is folded into the typical DSBH-fold (Fig. 3), as also seen in other P4H structures, such as Cr-P4H (being the closest homolog) (26, 27), transmembrane P4H (P4H-TM) (41), hypoxia-inducible factor P4H, type-2 (HIF-P4H-2) (42), hypoxia-inducible factor P4H, type-1 (43), viral P4H (44), and bacterial P4H (45). The N-terminal region of the α-subunit of the C-P4H-II-Δ281 complex, residues 284–328, which was previously proposed to be part of the linker region between the PSB and CAT domains (Fig. 2) (23), is actually an integral part of the CAT domain (Fig. 3). This unique N-terminal extension of the CAT domain, not seen in any other P4H family member (Fig. S1), forms two short α-helices (α12 and α13) followed by an extra β-strand (β1, including Cys311) of the major sheet of the DSBH-fold and another short α-helix (α14, including Ile324), before continuing into the secondary structure elements known from the other P4H structures (Fig. S1). Also the C terminus is more extended in the C-P4H-II-Δ281 structure, as compared with Cr-P4H, by eight residues, and this extension includes Cys529 (Fig. 1). Cys529, as well as Cys311 of the N-terminal extension, is conserved in the C-P4H enzymes (Fig. S1).

The structure of the C-P4H-II-Δ281 complex shows that in the CAT domain there is an intrasubunit disulfide bridge between two conserved cysteines, Cys294 (of the N-terminal extension of the CAT domain, in the α12-helix) and Cys504, located in β-strand βVII of the DSBH-fold (Fig. 3). The latter β-strand provides a side chain (His501) that sequesters the active site Fe(II) ion. MS peptide mapping of C-P4H-II showed that treatment with DTT reduces these two cysteines to free thiols. However, corresponding disulfide-linked peptides were not detected in the native sample despite extensive searching of the MS data, possibly because of low ionization efficiency. These peptides were also not detected in the *N*-ethylmaleimide (NEM)-labeled sample, in agreement with the presence of a disulfide bridge between Cys294 and Cys504 (Tables S2 and S3). An intrasubunit disulfide bridge in the α-subunit has been suggested by previous experimental studies with C-P4H-I, where the importance of the conserved Cys294 and Cys504 (Fig. S1) for the assembly and function of C-P4H-I has been noted (46, 47, 48). This intrasubunit disulfide bridge in CAT is unique for this subfamily of P4Hs because Cys294 is located in the unique N-terminal extension of CAT. However, in some other P4H enzymes, Cys504 is conserved (Fig. S1) and in fact also engaged in an intrasubunit disulfide bridge, for example, in P4H-TM (41) and Cr-P4H (26) but not in HIF-P4Hs (42, 43).

The assembly of the CAT-PDI heterodimer complex is stabilized by interactions of the unique N-terminal and C-terminal extensions of the CAT domain with the **a’**, **b’**, and **a** domains of PDI, whereas there are no interactions between the CAT domain and the **b** domain (Fig. 3). Two regions of the CAT N-terminal extension are involved in these interactions, located around Cys311 (of the β1 β-strand) with the **a’** domain and around Ile324 (α14-helix) with the **b’** domain. A third main interaction region is near Cys529 (near the C terminus of the CAT domain), which interacts with the **a** domain of the β/PDI subunit (Fig. 3). These CAT-PDI interactions are at the “back” side of the CAT domain, in such a way that the active site of the CAT domain points to bulk solvent, and the presence of the β/PDI subunit does not affect the conformational flexibility of its three active-site loops.

### The interactions of the CAT domain with the β/PDI subunit

**Figure 4:**
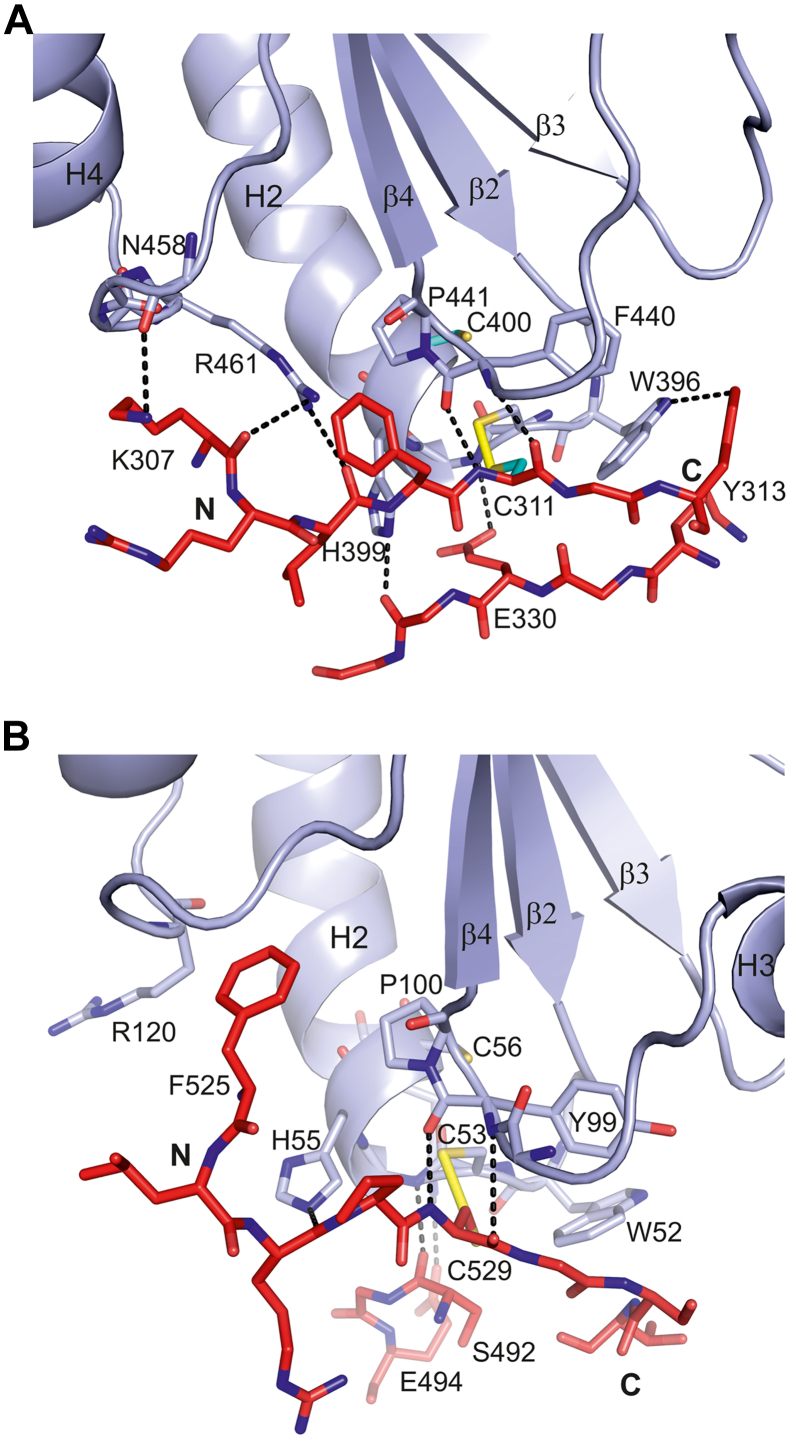
**The interactions of the CAT domain with the a’ and a domains of the β/PDI subunit.***A*, Cys311 of the β1 strand of the CAT domain forms an intersubunit disulfide bridge with the proximal Cys397 of the **a’** domain. *B*, Cys529 of the extended C-terminal tail of the CAT domain forms a disulfide bridge with the proximal Cys53 of the **a** domain. The **a’** and **a** domains are shown in l*ight blue ribbon* and *sticks*. The CAT peptide is shown in *red color*, and the sulfur atoms are shown in *yellow color*. The sequences of the peptides near Cys311 (KRLFCRYHH) and Cys529 (FLRPCGSTE) are very different; N and C identify the N-terminal and C-terminal regions of these peptides. The *dotted lines* highlight hydrogen-bond interactions, including the hydrogen-bond interactions of OE1(Glu330) and O(Ser492) of the α-subunit with the main chain peptide NH of the glycine of the CGHC motif in **a’** (*A*) and **a** (*B*), respectively. The orientation of the **a’** and **a** domains of panels (*A*) and (*B*) is similar as the orientation of the thioredoxin fold depicted in the schematic diagram of Fig. S8. Stereo images of these two interaction sites, including the corresponding electron density maps, are provided in Figs. S6 and S7. CAT, catalytic domain of the α-subunit of C-P4H; PDI, protein disulfide isomerase.

The CAT-PDI protein–protein interfaces near Cys311 and Cys529 (of the CAT domain) include disulfide bridges with Cys397 and Cys53 (of PDI), respectively, which are the proximal cysteines (Fig. 2) of the CGHC motifs of the **a’** and **a** domains (Figs. 4, S6, and S7). The CGHC motif is located at the N-terminal end of helix H2 of the thioredoxin fold (Fig. S8). The presence of these intersubunit disulfide bridges was validated by calculating omit maps and maps obtained from models of structures in which the disulfide bridges have been switched from an intersubunit disulfide bridge to an intrasubunit disulfide bridge between the proximal and distal cysteines of the CGHC motifs (Fig. S9). This switch requires only minimal structural changes. The obtained electron density maps suggest that the Cys311 disulfide bridge has high occupancy, whereas the Cys529 disulfide bridge could be partially intrasubunit, between Cys53 and Cys56 of the PDI **a** domain CGHC motif, and partially intersubunit, with the latter geometry occurring predominantly (Fig. S9).

The sequences around the Cys311 (β1 β-strand of the CAT domain) and Cys529 (C terminus of CAT domain) that interact with the PDI domains are very different (Figs. 4 and S10), being KRLFCRYHH and FLRPCGSTE, respectively. However, residues Cys311 and Cys529 have the same main chain hydrogen-bond interactions with Phe440 (of the **a’** domain) and Tyr99 (of the **a** domain), respectively, which are possible because of the conserved *cis*-(Phe440-Pro441) and *cis*-(Tyr99-Pro100) peptide bond (Figs. S6 and S7). The interactions of Cys311 and Cys529 with the **a’** and **a** domains are complemented with interactions of the β2-region (near Glu330) and the βVI–βVII loop region (near Ser492) of the CAT domain with the CGHC loop of the **a’** and **a** domains of PDI, respectively (Fig. S10). Cys311 and Cys529 are conserved cysteines in the C-P4H enzymes (Fig. S1), and their presence at the CAT-PDI interfaces is in agreement with earlier work on C-P4H-I (46, 48) that highlighted the importance of these two cysteines for the formation of the functional tetrameric C-P4H enzyme. In the two failed constructs of this study, C-P4H-II-Δ304 and C-P4H-II-Δ324, the truncation site is just before or just after Cys311, and therefore, the key structural elements present in this region of the structure are disrupted, resulting in insoluble proteins that could not be purified.

It is possible that the intersubunit disulfide bridges formed between CAT and PDI are transient disulfide bonds, which are easily converted into intrasubunit disulfide bridges (between the cysteines of the CGHC-cysteines) by nucleophilic attack of the distal cysteine of PDI, as suggested by other studies (37, 49). This breaks the intersubunit disulfide bridge and generates an oxidized β/PDI subunit on dissociation of the complex, as observed experimentally for C-P4H-I (47). This is also in agreement with the MS peptide mapping data of the mature C-P4H-II complex (Tables S2 and Table S3), where it is found that the sample treatment dissociates the complex, generating oxidized β/PDI active sites, having a disulfide bridge between the catalytic cysteines.

**Figure 5:**
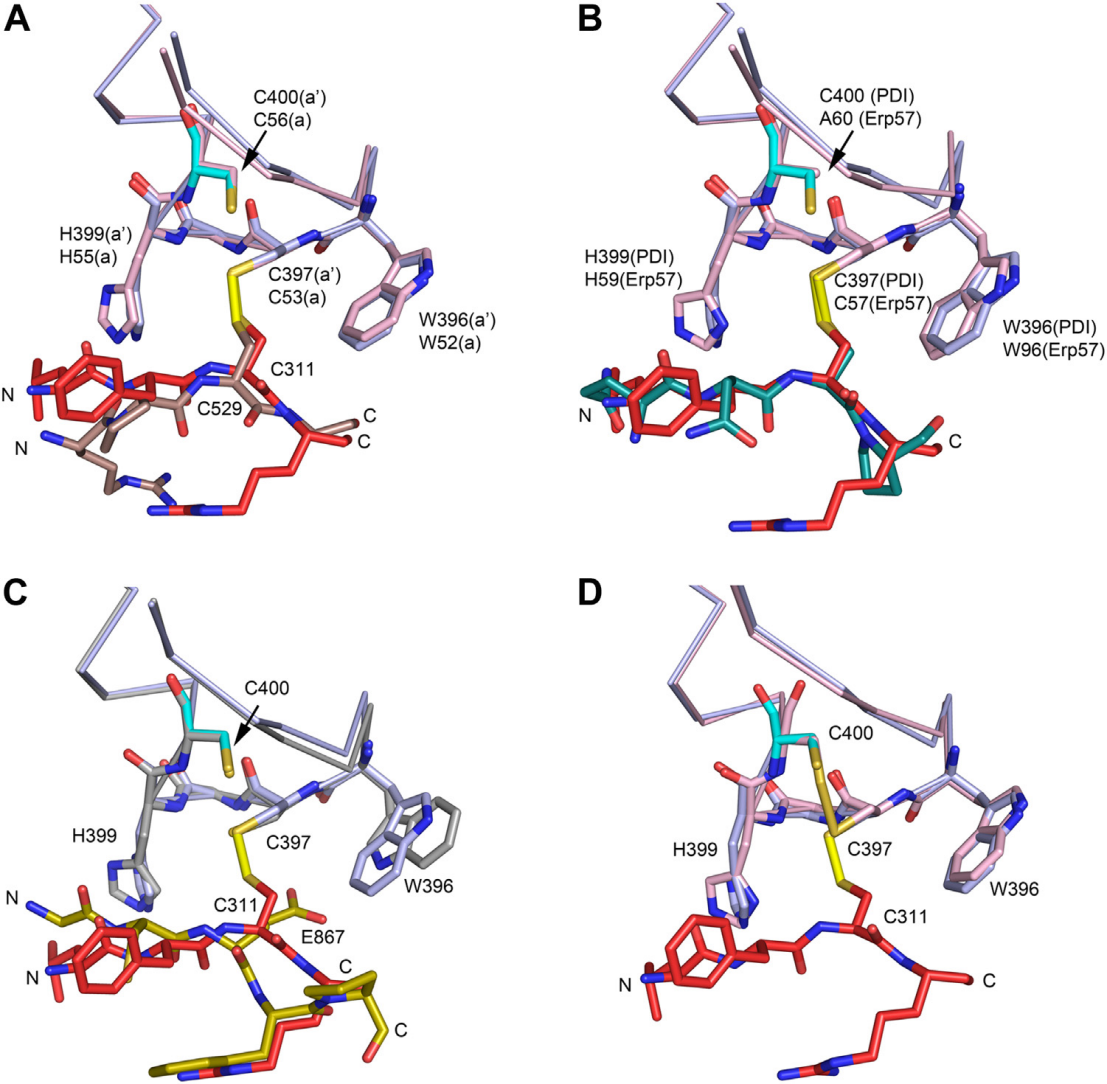
**Comparison of the geometry of the CGHC interaction sites of human PDI (in various oxidation states and complexes) and human ERp57.** Similar view as for Figures 4 and S8. The **a’** interaction site with the CAT domain of the C-P4H-II–Δ281 complex is used as a reference structure, and the Cα-atoms of β-strand β2 and helix H2 of the PDI domains have been used for the superpositioning. The **a’** domain is in *light blue color*, the CAT region is in *red color*, and the sulfur atoms are shown in *yellow color*. *A*, comparison of the **a’** and **a** (*light red*) interaction sites of the C-P4H-II–Δ281 structure. *B*, comparison of the **a’** and **a** (*cyan*) interaction sites of the complexes of C-P4H-II–Δ281 and ERp57–tapasin (Protein Data Bank [PDB] entry: 3F8U), respectively. In the latter complex, the distal cysteine of the **a** domain of ERp57 is mutated into an alanine. *C*, comparison of the **a’** interaction sites of the complexes of C-P4H-II–Δ281 and MTP (PDB entry: 6I7S) (*yellow*). In the MTP complex, the CGHC cysteines of PDI are reduced. *D*, comparison of the geometry of the **a’** interaction site of the C-P4H-II–Δ281 complex with the geometry of the unliganded and oxidized structure (with disulfide bridge) (*magenta*) of **a’** of human PDI (PDB entry: 4EL1). CAT, catalytic domain; MTP, microsomal triglyceride transfer protein; PDI, protein disulfide isomerase.

The geometries of the intersubunit disulfide bridges formed between Cys311(CAT) and Cys397(PDI) and between Cys529(CAT) and Cys53(PDI) are very similar, as shown when superimposing the **a** and **a’** domains (Fig. 5*A*), and this geometry is within acceptable ranges of disulfide bridges when compared with the geometry of disulfide bridges in other proteins (50). This geometry is also preserved in the structure of the ERp57–tapasin complex when considering the interface of the **a** domain of ERp57 and tapasin (Fig. 5*B*). The PDI geometry is also conserved in the structure of the MTP complex when considering the interface of the **a’** domain of PDI and the MTP α-subunit (Fig. 5*C*). In the ERp57–tapasin complex (the assembly is visualized in Fig. S11), the distal cysteine is mutated into an alanine, facilitating the trapping of a complex in which there is a stable covalent disulfide bond between the proximal cysteine of the ERp57 CGHC motif and the cysteine of tapasin (37). In the MTP complex, the β/PDI cysteines are both reduced, and a covalent complex is not observed in this structure. In Figure 5*D*, the comparison of the structures of the **a’** domain of the C-P4H-II-Δ281 complex (with the intersubunit disulfide bond) with the structure of the oxidized **a’** domain of the uncomplexed PDI (with a disulfide bond between Cys397 and Cys400) shows no structural differences between these two states. Clearly, the various oxidation states of the PDI domains can be accommodated with minor structural changes.

**Figure 6:**
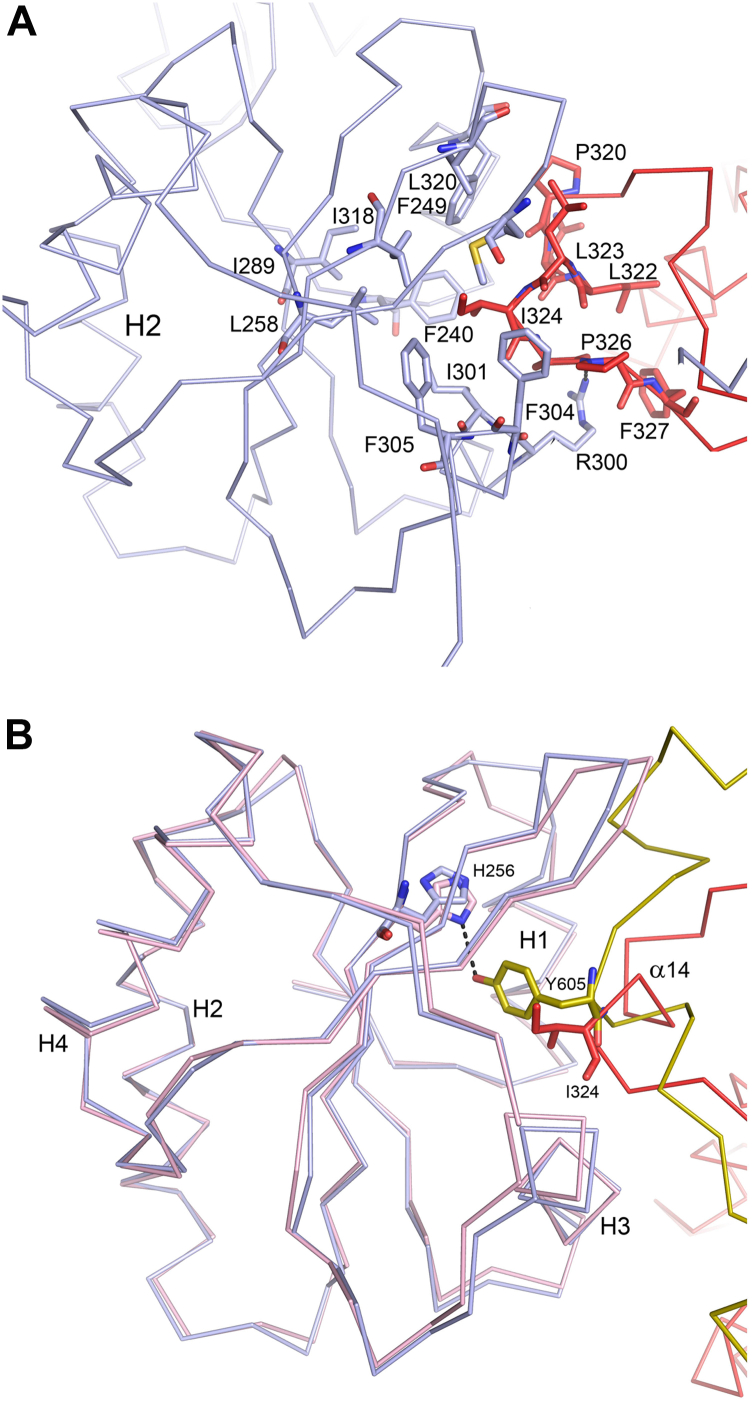
**The interactions of the CAT domain with the hydrophobic pocket of the b’ domain of the β/PDI subunit.** The viewing direction is similar as for Figures 4 and S8. *A*, the hydrophobic and conserved α14 region (including Ile324) of the CAT domain (*red ribbon* and *sticks*) interacts with the hydrophobic peptide-binding pocket of **b’** of the β/PDI subunit (*light blue ribbon* and *sticks*). The key side chains of the residues shaping the hydrophobic peptide-binding pocket of the **b’** domain are also shown. The stereo view is given in Fig. S12. *B*, the **b’** domains of the superimposed structures of the C-P4H-II-Δ281 complex (*light blue*) and the MTP complex (Protein Data Bank entry: 6I7S, shown as *ribbons* and *sticks*, colored *light magenta*). Small adjustments of the side chains of the **b’** hydrophobic pocket allow the side chains of Ile324 (C-P4H-II-Δ281 complex, *red*) and Tyr605 (MTP complex, *yellow*) to bind in the same hydrophobic peptide-binding pocket, formed between helices H1 and H3. In the MTP complex, the hydroxyl group of Tyr605 is hydrogen bonded to the side chain of His256 (marked with a *dotted line*). CAT, catalytic domain of the α-subunit of C-P4H; MTP, microsomal triglyceride transfer protein; PDI, protein disulfide isomerase.

The third important interface between the CAT domain and the β/PDI subunit concerns the hydrophobic interactions of the Ile324 side chain of the CAT domain with the hydrophobic pocket of the PDI **b’** domain. Various studies on the interactions of PDI with peptides have highlighted the importance of the **b’** hydrophobic pocket for the PDI peptide interactions (51, 52). This interaction site is formed by a region of the thioredoxin fold, which is different from the **a** and **a’** domain interaction sites, being shaped by residues of helix H1 and helix H3 (Figs. 6*A* and S12). The rim of this pocket is formed by residues Phe240, Phe249 (of helix H1), Ile301, Phe304, Phe305 (of helix H3), and Ile318. The bottom of this hydrophobic pocket is formed by the side chains of residues Leu258, Ile289, and Ile291, which protrude out of the core β-strands. The importance of Ile289 for the peptide-binding properties of PDI has been reported (34). Ile324 of the LLIA sequence of the α14-helix of the CAT domain points into this hydrophobic pocket. The hydrophobicity of the LLIA region is highly conserved in the C-P4H family (Fig. S1). In the MTP crystal structure, the mode of assembly of the α-subunit with the β/PDI subunit is very different (Fig. S11); however, the same hydrophobic peptide-binding pocket of the **b’** domain is also in MTP interacting with the MTP α-subunit (36). In this complex, Tyr605 of the α-subunit points into this hydrophobic pocket with its aromatic ring stacked between Leu258 and Phe249, and the hydroxyl moiety of Tyr605 has a hydrogen bond with the side chain of His256 (Fig. 6*B*). There is no further sequence or structural similarity in the regions of the α-subunits of the C-P4H-II-Δ281 and MTP complexes, which interact with the **b’** domain, but nevertheless, there are no conformational differences between the structural elements of the **b’** domain that form the binding pocket in these two complexes. This is consistent with the required promiscuity of substrate binding that this pocket must have as part of PDIs role in oxidative folding. The structure of the C-P4H-II-Δ281 complex suggests that the **a’** and **b’** interfaces contribute much to the stability of the complex, which is consistent with previous mutational studies, which have shown that the **a’** and **b’** domains are most important for the assembly of the mature complex (33, 34).

### The structure of the active site of the CAT domain

**Figure 7:**
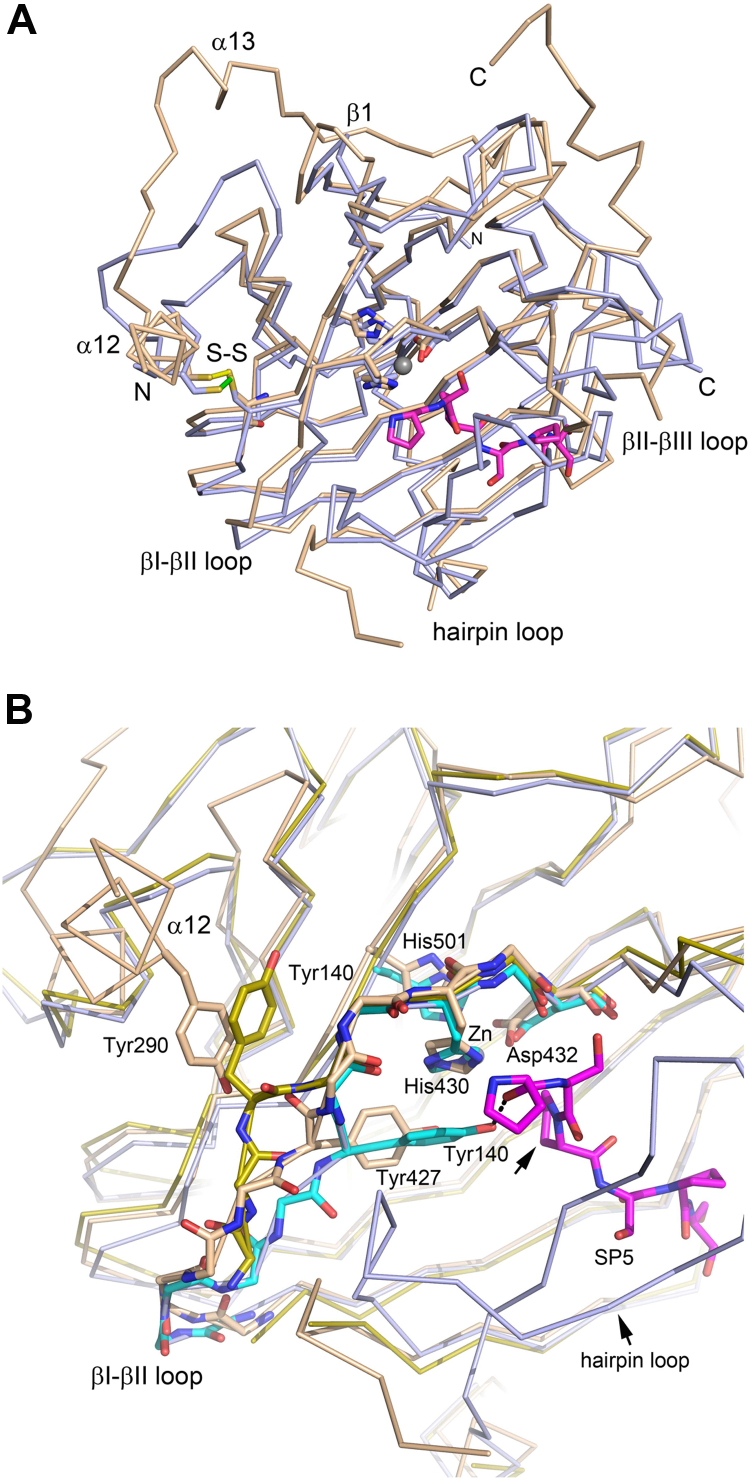
**Comparison of the structures of the CAT domains of C-P4H-II-Δ281 and Cr-P4H.***A*, superimposed Cα traces of the CAT domains of the C-P4H-II-Δ281 complex (*light brown*) and Cr-P4H (*light blue*, Protein Data Bank [PDB] entry: 3GZE, molecule A) in complex with Zn(II) (*gray sphere*) (the Zn(II) ion has replaced the Fe(II) active site ion, resulting in an incompetent active site) and the (Ser-Pro)_5_ peptide substrate (*magenta sticks*). The α12-helix, the α13-helix, and β-strand β1 are additional structural elements of C-P4H-II-Δ281, missing in Cr-P4H. Also the C terminus (labeled with “C”) is more extended in C-P4H-II-Δ281. The loop regions, which are important for peptide substrate binding (the hairpin and βII–βIII loop), are labeled. The hairpin loop is disordered in the structure of the C-P4H-II-Δ281 complex, and it is in the “closed” conformation in the Cr-P4H peptide complex. The side chains of the three important catalytic amino acids, two histidines and one aspartate, coordinating the bound Zn(II) ion in the 3GZE structure, are shown. Also the intrasubunit disulfide bond (labeled as S-S) of the conserved cysteine of the βVII-strand (Cys504 in the C-P4H-II-Δ281 complex and Cys230 in Cr-P4H) with Cys294 (α12-helix in the N-terminal region of the CAT domain of C-P4H-II-Δ281) and Cys195 (extended βIV–βV loop of Cr-P4H), respectively, is shown. *B*, zoomed in view of panel (*A*) of the active site of the CAT domain, highlighting the βI-βII loop region (Gly422-Pro429) and the following His-X-Asp----His motif (residues His430, Asp432, and His501), which are shown as *sticks*, like the side chain of Tyr427. Included is also the superimposed βI-βII loop region as seen (i) in the structure of the Cr-P4H enzyme–substrate complex (PDB entry: 3GZE, molecule A, as in panel (*A*), *light blue*) and (ii) as seen in the structure of Cr-P4H without bound peptide (molecule B, PDB entry: 2JIG, *yellow*), highlighting the two different conformations of Tyr140. In the structure of the Cr-P4H enzyme substrate complex, the Tyr140 side chain is hydrogen bonded to the bound (Ser-Pro)_5_ peptide substrate, as shown with a *dashed line*. The proline residue to be hydroxylated by the catalytic site is identified with an *arrow*. Zn identifies the Zn(II) ion–binding site in the catalytic site of the Cr-P4H enzyme–substrate complex. In the structure of Cr-P4H without bound peptide, the Tyr140 side chain is rotated outward (*yellow*). The latter conformation is not possible in the C-P4H-II-Δ281 structure, as its corresponding tyrosine (Tyr427) would clash with Tyr290 of the α12-helix. Stereo views are given in Fig. S13. CAT, catalytic domain of the α-subunit of C-P4H; C-P4H, collagen prolyl 4-hydroxylase; Cr-P4H, *Chlamydomonas reinhardtii* P4H isoform 1.

The CAT active site is in its apo form, without a bound Fe(II) ion, which is consistent with the presence of EDTA in the protein buffer of the crystallization experiment. The main chain and side chains of the fully conserved His-X-Asp----His motif (Fig. S1) (residues His430, Asp432, and His501 in C-P4H-II) known to bind the Fe(II) ion (10) superimpose well on the structure of the corresponding region of the Cr-P4H complex (Figs. 7 and S13) known to bind the active site of Fe(II) ion (26). Also, the main-chain and side-chain atoms of the conserved residues Tyr421, Thr467, and Lys511, which are proposed to interact with the buried 2-oxoglutarate carboxylate group (as seen in the Cr-P4H complex structure with a 2-oxoglutarate analog (26)), superimpose well on the corresponding residues of the Cr-P4H complex.

From the crystal structures of Cr-P4H, it is known that the two active-site substrate-binding loops, which are the hairpin loop (residues Ser77 to Thr94 in Cr-P4H) and the βII-βIII loop (residues His148 to Gly159 in Cr-P4H) sequester its (Ser-Pro)_5_ substrate peptide, by which the peptide binds in a tunnel, so that residue Pro5 of this peptide points toward the catalytic metal ion (27). It can be noted that also in HIF-P4H-2 (Protein Data Bank [PDB] entry: 3HQR) (42), the hairpin loop is important for peptide binding. In the structure of the C-P4H-II-Δ281 complex, the active site is unliganded and the hairpin loop (residues Arg363 to Val376) is disordered. The βII-βIII region (residues Arg435 to Gly448) is included in the model of the CAT-PDI complex, but it has been built in weak density. Importantly, these two loops are not near the interaction site of the β/PDI subunit and therefore can adopt other conformations, for example, in the presence of bound substrate peptide. The sequences of the hairpin loop and the βII-βIII loop are not well conserved when comparing the sequences of human C-P4H-I, C-P4H-II, and C-P4H-III (Fig. 1). Large sequence divergence is also apparent when taking into account the sequences of other P4H enzymes (Fig. S1). For example, in P4H-TM, the hairpin loop is much more extensive, forming even a separate domain, known as the Ca(II)-binding EF domain (Fig. S1) (41). In this respect, it is also interesting to note that the splice variants of C-P4H-I affect the sequence of the hairpin loop (Fig. S1), whereas those of C-P4H-II change the sequence and length of the βII-βIII loop (Fig. S1).

Previous structural studies of Cr-P4H have shown that conformational flexibility of the βI-βII catalytic loop (residues Tyr134 to Tyr140 in Cr-P4H, Fig. 7*B*) (26, 27) is important for the function. In the structure of Cr-P4H complexed with its substrate (Ser-Pro)_5_, the side chain of the conserved Tyr140 of the βI-βII region is pointing inward and contacts Pro5 of the (Ser-Pro)_5_ peptide that is hydroxylated in the catalytic cycle. This tyrosine, which is Tyr427 in C-P4H-II, is highly conserved in P4H sequences (Fig. S1), and indeed, in the structure of the HIF-P4H-2 peptide complex, the corresponding tyrosine (Tyr310) also interacts with the bound peptide. Structure analysis of the complex of Cr-P4H with bound peptide suggests that the product of the hydroxylation reaction, which is the hydroxylated proline, will clash with the side chain of Tyr140 (27). This then could result in the tyrosine side chain flipping out, being linked to an induced conformational switch of the βI-βII region. Such an out conformation is captured in the unliganded structure, as shown in Figure 7*B*. In the C-P4H-II-Δ281 structure, the βI-βII region near Tyr427 adopts a conformation, which is different from both the competent in-conformation as well as the unliganded out-conformation of Cr-P4H (Fig. 7*B*). The notion that the conformation of the βI-βII region of the C-P4H-II-Δ281 complex is different from the conformation competent for catalysis is consistent with the observation that the C-P4H-II-Δ281 complex has low catalytic activity (Table 1). The flipped-out conformation of Tyr427 would clash with Tyr290 of the α12-helix (as shown in Fig. 7*B*), showing that the flipped-out conformation, as observed in the Cr-P4H structure, is not possible in the structure of the assembly that is captured in this crystal form of the C-P4H-II-Δ281 complex.

### The predicted structure of the mature C-P4H-II α_2_β_2_ tetramer

**Figure 8:**
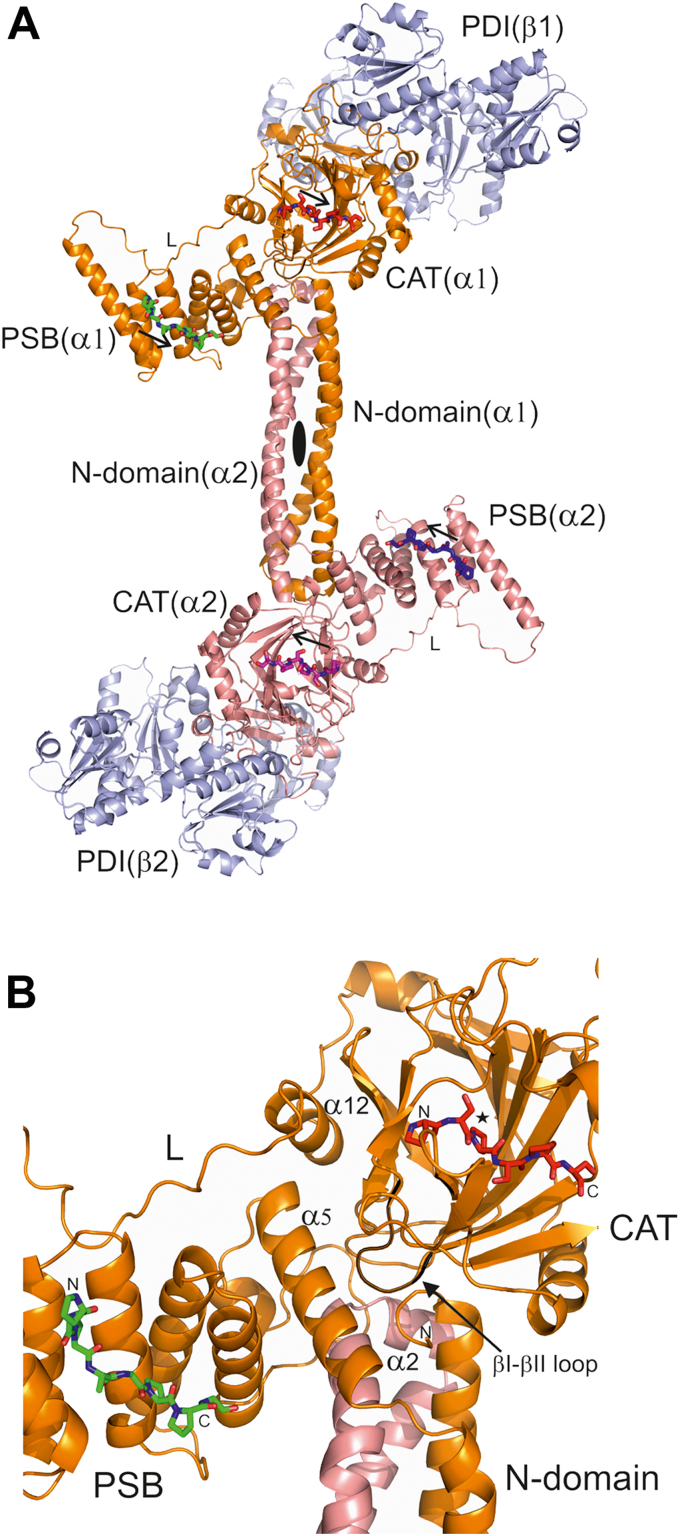
**The predicted structure of the mature C-P4H-II α**_**2**_**β**_**2**_**tetramer.***A*, the mature tetramer. The α-subunits are in *orange* and *salmon colors*, respectively, and the β/PDI subunits (at the outer edge of the α_2_-dimer) are colored *light blue*. The view is down the twofold axis (identified by a *black oval*) of the α_2_-dimer. The locations of the peptide-binding sites of the PSB and CAT domains are visualized with bound peptides, as predicted by using the structures of the Cr-P4H complexed with its substrate (Ser-Pro)_5_ (Protein Data Bank [PDB] entry: 3GZE), superimposed on the CAT domains (*red* and *magenta* peptides) and the PSB-II-peptide complex (PDB entry: 6EVN), superimposed on the PSB domains (*green* and *blue* peptides). The *upper* and *lower* CAT active sites, identified by the *red* and *magenta* colored peptides, respectively, are related by the dimer twofold axis. The *arrows* show the N-to-C direction of the bound peptides. “L” identifies the linker region between the PSB domain and the CAT domain. *B*, zoomed-in view of the PSB and CAT domains of the mature C-P4H-II tetramer, shown in the *upper part* of *A*. The CAT catalytic site is marked by a *star*. The βI-βII loop (*black ribbon*) is close to the α5-helix and the N terminus (labeled “N”) of the same α-subunit as well as to the α2-helix of the other α-subunit of the α2-dimer (colored in *salmon*). CAT, catalytic domain of the α-subunit of C-P4H; C-P4H, collagen prolyl 4-hydroxylase; Cr-P4H, *Chlamydomonas reinhardtii* P4H isoform 1; PDI, protein disulfide isomerase; PSB, peptide-substrate–binding domain.

The structure prediction AlphaFold2 protocol (38), as implemented in a CoLab notebook (AlphaFold2_advanced.ipynb), was used to predict the structure of the α_2_-dimer, using the sequence of the mature α-subunit, starting at Glu22 (Fig. 1). Subsequently, the CAT domain of the structure of the C-P4H-II-Δ281 complex was superimposed on the two CAT domains of the predicted structure of the α_2_-dimer, providing then the predicted structure of the mature C-P4H α_2_β_2_ complex (Fig. 8). The fold of the α-subunit as predicted for the α_2_-dimer is essentially the same as seen in the model obtained for the monomeric α-subunit using either Robetta (53) or AlphaFold2 (38), using the protocols as described in the Experimental procedures section. The confidence score (the pLDDT score) of the AlphaFold2 model of the α_2_-dimer is high for most regions of the model (Fig. S14). The lowest scores are for the sequence region just after the PSB domain (and before the α12-helix of the C-P4H-II-Δ281 complex) and for the sequence regions of the two substrate-binding loops (the hairpin loop and the βII-βIII loop) of the CAT domain. The latter two loops are disordered (the hairpin loop) or built in weak density (the βII-βIII loop) in the C-P4H-II-Δ281 structure. The structure of the N-terminal double-domain region of the model agrees very well with its crystal structure (22). In the model, the CAT domain folds over the double-domain region in such a way that the minor sheet of the DSBH-fold is closely interacting with the α5-helix of the same α-subunit. The βI-βII region of the CAT domain is interacting with (i) the α5-helix and (ii) the N terminus of the same α-subunit as well as with (iii) the α2-helix of the other α-subunit of the α_2_-dimer (Fig. 8). The residues of the α5-helix are highly conserved in sequence alignments (Figs.1 and S1), as was noted already when discussing the structural properties of the double-domain dimer crystal structure (22). The sequence alignment (Figs. 1 and S1) shows that Phe24 (at the N terminus of the α-subunit) and Pro83 (near the C terminus of the α2-helix) are also conserved. These conserved residues are in the model close to the βI-βII region. The predicted structure of the βI-βII region is the same as in the structure of the Cr-P4H peptide complex (27). The sequence alignment (Fig. S1) shows that this region is highly conserved in the C-P4H sequences (Gly-(V/I/M)-Gly-Gly) but being different from the sequences of other P4Hs.

In the model of the C-P4H α_2_β_2_ complex, the PSB domain is a protrusion of the core of this complex (Fig. 8), which is consistent with the observation that a soluble construct, containing only the PSB domain, can be expressed and purified (21), and the purified protein has functional peptide-binding properties, as described for the PSB domains of C-P4H-I (24, 54) as well as for C-P4H-II (25). Superpositioning of the structures of the PSB–peptide complex (25) and Cr-P4H-peptide complex (27) on the model of C-P4H-II shows that the peptide-binding grooves of the PSB domain and the CAT domain are aligned, such that the C-terminal end of the peptide bound to the PSB domain points toward the N terminus of the peptide bound in the CAT active site (Fig. 8). However, there is a gap of about 32 Å between these two peptides, being the Cα–Cα distance between the C-terminal end of the PSB-bound peptide and the N-terminal end of the CAT domain–bound peptide. It is unclear if structural rearrangements occur in the C-P4H complex during the catalytic cycle that would align these grooves closer to each other. In any case, the conformational switch observed for Tyr140 of Cr-P4H (Fig. 7) is not possible in the current C-P4H-II model of the tetramer, as the flipped-out conformation of the corresponding residue (Tyr427) would overlap with the side chain of the conserved Tyr290 (located in the middle of the α12-helix). Therefore, the conformational flexibility properties of the βI-βII region of Cr-P4H suggest that structural rearrangements of the C-P4H complex during the catalytic cycle might be important, and the structure of the C-P4H-II-Δ281 complex represents just a snapshot of a structure that occurs during the catalytic cycle. More structural snapshots are required to fully understand the complicated catalytic cycle and the reaction mechanism of C-P4H.

## Concluding remarks

The structural studies of the C-P4H-II-Δ281 complex reveal the structure of the C-P4H CAT domain and its mode of interaction with the β/PDI subunit. This structure provides also important insight into the structural properties of the mature α_2_β_2_ C-P4H complex. The proposed model of the C-P4H tetramer is based on the crystal structure of the C-P4H-II-Δ281 complex, together with AlphaFold2 prediction of the structure of the α_2_-dimer. In this model, the CAT active site and its three active site loops face bulk solvent, whereas the β/PDI subunit is located on the backside of the CAT domain, having no interactions with any of the other domains. The PSB peptide-binding site of C-P4H is near the CAT peptide-binding site, suggesting that the procollagen chain can remain bound near the catalytic site, even if the substrate-binding tunnel of the catalytic site opens up at the end of the catalytic cycle, allowing for the hydroxylated peptide to be released from the catalytic site. In the catalytic cycle, the cofactor 2-oxoglutarate is converted into succinate, and opening up of the catalytic site (with the release of the hydroxylated peptide) is required for exchanging the succinate with 2-oxoglutarate after which the next reaction cycle can start. The notion that the procollagen substrate remains bound at the nearby PSB domain at the end of the catalytic cycle, and is not released in bulk solvent, is in line with the observed processivity properties of this enzyme (55). The model of the mature C-P4H tetramer shows that the active site of C-P4H is shaped by regions of each of the three domains of the α-subunit (including the α5-helix) as well as by a region (the α2-helix) of the other α-subunit of the α_2_-dimer. Therefore, our studies on the CAT mechanism of C-P4H will now focus on understanding the structural enzymological properties of the mature α_2_β_2_ C-P4H complex.

## Experimental procedures

### Cloning and construct preparation of the mature and truncated C-P4H variants

Two splice variants occur for the α-subunit of C-P4H-I (gene name: P4HA1_HUMAN) and C-P4H-II (gene name: P4HA2_HUMAN). These studies were done with the P13674-1 (UniProt) isoform-1 splice variant of C-P4H-I (in which exon 10 is expressed) (16) and with the O15460-1 (UniProt) isoform-IIb splice variant of C-P4H-II (in which exon 12b is expressed (17)). Codon-optimized genes of these variants were synthesized commercially (GenScript). The complementary DNA was inserted into the NdeI and BamHI cleavage sites. They were then cloned into a polycistronic CyDisCo plasmid already containing the codon-optimized PDI gene (the β-subunit of C-P4Hs, P07237 [UniProt]) and the yeast Erv1 gene (sulfhydryl oxidase) (39, 40). The N-terminal and C-terminal sequences of the expressed subunits are given in Table S1. The complete sequences of the expressed α-subunit and β-subunit are provided in Figure 1 and Figure S2, respectively. Second, various truncated C-P4H-II constructs lacking the N-terminal region of the α-subunit starting from Asp141, Asp282, Arg305, and Ala325 for C-P4H-II (Table S1 and Fig. 1) were also cloned into the polycistronic CyDisCo plasmid by using the corresponding codon-optimized α-subunit as a template and the primers shown in Table S1. These truncated C-P4H-II variants are referred to as C-P4H-II-Δ140, C-P4H-II-Δ281, C-P4H-II-Δ304, and C-P4H-II-Δ324, respectively. In all the constructs, the α-subunit was cloned to contain an N-terminal 6× His tag for convenient purification, as listed in Table S1. All cloning procedures were carried out using standard double digestion and ligation protocols. The positive colonies were screened by checking the size of the insert on an agarose gel, and the obtained clones were confirmed by sequencing using the Biocenter Oulu (BCO) DNA sequencing core facility.

### Expression and purification

The expression of the recombinant proteins was initiated by transforming the CyDisCo plasmid, which encodes also the α-subunit, the β/PDI subunit, and Erv1, into the K12 *E. coli* expression strain. In the protocol, on day 2, a single bacterial colony was first inoculated in Luria–Bertani medium containing 100 μg/ml ampicillin and grown overnight at 37 °C in a shaker. The overnight culture was diluted on day 3 (100-fold) in 1 l of Terrific broth medium supplemented with 100 μg/ml ampicillin and incubated at 30 °C with vigorous shaking (250 rpm) until the absorbance reached 0.6 to 0.7 at 600 nm. Then the culture was cooled to the induction temperature (20 °C), and the expression was induced by adding 0.5 mM IPTG and continued to grow overnight (∼17 h) at 20 °C (250 rpm). After expression, on day 4, the cells were harvested by centrifugation (10,800*g*, 30 min) and frozen with liquid nitrogen and stored at −70 °C for later use.

The protein purification was carried out at 4 °C. The frozen pellets, containing the recombinant proteins with the N-terminal 6× His tag in the α-subunit, were suspended in lysis buffer containing 50 mM Tris, pH 7.8, 100 mM NaCl, and 100 mM glycine, supplemented with SIGMAFAST protease inhibitor cocktail tablets (one tablet per 100 ml). The cell pellet was sonicated at 36% amplitude for 7 min with 2 s ON, 4 s OFF pulse on ice bath, and the supernatant was loaded onto a Talon immobilized metal-ion affinity chromatography column, washed with the lysis buffer, and eluted with the same buffer containing also 250 mM imidazole. The protein was then passed through a PD10 column pre-equilibrated with a buffer containing 10 mM Tris, pH 7.8, 50 mM NaCl, and 100 mM glycine. After this, the sample was subjected to anion exchange chromatography (HiTrap Q; GE Healthcare) connected to BioLogic DuoFlow purification device (Bio-Rad Laboratories, Inc) with the same buffer and eluted by using an NaCl gradient from 50 to 1000 mM. All fractions containing the protein (analyzed by using SDS-PAGE) were then pooled and concentrated with 50 kDa molecular weight cutoff Amicon Ultracentrifugal filters (Merck Millipore) before loading them on to a Superdex 200 10/300 GL column (bed volume 24 ml; GE Healthcare) pre-equilibrated with SEC buffer containing 50 mM Tris, pH 7.8, 50 mM NaCl, and 100 mM glycine. All fractions containing the protein were pooled and concentrated to about 3 to 10 mg/ml. Mostly, samples were frozen with liquid nitrogen in 100 μl aliquots (in SEC buffer) before being used for the subsequent experiments. The sample quality was monitored by SDS-PAGE analysis.

Peptide MS fingerprints and selected MSMS spectra were used to confirm the presence of the protein loaded on the SDS-PAGE gels after the purifications. Bands were excised, alkylated (DTT/iodoacetamide), and trypsinized using standard procedures. Peptides were eluted from the gel and measured on an UltrafleXtreme Maldi-Tof mass spectrometer (Bruker). Raw data were processed with BioTools and subjected to database search using Mascot (Matrix Science).

### SEC-MALS

The SEC-MALS analysis of the purified proteins (in SEC buffer) was carried out using a miniDAWN detector from Wyatt Technologies in on-line mode (connected to the Shimadzu HPLC system). Before entering the MALS detector, the sample flows through an Optilab refractive index detector from Wyatt Technologies. This is used to measure the concentration of the protein sample. Molecular mass and polydispersity measurements were carried out using the ASTRA software from Wyatt Technologies. About 30 to 50 μl of the purified (and frozen-thawed) sample was filtered and loaded with flow rate 500 μl/min onto the Superdex 200 Increase 10/300 GL column pre-equilibrated with SEC buffer (0.1 μm filtered and degassed).

### CD

Far-UV CD spectra of the purified proteins were recorded using a Chirascan CD spectrophotometer (Applied Photophysics Ltd) in a cuvette with 1 mm path length, using a wavelength range of 190 to 280 nm. The purified protein samples (in SEC buffer) were diluted to 0.1 mg/ml using Milli-Q water, and the final protein concentrations were determined from the absorbance values at 214 nm. The CD data were acquired every 1 nm with 0.5 s as an integration time and repeated three times with baseline correction. The samples were then heated from 22 to 94 °C using a Peltier temperature controller at a rate of 1 °C/min for obtaining a CD melting curve, recording at each temperature the CD spectrum using the same wavelength range as mentioned previously. The data collected between 72 and 94 °C were not used for the *T*_m_ calculations as the proteins started to aggregate. Data analysis was carried out using the Chirascan Pro-Data Viewer (Applied Photophysics) and CDNN (http://www.xn--gerald-bhm-lcb.de/download/cdnn). The direct CD measurements (θ; mdeg) were converted into mean residue molar ellipticity ([θ]MR) by Pro-Data Viewer. The *T*_m_ was calculated with the Global 3 software (Applied Photophysics) using a one-transition model and using the same wavelength range as aforementioned.

### Activity assays

Two assays were used to characterize the enzymatic properties of C-P4H-II and its truncated variants. First, the catalytic activity of the purified enzymes was measured by an indirect activity assay based on the hydroxylation-coupled decarboxylation of 2-oxo[1-^14^C]glutarate (PerkinElmer) (56). About 100 μM (PPG)_10_ peptide was used as a substrate. In addition, the activities were measured with a direct assay (56), based on measurement of the 4-hydroxy[^14^C]proline formation from [^14^C]proline-labeled procollagen substrate, consisting of nonhydroxylated α-chain of chicken type-I procollagen. The radioactivities were determined using a Tri-Carb 2900TR (PerkinElmer) liquid scintillation counter. The results were calculated as disintegrations per minute per enzyme active site, taking into account that the number of C-P4H active sites is two per mature C-P4H-II tetramer and one per heterodimer for the truncated variants.

### Peptide mapping MS experiments of mature C-P4H-II

The MS analyses were performed with native (untreated), DTT-reduced, and NEM-labeled samples of the mature C-P4H-II (in SEC buffer). The treatment with DTT reduces disulfide bridges, and the treatment with NEM will modify free SH groups. First, the protein solution was buffer-exchanged to 100 mM ammonium acetate (pH 6.8) by using a PD-10 column (Cytiva). In the next step, three 50-μl aliquots were taken from the C-P4H-II sample (3.5 μM), the first being the native sample, the second reduced with DTT (1 mM, 1 h at room temperature), and the third treated with NEM (1 mM, 1 h at room temperature). In the third step, each sample was diluted (1:1, v/v) with ammonium acetate (pH 2.6) (to reduce pH to around 4.1 to facilitate on-line pepsin digestion and to prevent cysteine oxidation). The on-line pepsin digestion tubing was prepared as follows (57): a 50-μl PEEK tubing was first washed with 1% formic acid, followed by filling the tubing with a pepsin solution (1 mg/ml pepsin in 1% formic acid). After an overnight incubation at 4 °C, the tubing was washed once with a small volume of water. The tubing, with the immobilized pepsin, was then connected directly to the ion source, and the protein solutions were infused through the tubing at room temperature at a flow rate of 2 μl/min using a syringe pump, resulting in an approximately 30 min digestion time. The mass spectra were measured using a 12-T Bruker solariX XR Fourier transform ion cyclotron resonance MS (Bruker Daltonics GmbH), equipped with a dynamically harmonized Paracell and an Apollo-II electrospray ionization source, operated in the positive-ion mode. The ion source temperature was set to 200 °C, and the nebulizing and drying gases were set to 1 bar and 4 l/min, respectively. The data were acquired with ftmsControl 2.1 (Bruker Daltonics GmbH) and further processed using DataAnalysis 4.4 software (Bruker Daltonics GmbH). The mass spectra were acquired using a one molecular weight dataset size with 256 scans summed for the final spectra using an *m/z* range of 387 to 5000. The mass spectra were further internally recalibrated using monoisotopic masses of the selected peptides, whose sequences were confirmed by MS/MS (collision-induced dissociation) measurements. The peptide monoisotopic masses were obtained by using a built-in SNAP2 peak picking algorithm and were then searched against the C-P4H-II sequence with GPMAW 9.2 software (Lighthouse Data) and ProteinProspector (https://prospector.ucsf.edu/prospector/mshome). Because of the broad cleavage specificity of pepsin, the peptide mapping was performed as follows. The peptides with a single hit were assigned first, and the observed cleavage sites of these peptides were then used to assign the other peptides with multiple possible locations. No restrictions were applied to the digestion sites, that is, nonspecific cleavage was used, and only the peptides with less than 10 ppm mass error were considered. All possible disulfide linkages were initially considered in the search of the peptides including intersubunit and intrasubunit ones by searching α- and β-subunits simultaneously. The search was repeated by limiting the disulfide pairing to those observed in the crystal structure. The identified peptides as well as the sequence coverages in each of the three experiments are listed in Table S2. A summary is provided in Table S3.

### Protein crystallography studies

Extensive crystallization screening was performed with the C-P4H-II-Δ281 complex. After SEC, the protein (in SEC buffer) was concentrated to 11 mg/ml by using 50 K Amicon Ultra-15 Centrifugal Filter concentrator (Merck). The protein was subjected to extensive crystallization screening by using the equipment of the BCO, University of Oulu Structural Biology core facility. Initial screening for crystallization was carried out by the sitting drop vapor diffusion method using in-house screens in IQ 96-well triple sitting drop plates (TTP Labtech) at two different temperatures (22 and 4 °C). The protein and precipitant solutions were mixed together in three volume ratios (1:2, 1:1, and 2:1) in a final volume of 300 nl with the help of the Mosquito LCP nanodispenser (TTP Labtech). The plates were imaged using Formulatrix Rock Imagers RI54 (at 22 °C) and RI27 (at 4 °C). The crystallization results were monitored with the IceBear software expert system (58).

The first, poorly diffracting, crystals of the CP4H-II-Δ281 complex were obtained at 4 °C using a well solution of 0.1 M MES, pH 6.0, containing 20% (w/v) PEG4000, 0.2 M lithium sulfate (ProPlex screen HT-96; Molecular Dimensions) at a volume ratio of 1:2 for the protein and well solutions, respectively. In subsequent purifications, the SEC buffer of the last step of the purification protocol was supplemented with 1 mM EDTA, and in further crystallization experiments, several additive screens were tested to improve the crystal quality. The best crystals were eventually obtained at 4 °C by mixing equal volumes of a protein solution of 16.5 mg/ml in 50 mM Tris–HCl, pH 7.8 (at 4 °C), 50 mM NaCl, 100 mM glycine, 1 mM EDTA, with a well solution of 0.1 M MES, pH 6.0 (at 4 °C), 20% (w/v) PEG4000, 0.2 M lithium sulfate, and 3% methanol. The initial crystal testing was done in the BCO Structural Biology core facility/Biocenter Finland national data collection core facility, using the in-house Microstar X8 Proteum X-ray generator (Bruker). Crystals were cryoprotected, before cryocooling by immersing in liquid nitrogen, by quick transfer (few seconds of equilibration) in a cryobuffer (0.1 M MES, pH 6.0 buffer with 15% ethylene glycol, 20% [w/v] PEG4000, and 0.2 M lithium sulfate). All the crystal fishing and cryocooling protocols were performed in the cold room at 4 °C. Many crystals were tested with the home source X-ray generator, and the best crystals (showing diffraction between 6 Å and 9 Å resolution) were stored and shipped to synchrotrons (ESRF [beamline ID29], PETRA III [DESY, EMBL Hamburg, beamline P14], and Diamond Light Source, beamlines I03, I04, and VMXi). The best dataset (3.8 Å resolution) was collected at the beamline P14, PETRA III (DESY), EMBL Hamburg. The X-ray images were processed by XDS (59), and the data were scaled using Aimless (60) of the CCP4 package (61). The data processing statistics are listed in Table 2.

The structure of the C-P4H-II-Δ281 complex was solved by molecular replacement, using Phaser (62) and Molrep (63). In the first step, the crystal structure of Cr-P4H (PDB entry: 2JIG) (26) was used as a model to find two copies of the CAT domain. The two copies formed a dimer with the orientation of the local twofold axis consistent with the self-rotation function calculated using Molrep (63). Such a confirmation was important at this point because, with low-resolution data, incorrect molecular replacement solutions with a good contrast are not unusual, whereas electron density maps from a partial structure are not very instructive. In the next steps, two copies of each of the **a**, **a'**, and **b’** (in this order) domains of the β/PDI subunit were positioned using Phaser. The **a** and **a’** domains were found by using as search models the structures of these domains, as present in the reduced (“open”) form of human PDI (PDB entry: 4EL1) (35), and the **b’** domain was found by using the structure of this domain in the β/PDI subunit of the MTP complex (PDB entry: 6I7S (36)). The PDI domains were located around the central CAT domain dimer and followed the same local twofold symmetry as the previously positioned CAT domains. The initial refinement trials with the partial model with Phenix (64), Refmac5 (65), and Lowrestr (66) gave *R*_work_ and *R*_free_ of around 40.0 and 45.0%, respectively. The **b** domain was not found by molecular replacement searches, but it was positioned manually in the weak electron density map. First, the PDI structure (PDB entry: 4EL1) was superposed onto the already positioned **a** and **b'** domains using Coot (67), and subsequently, the **b** domain position was optimized manually. The complete structure was then refined using the BUSTER-TNT software (Global Phasing Limited) (68), and model corrections were done using Coot (67). In this model, the linker region at the N terminus of the α-subunit construct and the C-terminal tail of the α-subunit were built as a polyalanine model. *R*_work_ and *R*_free_ were 33.8% and 35.4%, respectively.

At this stage, the predicted models of the AlphaFold2 (38, 69) and Robetta (53) structure prediction machine learning software became available. Both models predicted the same conformation for the regions that were built as polyalanine stretches of the intermediately refined C-P4H-II-Δ281 structure, and these predicted conformations were also the same as built in this intermediate structure. Subsequently, the sequences of the predicted model were assigned to the polyalanine stretches of the α-subunits, and the structure refinement was revisited with AlphaFold2 models for CAT domain and β/PDI subunits being used for generating additional geometric restraints.

The final structure is the result of three additional series of refinement with BUSTER, Refmac5, and again BUSTER. Model correction in Coot was done using BUSTER maps, whereas the intermediate Refmac5 series helped pushing optimization forward. *R*_work_/*R*_free_ in the last BUSTER refinement run before and in the first BUSTER refinement run after the Refmac5 series were 27.8%/30.4% and 26.0%/28.0%, respectively. The slow progress of the refinement is possibly associated with the inaccuracy of the initial positioning of the domains of the less ordered β/PDI subunits and smaller gradients for their atoms during refinement (the centers of the individual domains of the β/PDI subunits, calculated as averaged coordinates of Cα-atoms, moved during the three series of refinement by 0.17 to 0.52 Å, whereas the centers of the two CAT domains moved by only 0.12 and 0.13 Å).

The refinement protocols for BUSTER and Refmac5 were similar and included TLS refinement (one “big” cycle in case of BUSTER or 10 cycles in case of Refmac5) followed by restrained refinement of atomic coordinates and the overall *B*-factor (5–7 “big” cycles or 20 cycles). In both protocols, the relative weight between X-ray and geometry terms was estimated by the respective program. *B*-factors of all atoms were reset to 50 Å^2^ prior to each refinement run. In the TLS refinement cycles, the entire CAT domains and individual domains of the β/PDI subunits were assigned as TLS groups (based on the results of preliminary trials, where subunit-based definition of TLS groups gave higher *R*-factors). TLS parameters were recalculated from scratch in each run. The AlphaFold2 models of the CAT domain and the β/PDI subunit were generated using AlphaFold2, as implemented at the EBI (69), and these models were used as reference structure for the BUSTER refinement, being used by BUSTER to internally generate local structural similarity restraints (LSSRs) (70). In case of Refmac5, the external restraints (similar to LSSR but named differently) were generated explicitly using ProSMART (71), and weights were customized for repulsion restraints (increased from 1 to 2) and X-ray term (fixed to 0.0004). The final BUSTER model was validated with a Refmac5 test refinement cycle, using the same parameters but without external restraints. The overall rmsd Cα of 0.15 Å (0.14–0.17 Å for individual subunits) (with maximum Cα difference of 0.5 Å) for Gly298 of subunit B when comparing the final model (BUSTER with LSSR) and the control model (Refmac5 without external restraints) show that LSSR did not introduce any considerable model bias. Sharpened maps for model building and structure analysis were calculated using the default map coefficients of BUSTER.

The quality of the structure was monitored using the validation tools in Coot, Molprobity (72), and the wwPDB validation server throughout the refinement. The refinement statistics of the final model (*R*_work_ = 24.4%, *R*_free_ = 27.8%, as calculated by BUSTER) are listed in Table 2. The structures of the final model of both CAT domains as well as of the **a’** and **b’** domains of both β/PDI subunits are nicely defined by the electron density map (Figs. S5–S7). In addition, the structures of the PDI **a** domains are well defined, and the two least well-defined PDI **b** domains still unambiguously match the electron density map.

## Data availability

The structure presented in this article has been deposited in the PDB with the code 7ZSC.

## Supporting information

This article contains supporting information (26, 27, 35, 37, 41, 42, 43, 44).

## Conflict of interest

A patent for the production system used to make the protein for structural studies using sulfhydryl oxidases in the cytoplasm of *E. coli* is held by the University of Oulu: Method for producing natively folded proteins in a prokaryotic host (Patent number: 9238817; date of patent: January 19, 2016). Inventor: L. W. R.

J. M. owns equity in FibroGen, Inc, which develops HIF-P4H inhibitors as potential therapeutics. This company supports research in the J. M. group.

All the other authors declare that they have no conflicts of interest with the contents of this article.
