# Supplementary material for: Crystal structure of the collagen prolyl 4-hydroxylase (C-P4H) catalytic domain complexed with PDI: Toward a model of the C-P4H α2β2 tetramer
Source: J Biol Chem. 2022 Oct 18;298(12):102614. doi: 10.1016/j.jbc.2022.102614 (PMC9676403; doi:10.1016/j.jbc.2022.102614)
Supplement: Supporting information [file mmc1.pdf]

Supporting Information

Crystal structure of the collagen prolyl 4-hydroxylase (C-P4H) catalytic domain complexed with PDI: towards a model of the C-P4H $\alpha_2\beta_2$ tetramer

Abhinandan V. Murthy[1,2#], Ramita Sulu[1#], Andrey Lebedev[3], Antti M. Salo[1,2], Kati Korhonen[1], Rajaram Venkatesan[1], Hongmin Tu[2], Ulrich Bergmann[2], Janne Jänis[4], Mikko Laitaoja[4], Lloyd Ruddock[1], Johanna Myllyharju[1,2], M. Kristian Koski[1,2*], Rik K. Wierenga[1*]

#Abhinandan V. Murthy and #Ramita Sulu are joint first authors. *M. Kristian Koski (kristian.koski@oulu.fi) and *Rik K. Wierenga (rik.wierenga@oulu.fi) are corresponding authors.

[1] Faculty of Biochemistry and Molecular Medicine, University of Oulu, P.O. Box 5400, FI-90014 University of Oulu, Finland
[2] Biocenter Oulu, University of Oulu, P.O. Box 5000, FI-90014 University of Oulu, Finland
[3] Scientific Computing Department, STFC Rutherford Appleton Lab., RCaH, Harwell Campus, Didcot OX11 0FA, UK
[4] Department of Chemistry, University of Eastern Finland, P.O. Box 111, 80101 Joensuu, Finland

Current address Abhinandan V. Murthy: Translational Cancer Biology Program, Research Programs Unit, Biomedicum Helsinki, University of Helsinki, FI-00014 Helsinki, Finland

Table of Contents:

Table S1. List of primers used in this study and the N-terminal and C-terminal sequences (including the tags, if present) of the constructs.

Table S2. The peptides observed in the mass spectrometry mapping experiment with the purified mature C-P4H-II complex, are listed for each of the three experiments

Table S3. Summary of the mass spectrometry peptide mapping experiments with mature C-P4H-II

Figure S1. Alignment of sequences of the catalytic domain of prolyl 4-hydroxylases and their splicing variants

Figure S2. Alignment of the sequence of human PDI and some of its homologs

Figure S3. SEC-MALS analysis of mature C-P4H-II and its two truncated variants

Figure S4. Protein conformation and thermostability analysis by CD spectroscopy

Figure S5. The 2Fo-Fc electron density map of the CAT-PDI heterodimer complex and of regions of the CAT domain and the β/PDI subunit

Figure S6. The 2Fo-Fc electron density map at the interaction site of the CAT domain with the **a'** domain of the β/PDI subunit

Figure S7. The 2Fo-Fc electron density map at the interaction site of the CAT domain with the **a** domain of the β/PDI subunit

Figure S8. The thioredoxin fold

Figure S9. Electron density maps of the regions with the inter-subunit disulfide bridges

Figure S10. Stereo view of the structural details of the interaction sites of the CAT domain with the **a'** and **a** domains of the β/PDI subunit

Figure S11. Comparison of the assembly (A) of PDI with the CAT domain of the C-P4H-II-Δ281 complex, (B) of PDI with the α-subunit of MTP and (C) of ERp57 with tapasin

Figure S12. The interactions of the CAT domain in the hydrophobic pocket of the **b'** domain of the β/PDI subunit

Figure S13. Comparison of the structures of the CAT domains of C-P4H-II-Δ281 and Cr-P4H.

Figure S14. pLDDT plot calculated by AlphaFold2 for the structure prediction of the α_2-dimer of the mature C-P4H-II

Supplementary Tables

Table S1. List of primers used in this study and the N-terminal and C-terminal sequences (including the tags, if present) of the used constructs

Construct	Primers used (5' --> 3') [1]	Sequence of the N-terminus and C-terminus of the construct
mature CP4H-I (α)	not applicable	MHHHHHHMHPGFFTSIG……….TLSELE
mature CP4H-II (α)	not applicable	MHHHHHHMEFFTSIGHM………GSTEVD
C-P4H-II-Δ140 (α)	F: TTT **CATATG** GATACCTATCGCCTG<br>R: AAA **GGATCC** TTAATCCACTTCGGT	MHHHHHHMDTYRLDTG………GSTEVD
C-P4H-II-Δ281 (α)	F: TTT **CATATG** GATTATCTGCCGGAA<br>R: AAA **GGATCC** TTAATCCACTTCGGT	MHHHHHHMDYLPERDV………GSTEVD
C-P4H-II-Δ304 (α)	F: TTT **CATATG** CGCCAGAAACGC<br>R: AAA **GGATCC** TTAATCCACTTCGGT	MHHHHHHMRQKRLFCR………GSTEVD
C-P4H-II-Δ324 (α)	F: TTT **CATATG** GCGCCGTTTAAAGAA<br>R: AAA **GGATCC** TTAATCCACTTCGG	MHHHHHHMAPFKEEDE………GSTEVD
β/PDI	not applicable	MDAP………………………………..KDEL

[1] Restriction sites of the primers are marked in bold.

Table S2. The peptides observed in the mass spectrometry mapping experiment with the purified mature C-P4H-II complex, are listed for each of the three experiments

The information concerning the peptides found in the peptide mapping experiments with the purified mature C-P4H-II can be found in a separate file:

Mass-Spectrometry-Peptide-Mapping-C-P4H-II.pdf

Table S3. **Summary of the mass spectrometry peptide mapping experiments with mature C-P4H-II**[1]

	**native**	**DTT treatment**	**NEM-labelling**	**Comments**
**α-subunit**				
Cys169, C-P4H-II	Free thiol peptide is not found	Free thiol peptide is not found	NEM labelled peptide is found	
Cys294, C-P4H-II	Free thiol peptide is not found	Free thiol peptide is found	NEM labelled peptide is not found	can be part of a disulfide
Cys311, C-P4H-II	Free thiol peptide is not found	Free thiol peptide is found	NEM labelled peptide is found	
Cys504, C-P4H-II	Free thiol peptide is not found	Free thiol peptide is found	NEM labelled peptide is not found	can be part of a disulfide
Cys510, C-P4H-II	Free thiol peptide is not found	Free thiol peptide is found	NEM labelled peptide is found	
Cys529, C-P4H-II	Free thiol peptide is found	Free thiol peptide is found	NEM labelled peptide is found	
**β/PDI-subunit**				
Cys53, C-P4H-II	disulfide peptide is found	Free thiol peptide is found	NEM labelled peptide is not found	disulfide bridge with Cys56
Cys56, C-P4H-II	disulfide peptide is found	Free thiol peptide is found	NEM labelled peptide is not found	disulfide bridge with Cys53
Cys312, C-P4H-II	Free thiol peptide is not found	Free thiol peptide is found	NEM labelled peptide is found	
Cys343, C-P4H-II	Free thiol peptide is not found	Free thiol peptide is found	NEM labelled peptide is found	
Cys397, C-P4H-II	disulfide peptide is found	Free thiol peptide is found	NEM labelled peptide is not found	disulfide bridge with Cys400
Cys400, C-P4H-II	disulfide peptide is found	Free thiol peptide is found	NEM labelled peptide is not found	disulfide bridge with Cys397

[1] This table summarizes the information concerning cysteine containing peptides that were found (or not found) in the three different peptide mapping experiments with mature human C-P4H-II. The peptide information included in the table concerns either free thiol peptides, disulfide peptides or NEM labeled peptides. The peptide mapping did not show any evidence for the presence of peptides in which the cysteines were oxidized to a hydroxycysteine or to any higher oxidation state.

Supplementary figures

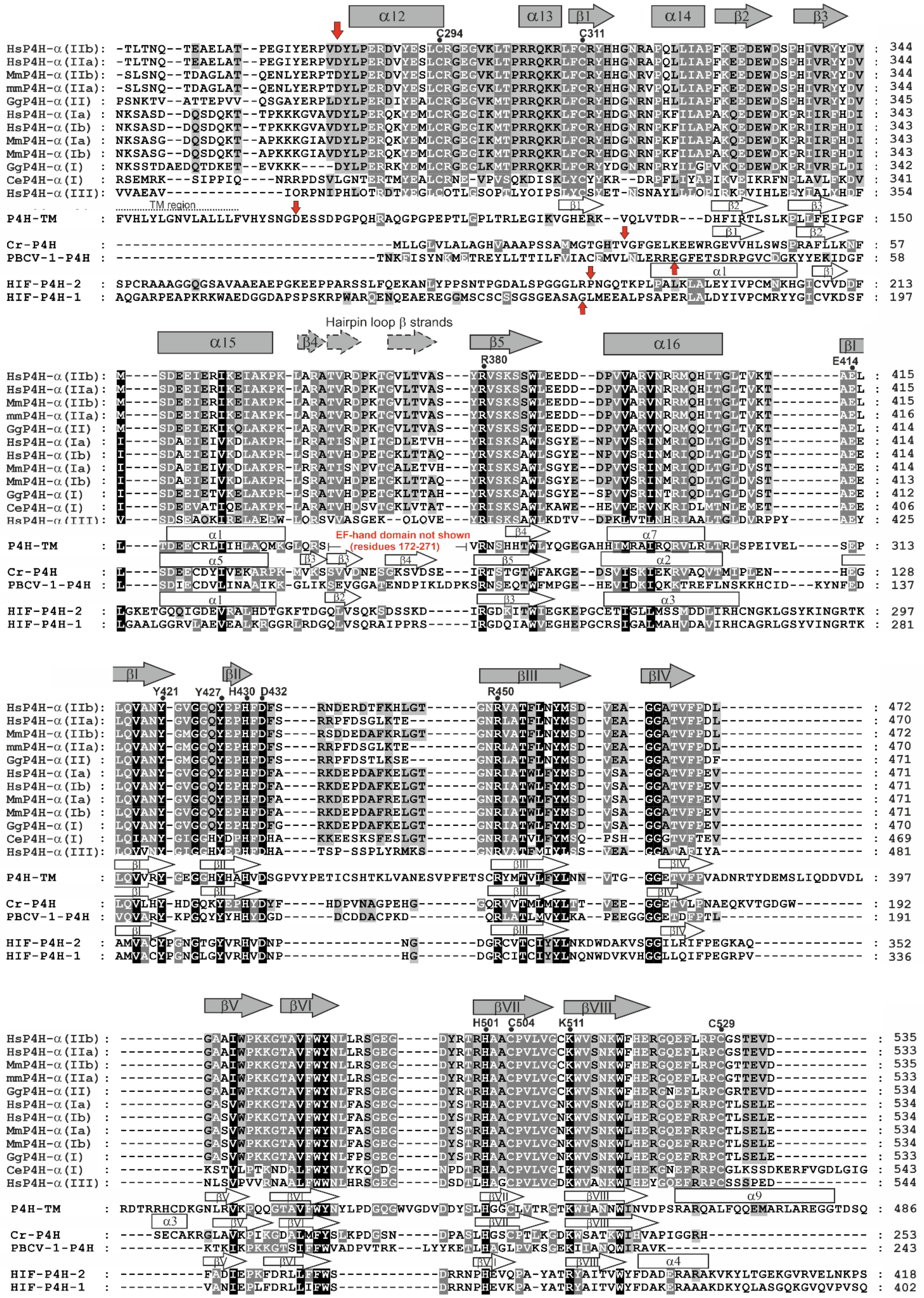

HsP4H-α(IIb)
HsP4H-α(IIa)
MmP4H-α(IIb)
mmP4H-α(IIa)
GgP4H-α(II)
HsP4H-α(Ia)
HsP4H-α(Ib)
MmP4H-α(Ia)
MmP4H-α(Ib)
GgP4H-α(I)
CeP4H-α(I)
HsP4H-α(III)
P4H-TM
Cr-P4H
PBCV-1-P4H
HIF-P4H-2
HIF-P4H-1
TM region
Hairpin loop β strands
EF-hand domain not shown
(residues 172-271)
C294
C311
R380
E414
Y421
Y427
H430
D432
R450
H501
C504
K511
C529

Figure S1. Alignment of sequences of the catalytic domain of prolyl 4-hydroxylases and their splicing variants. HsP4H-α(IIb), HsP4H-α(IIa) concern the sequences of human C-P4H-II with the 12b exon and 12a exon, respectively. Similarly, HsP4H-α(Ia), HsP4H-α(Ib) concern the sequences of human C-P4H-I with the exon 10 and exon 9 sequences, respectively. The studies reported here have been done with HsP4H-α(IIb) and HsP4H-α(Ia). MmP4H, GgP4H and CeP4H refer to the mouse, chicken and *Caenorhabditis elegans* C-P4H sequences. The alignment is further extended with the sequences of five other P4Hs of which structures are available, being P4H-TM (PDB entry 6TP5), Cr-P4H (PDB entry 3GZE), PBCV-1-P4H (PDB entry C5T), HIF-P4H-2 (PDB entry 3HQR) and HIF-P4H-1 (PDB entry 5V1B) and their secondary structure elements are shown above each sequence. P4H-TM is the human membrane anchored P4H (41). Cr-P4H is the isoform 1 of the *Chlamydomonas reinhardtii* P4H (26, 27), PBCV-1-P4H is the viral P4H of *Paramecium Bursaria* Chlorella virus (44). HIF-P4H is the human hypoxia-inducible-factor P4H (42, 43). The eight β-strands of the DSBH-fold are numbered using roman numerals, βI-βVIII (see also **Fig. 3C**). The residues of the CAT domain that bind the Fe(II) ion, 2-oxoglutarate and the key residues for substrate binding, as well as the conserved cysteines, are highlighted. Vertical arrows show the first residue of each construct used in the crystallization studies. The sequence of the 100 residue long EF-hand domain, unique for P4H-TM, is not shown. Full sequence conservation is highlighted as white letters on black background.

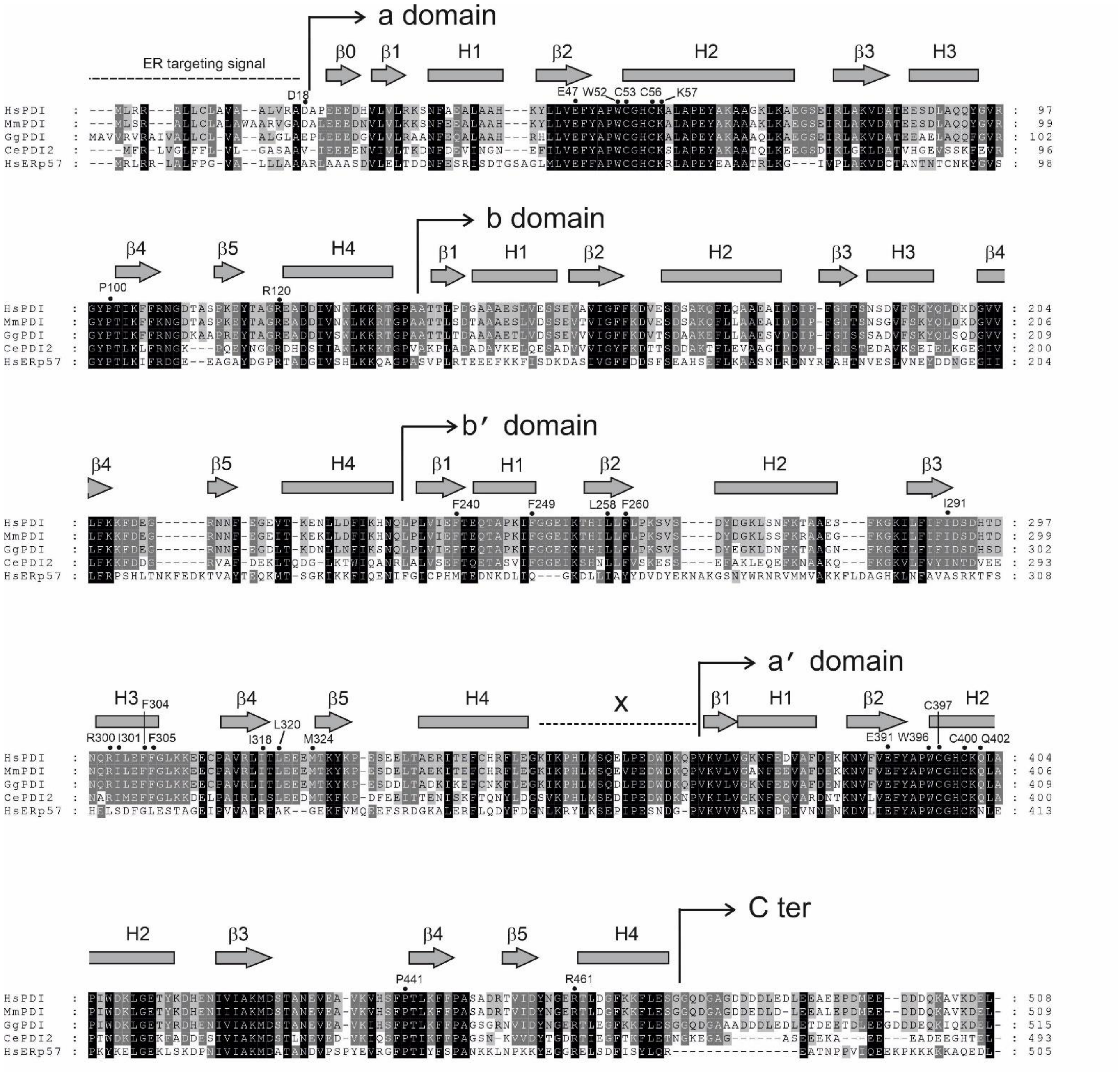

Figure S2. Alignment of the sequence of human PDI and some of its homologs. The sequences include the N-terminal endoplasmic reticulum targeting sequence, shown with a dashed line above the sequences. The HsPDI (human), MmPDI (mouse), GgPDI (chicken), CePDI2 (*C. elegans*) concern PDI isoforms which are known to form complexes with the α-subunit of prolyl 4-hydroxylase. Crystal structures are known of HsPDI (35) and HsERp57 (37). The CGHC-sequence motif is at the N-terminus of helix H2 of domain **a** and **a'**. The construct starts at Asp18 (**Table S1**). The secondary structure elements are labelled similarly for each of the four thioredoxin domains, as also shown in **Fig. S8**. Important residues, that are discussed in the text, are highlighted above the sequence alignment.

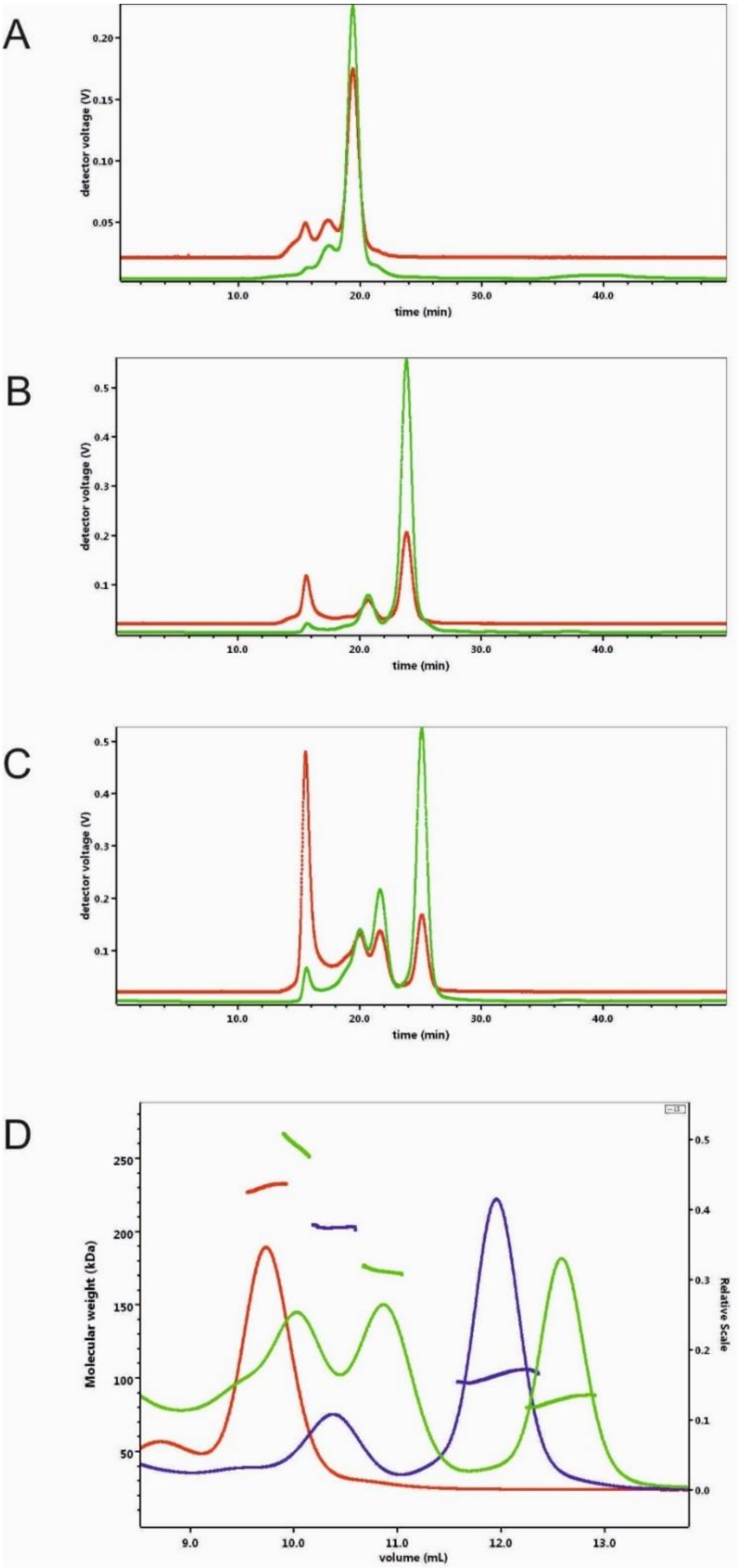

Figure S3. SEC-MALS analysis of mature C-P4H-II and its two truncated variants. SEC chromatograms of (A) mature C-P4H-II, (B) C-P4H-II-Δ140 and (C) C-P4H-II-Δ281. Shown are the UV curve (green) obtained from the SPD-M20A diode array detector of the high-performance-liquid-chromatography system (Shimadzu Corp.) and the light scattering signal (angle= 90°) of the miniDAWN MALS detector (Wyatt technologies). (D) The light scattering signals of all three runs are shown (mature C-P4H-II, red; C-P4H-II-Δ140, blue; C-P4H-II-Δ281, green). In addition, the molecular mass distribution profiles of each run are shown for the peak regions (horizontal lines). The C-P4H-II-Δ281 sample contains heterodimers, as well as higher oligomers (dimers and trimers of the heterodimers). Such higher oligomers are not observed in freshly prepared samples.

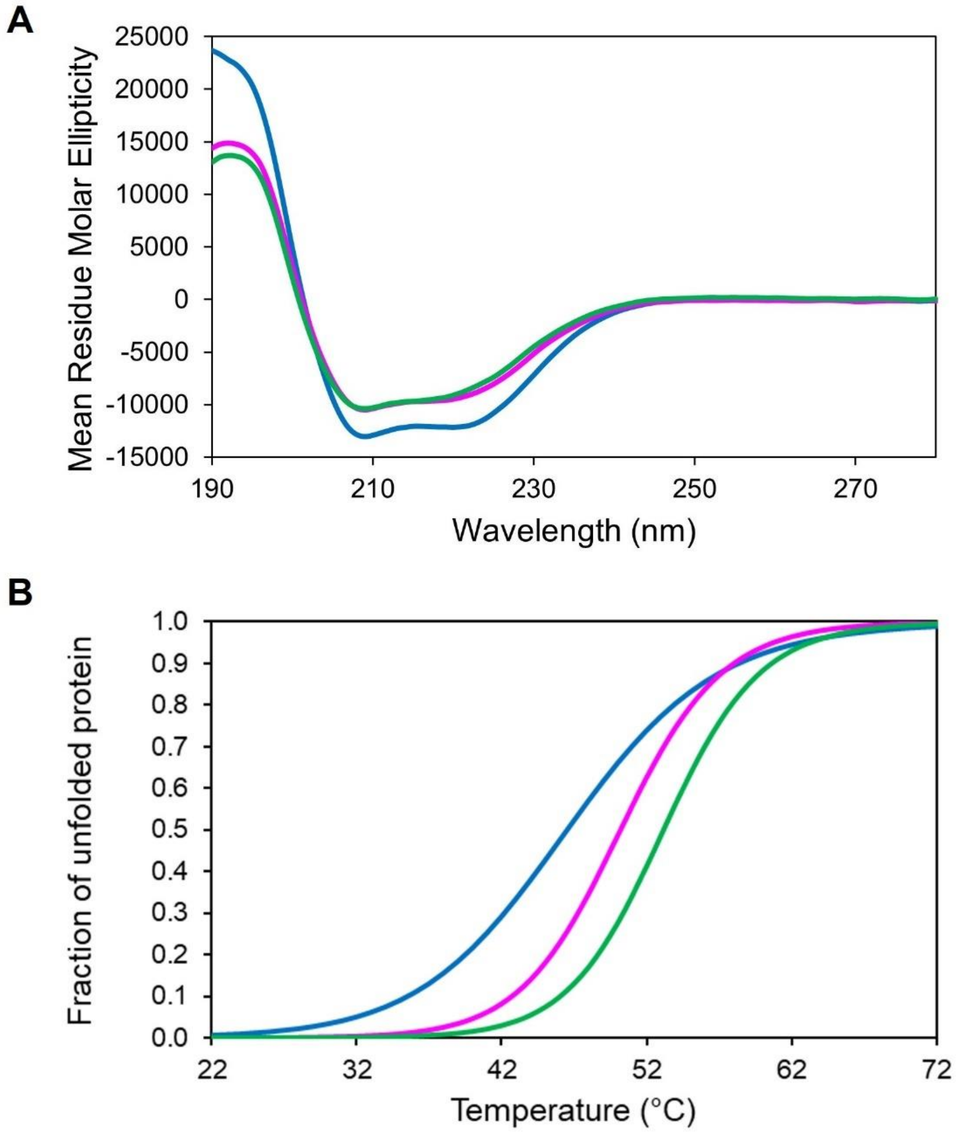

Figure S4. Protein conformation and thermostability analysis by CD spectroscopy. (A) Processed circular dichroism spectra of mature C-P4H-II (blue), C-P4H-II-Δ140 (magenta) and C-P4H-II- Δ281 (green). (B) Calculated melting curves of C-P4H-II (blue), C-P4H-II-Δ140 (magenta) and C-P4H-II-Δ281 (green). The experimental details are provided in the CD section of the Experimental procedures. The predicted secondary structure content as calculated by CDNN (http://www.xn--gerald-bhm-lcb.de/download/cdnn) from the CD spectra for the mature C-P4H-II is: α-helix 39.1 %, antiparallel β sheet 6.9 %, parallel β sheet 7.5 %, β turn 15.9 %, and random coil 29.4 %. For the C-P4H-II-Δ140 construct the secondary structure content is predicted as α-helix 29.6 %, antiparallel β sheet 11.3 %, parallel β sheet 10.2 %, β turn 16.5 % and random coil 33.3 %. For the C-P4H-II-Δ281 construct it is α-helix 27.6 %, antiparallel β sheet 9.3 %, parallel β sheet 9.6 %, β turn 17.6 %, and random coil 36.7 %.

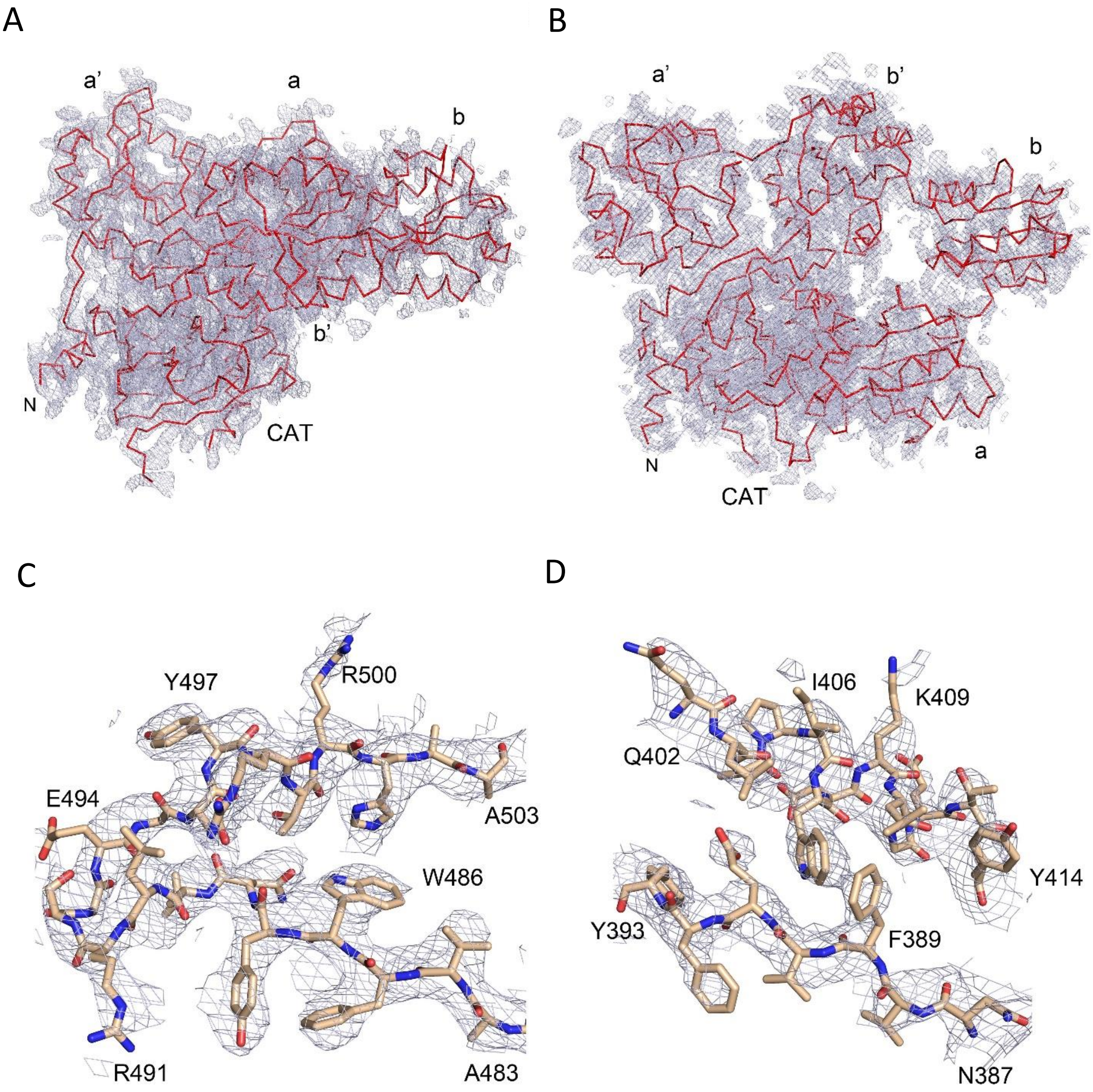

Figure S5. The 2Fo-Fc electron density map of the CAT-PDI heterodimer of the C-P4H-II-Δ281 complex and of regions of the CAT domain and the β/PDI subunit. (A) The CAT-PDI complex (chains A and C). Same view as **Fig. 3a**. (B) The CAT-PDI complex (chains A and C). Same view as **Fig. 3b**. (C) The βVI-βVII region of the CAT domain (chain A) is shown. (D) The β2 and helix H2 regions of the **a'** domain of the β/PDI subunit (chain C) is shown. The contour level is the same in all panels (1.0 sigma). In panels A and B the N-terminus of the CAT domain is labeled as "N" and the electron density for the N-terminal region of the CAT domain is visible. In panel B the electron density map visualizes the shape of the CAT domain and the four β/PDI domains.

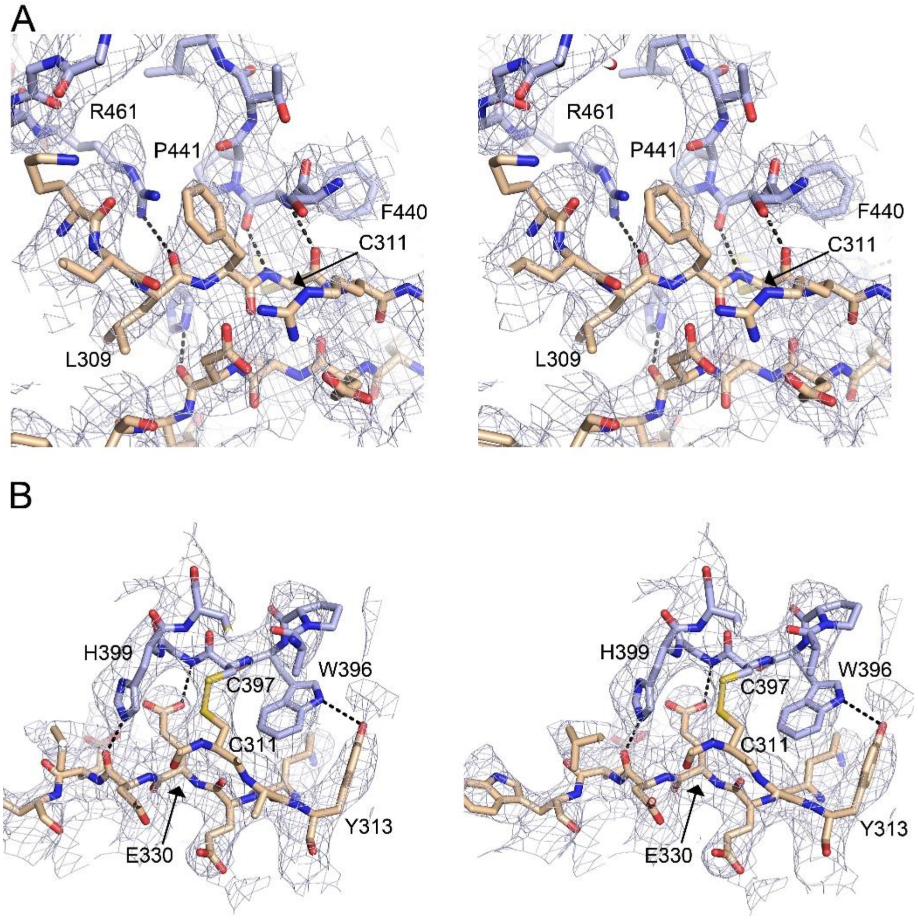

Figure S6. The 2Fo-Fc electron density maps at the interaction site of the CAT domain (chain A) with the a' domain of the β/PDI subunit (chain C). Stereo views. In each of the panels the contour level is 1.0 sigma and the view is similar as in **Fig. 4**. Dotted lines visualize hydrogen bond interactions. (A) The interactions of the *cis*-(Phe440-Pro441)-peptide region. (B) The interactions of the CGHC loop (including Trp396, Cys397, His399). Details of these interactions are described in the legend of Fig. **S10**.

A

B

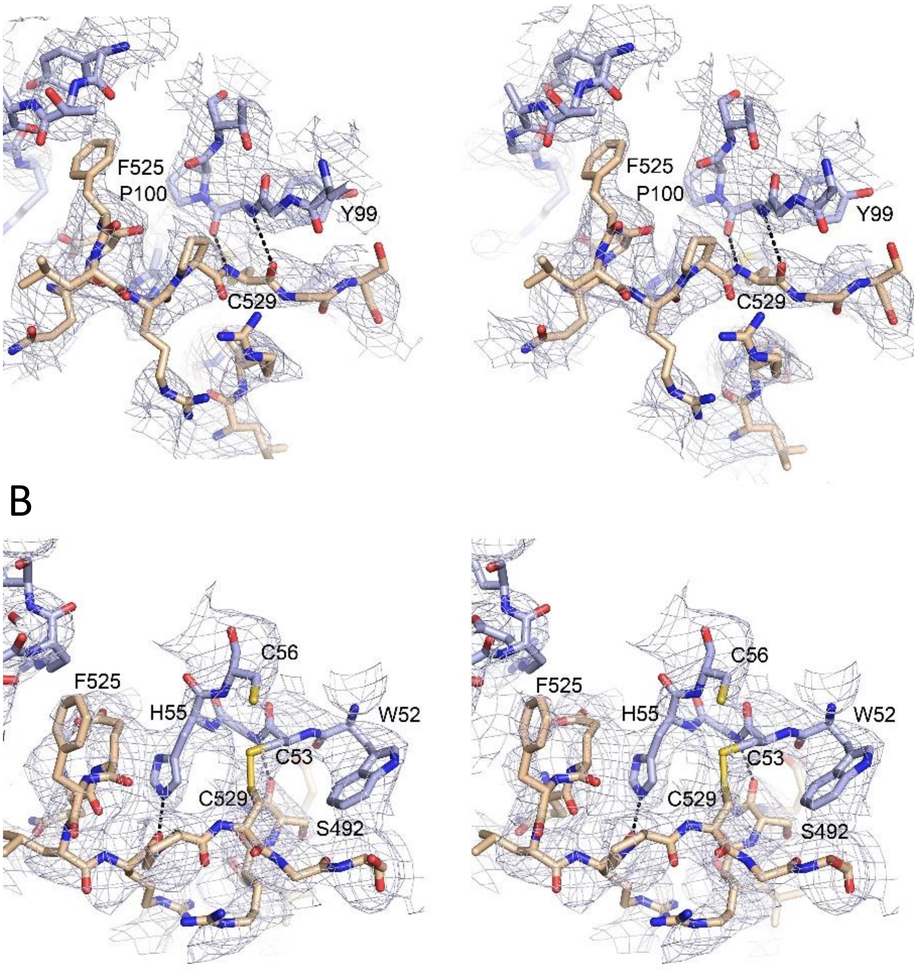

Figure S7. The 2Fo-Fc electron density maps at the interaction site of the CAT domain (chain A) with the a domain of the β/PDI subunit (chain C). Stereo views. In each of the panels the contour level is 1.0 sigma and the view is similar as in **Fig. 4**. Dotted lines visualize hydrogen bond interactions. (A) The interactions of *cis*-(Tyr99-Pro100)-peptide region. (B) The interactions of the CGHC loop (including Trp52, Cys53, His55). Details of these interactions are described in the legend of **Fig. S10**.

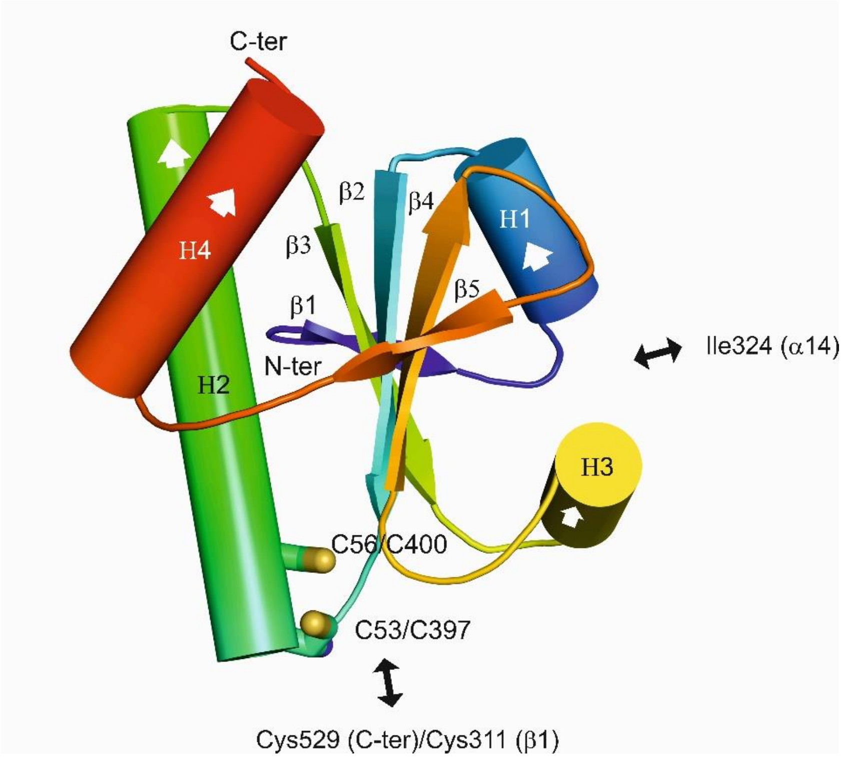

Figure S8. The thioredoxin fold, taking the a domain of human PDI as an example. The cysteines of the CGHC sequence motif are shown as stick models and labeled as C53/C397 (the proximal cysteine) and C56/C400 (the distal cysteine) of the **a**/**a'** domains of PDI. This motif is located in the loop between β2 and H2. The conserved *cis*-Pro (Pro100/Pro441) is in the loop between H3 and β4. The ribbon diagram is color coded by color ramping from N-terminus (dark blue) to C-terminus (red). The interaction sites with the CAT domain of the α-subunit are indicated by arrows. The **a** (C53) and **a'** (C397) interaction sites are with Cys529 and Cys311 of the CAT domain, respectively, whereas the **b'** interaction site is with Ile324 of the CAT domain. In the **a'** domain the H3 region is not helical, but instead adopts an extended loop conformation.

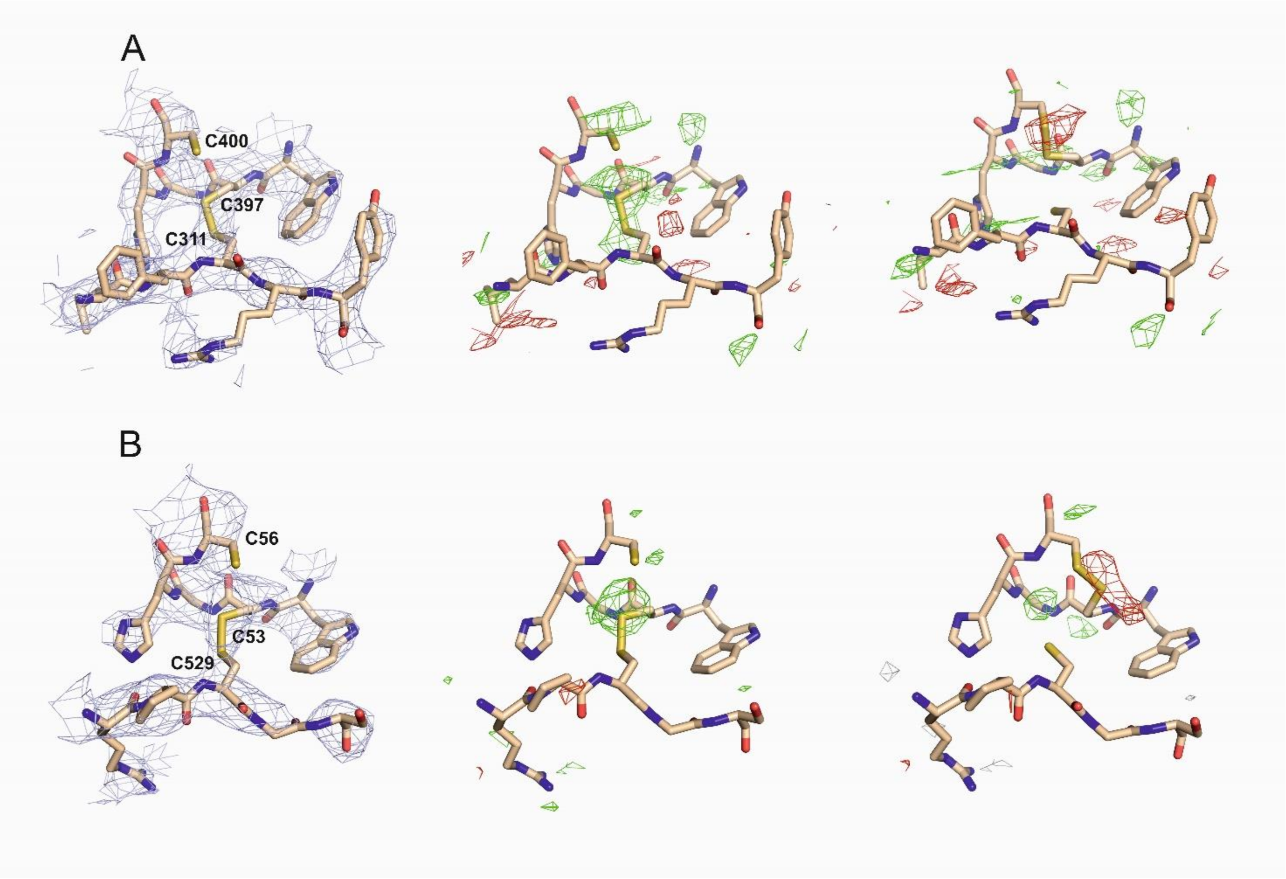

Figure S9. Electron density maps of the regions with the inter-subunit disulfide bridges. (A) Electron density maps of the inter-disulfide bridges of Cys311(CAT, chain A)-Cys397(PDI, chain C) and (B) of Cys529(CAT, chain A)-Cys53(PDI, chain C). On the left is shown the final 2Fo-Fc map (contoured at 1.2 sigma) calculated with the final coordinate set. The superimposed final model is also shown. In the middle panel is shown the omit Fo-Fc map (negative density, red (contour level -2.5 sigma) and positive density, green (contour level +2.5 sigma)) calculated after refinement using the final model in which the 12 cysteine sulfurs of the four inter-subunit disulfide bridges plus the distal PDI cysteines were deleted. Shown is also the superimposed final model. On the right is shown the Fo-Fc map (negative density, red (contour level -2.5 sigma) and positive density, green (contour level +2.5 sigma)) calculated from a refined model in which the four inter-subunit disulfide bridges were switched. The superimposed switched model is also shown.

A

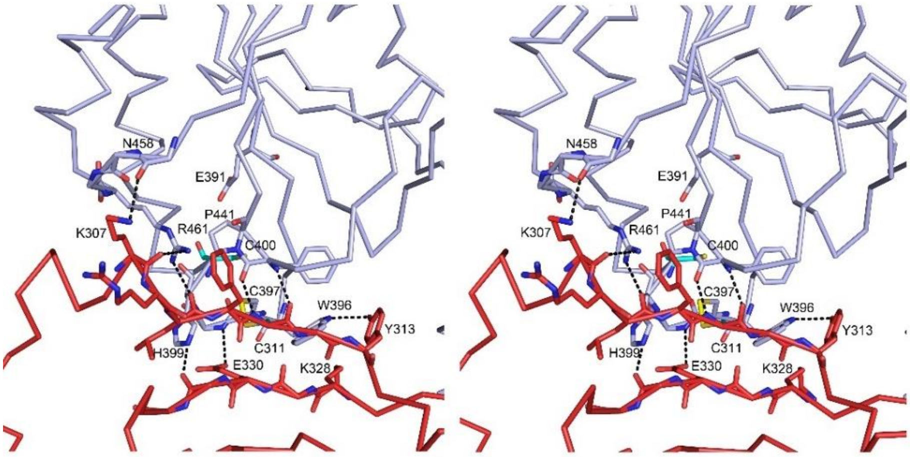

B

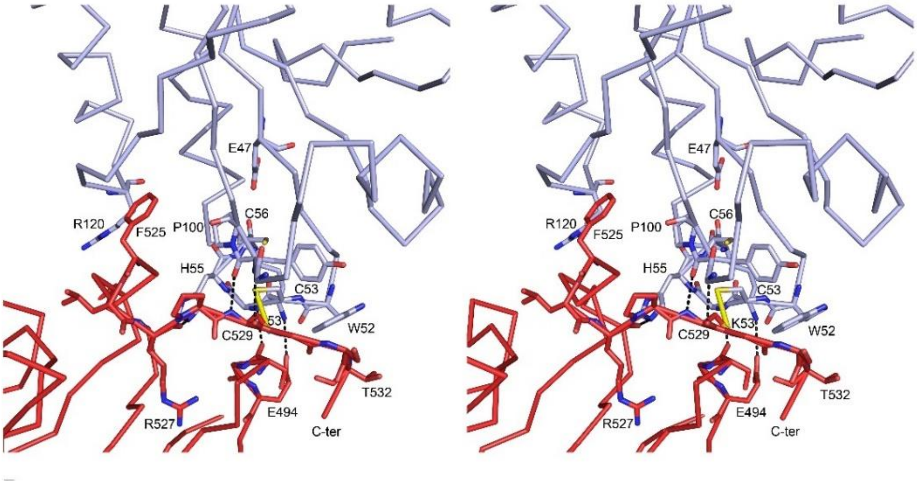

Figure S10. Stereo view of the structural details of the interaction sites of the CAT domain with the a' and a domains of the β/PDI subunit. The CAT domain residues are in red color and the PDI domains are shown in light blue color (in stereo, same view as in **Fig. 4** and **Fig. S8**). Hydrogen bond interactions are visualized by dotted lines. (A) The CGHC-motif of the **a'** domain forms an inter-subunit disulfide bridge with Cys311 of the N-terminal β-strand (β1) of the CAT domain. (B) The CGHC-motif of the **a** domain forms an inter-subunit disulfide bridge with the C-terminal Cys529 of the CAT domain. The interface interactions of Cys311 and Cys529 are similar, but some other interactions at the two interfaces are not the same. For example the interactions of the side chain of His(CGHC) in (A) and (B) are different. Also the interactions of the side chains of the conserved Trp396 and Arg461 of domain **a'** in (A) and Trp52 and Arg120 of domain **a** in (B) are different.

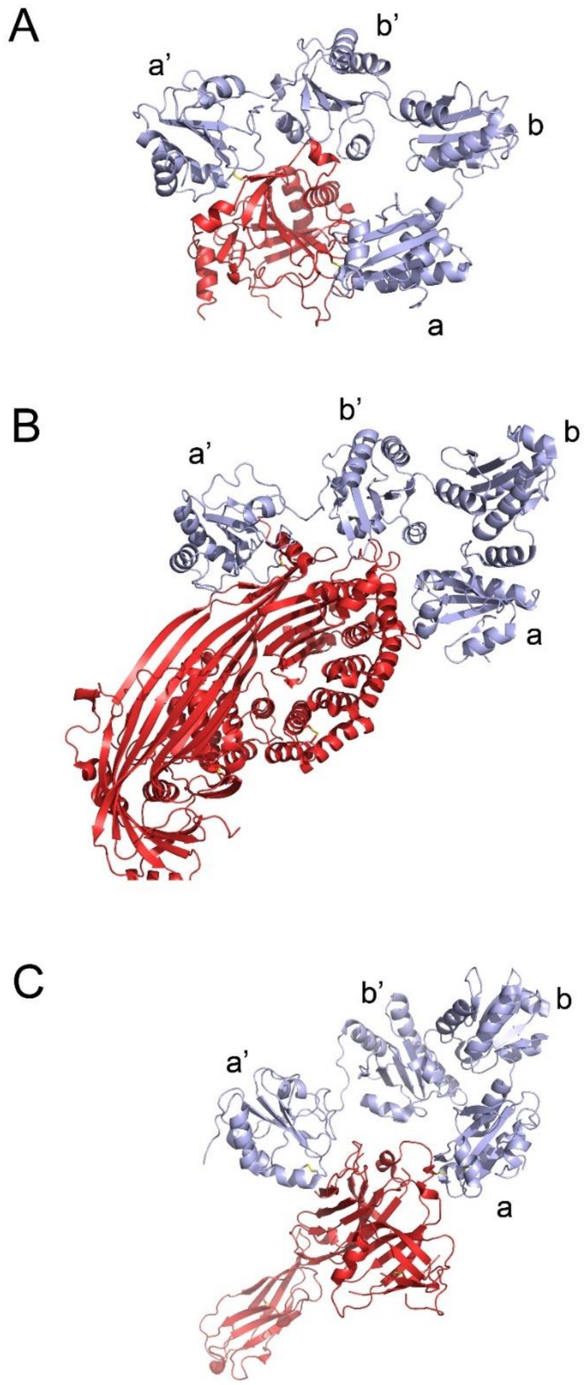

Figure S11. Comparison of the assembly (A) of PDI with the CAT domain of the C-P4H-II-Δ281 complex, (B) of PDI with the α-subunit of MTP and (C) of ERp57 with tapasin. The PDI and ERp57 subunits are colored light blue and the CAT domain, α-subunit of MTP and tapasin are colored red. In the C-P4H-II-Δ281 complex the interactions of the **a'** and **b'** domains of PDI are with the same region of the CAT domain (the N-terminal extension), whereas in the MTP complex (PDB entry 6I7S) the interactions of **a'** and **b'** of PDI are with different domains of the MTP α-subunit. In the ERp57-tapasin complex (PDB entry 3F8U), the **a** domain of ERp57 forms an inter-subunit disulfide bridge with tapasin. In the latter complex the **b'** domain of ERp57 does not interact with tapasin.

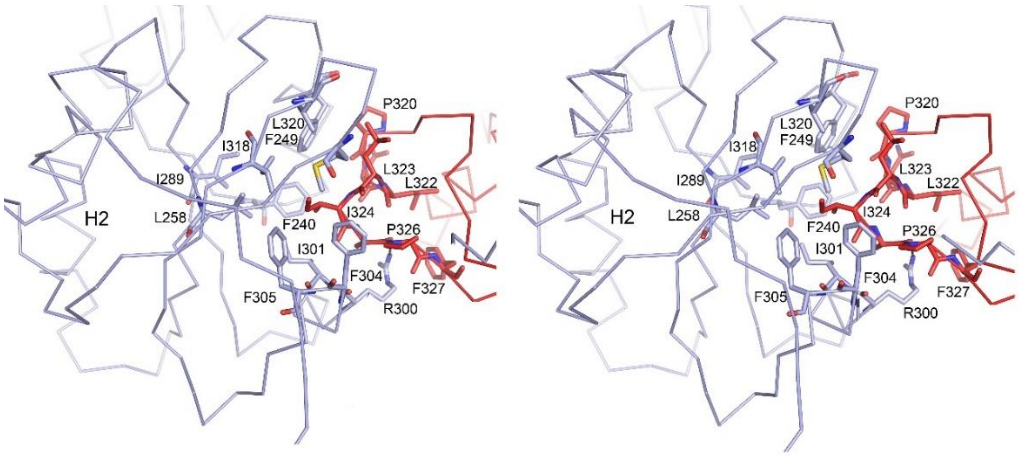

Figure S12. The interactions of the CAT domain in the hydrophobic pocket of the b' domain of the β/PDI subunit. Similar view (in stereo) as in **Fig. S8**. The Cα-trace of the **b'** domain is shown in light blue ribbon and the main chain and side chain atoms of the residues shaping the hydrophobic pocket are also shown and labeled. The CAT domain residues are in red color. The side chain of Ile324, of the α14-helix of the CAT domain, points into the hydrophobic pocket of the **b'** domain. Also visualized is the hydrogen bond interaction of the Arg300 (β/PDI) side chain with the Ala325 main chain oxygen (of the α14-helix of the CAT domain).

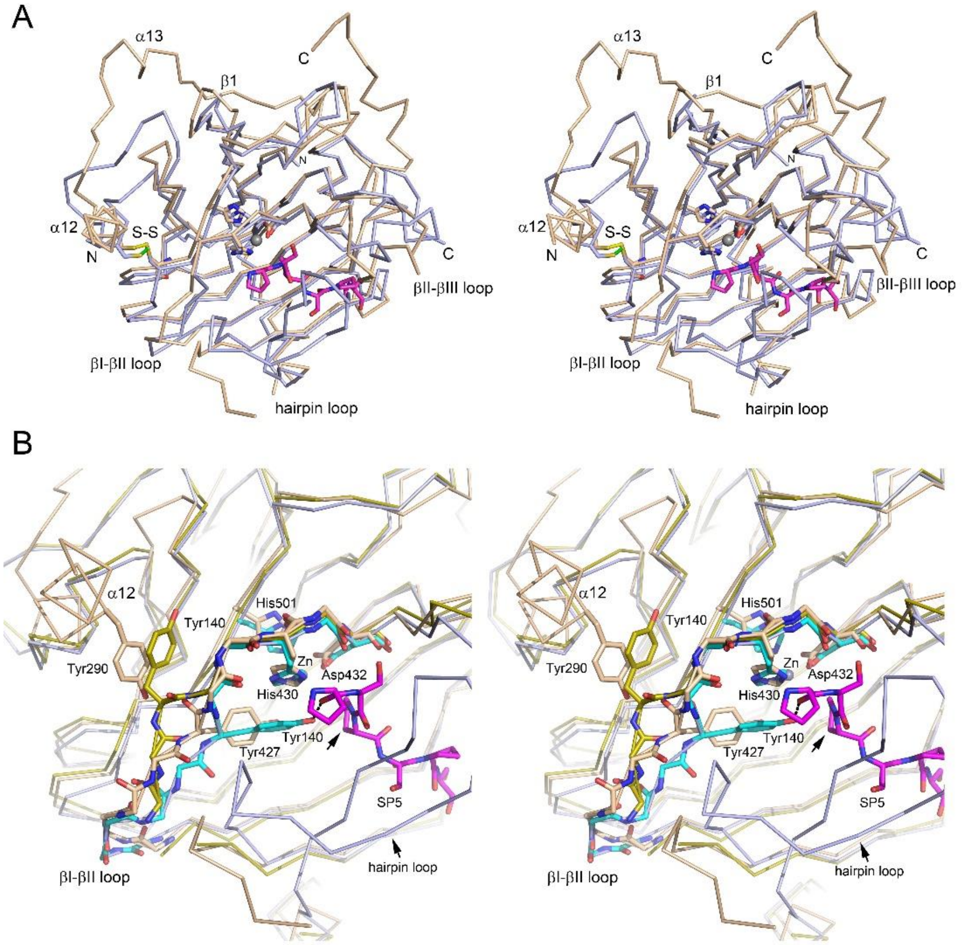

Figure S13. Comparison of the structures of the CAT domains of C-P4H-II-Δ281 and Cr-P4H. (A) Stereo view of the Cα-traces of the α-subunit of the C-P4H-II-Δ281 complex (light brown) superimposed on the Cr-P4H structure (light blue, PDB entry 3GZE, molecule A) in complex with Zn(II) (gray sphere) (the Zn(II) ion has replaced the Fe(II) active site ion, resulting in an incompetent active site) and $(Ser\text{-}Pro)_5$ peptide substrate (magenta sticks). The α12-helix, the α13-helix and β-strand β1 are additional structural elements of C-P4H-II-Δ281, missing in Cr-P4H. Also, the C-terminus (labeled with "C") is more extended in C-P4H-II-Δ281. The loop regions that are important in peptide substrate binding (the hairpin and βII-βIII loop), are labelled. The hairpin loop is disordered in the structure of the C-P4H-II-Δ281 complex and it is in the "closed" conformation in the Cr-P4H peptide complex. The side chains of the three important catalytic amino acids, two histidines and one aspartate, coordinating the bound Zn(II) ion in the 3GZE structure, are shown. Also the intra-subunit disulfide bond (labeled as S-S) of the conserved cysteine of the βVII-strand (Cys504 in the C-P4H-II-Δ281 complex, Cys230 in Cr-P4H) with Cys294 (α12-helix in the N-terminal region of the CAT domain of C-P4H-II-Δ281) and Cys195 (extended βIV-βV loop of Cr-P4H), respectively, is shown. (B) Zoomed in view of panel (A) (in stereo) of the active site of the CAT domain, highlighting the βI-βII loop region (Gly422-Pro429) and the following His-X-Asp motif (residues His430, Asp432, His501), which are shown as sticks, like the side chain of Tyr427. Included is also the superimposed βI-βII loop region as seen (i) in the structure of the Cr-P4H enzyme–substrate complex (PDB entry 3GZE, molecule A, as in panel (A), light blue) and (ii) as seen in the structure of Cr-P4H without bound peptide (molecule B, PDB entry 2JIG, yellow), highlighting the two different conformations of Tyr140. In the structure of the Cr-P4H enzyme substrate complex, the Tyr140 side chain is hydrogen-bonded to the bound $(Ser\text{-}Pro)_5$ peptide substrate as shown with a dashed line. The proline residue to be hydroxylated by the active site is identified with an arrow. Zn identifies the Zn(II) ion binding position in the catalytic site of the Cr-P4H enzyme-substrate complex. In the structure of Cr-P4H without bound peptide the Tyr140 side chain is rotated outwards (yellow). The latter conformation is not possible in the C-P4H-II-Δ281 structure, as its corresponding tyrosine (Tyr427) would clash with Tyr290 of the α12-helix.

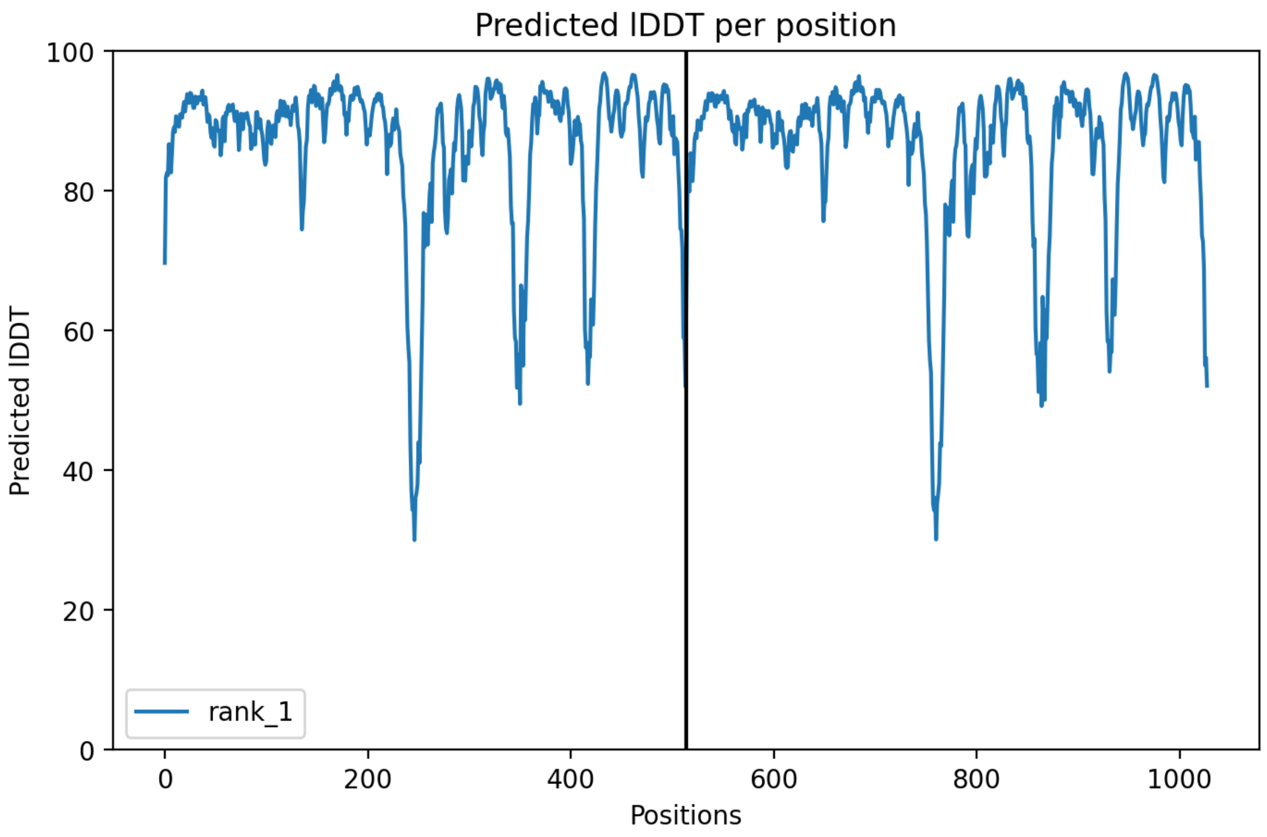

Figure S14. pLDDT plot calculated by AlphaFold2 for the structure prediction of the α$_2$-dimer of the mature C-P4H-II. pLDDT refers to the predicted Local Distance Difference Test values. Values above 90 refer to a reliable prediction. The prediction calculations were done with the sequence of the mature α-subunit of human C-P4H-II. The pLDDT values are plotted on the Y-axis as a function of the residue numbers, which are plotted on the X-axis. The left and right parts of the diagram refer, respectively, to the two α-subunits which together form the α$_2$-dimer. The regions with a low pLDDT score, for both α-subunits, concern the region between the PSB domain and the CAT domain and the regions of the hairpin loop and the βII-βIII loop, as discussed in the text.
