# Supplementary material for: Crystal structure of the collagen prolyl 4-hydroxylase (C-P4H) catalytic domain complexed with PDI: Toward a model of the C-P4H α2β2 tetramer
Source: J Biol Chem. 2022 Oct 18;298(12):102614. doi: 10.1016/j.jbc.2022.102614 (PMC9676403; doi:10.1016/j.jbc.2022.102614)
Supplement: Supplemental table S2 [file mmc2.pdf]

Table S2. The peptides observed in the mass spectrometry mapping experiment with the purified mature C-P4H-II complex, are listed for each of the three experiments as described in Experimental Procedures section

C-P4H-II, native experiment
α-subunit is residues 14-535 (residues 14-20 includes the 6x-His-tag residues)
β-subunit is residues 17-508
α-subunit: sequence coverage 78% (405 / 522 amino acids)
β-subunit: sequence coverage 91% (450 / 492 amino acids)

α-subunit

Experimental mass (Da)	Theoretical mass (Da)	Error (ppm)	From	To	Sequence
1019.5256	1019.5288	-3.14	103	112	LVLQDSAAGF
1222.5616	1222.5652	-2.99	525	535	FLRPCGSTEVD (Cys529, free thiol)
1320.6830	1320.6860	-2.27	56	66	SKIKSWANKME
1323.7221	1323.7194	2.02	398	408	VNRRMQHITGL
1369.6936	1369.6990	-3.94	375	386	TVASYRVSKSSW
1378.5522	1378.5560	-2.78	14	23	MHHHHHHMEF
1382.7937	1382.7994	-4.11	362	374	ARATVRDPKTGVL
1580.8602	1580.8675	-4.59	216	229	AVFQLGDLHRALEL
1960.0355	1960.0418	-3.23	87	102	YKLVKRLNTDWPALED
2033.2397	2033.2473	-3.72	356	374	IAKPKLARATVRDPKTGVL
2039.9294	2039.9377	-4.08	416	433	LQVANYGVGGQYEPHFDF
2189.9372	2189.9477	-4.81	173	190	GRSAYNEGDYYHTVLWME
2221.0938	2221.1015	-3.48	116	135	LSVQRQFFPTDEDEIGAAKA
2263.1145	2263.1161	-0.69	320	338	PQLLIAPFKEEDEWDSPHI
2273.1257	2273.1261	-0.19	434	453	SRNDERDTFKHLGTGNRVAT
2310.2222	2310.2305	-3.58	230	249	TRRLLSLDPSHERAGGNLRY
2343.1446	2343.1536	-3.84	323	341	LIAPFKEEDEWDSPHIVRY
2352.1320	2352.1420	-4.27	117	137	SVQRQFFPTDEDEIGAAKALM
2479.3451	2479.3462	-0.43	33	53	LIYAEKELVQSLKEYILVEEA
2562.2858	2562.2973	-4.47	387	408	LEEDDDPVVARVNRRMQHITGL
2827.4745	2827.4842	-3.44	230	253	TRRLLSLDPSHERAGGNLRYFEQL
2834.4186	2834.4313	-4.48	458	485	MSDVEAGGATVFPDLGAAIWPKKGTAVF
2864.3976	2864.4127	-5.27	117	141	SVQRQFFPTDEDEIGAAKALMRLQD
2983.4898	2983.4927	-0.96	200	225	EEATTTKSQVLDYLSYAVFQLGDLHR
3111.5254	3111.5376	-3.94	456	485	NYMSDVEAGGATVFPDLGAAIWPKKGTAVF
3311.4767	3311.4969	-6.12	323	349	LIAPFKEEDEWDSPHIVRYYDVMSDEE
3770.0188	3770.0401	-5.66	342	374	YDVMSDEEIERIKEIAKPKLARATVRDPKTGVL
4079.9259	4079.9463	-5.00	323	355	LIAPFKEEDEWDSPHIVRYYDVMSDEEIERIKE
4295.0317	4295.0533	-5.04	416	453	LQVANYGVGGQYEPHFDFSRNDERDTFKHLGTGNRVAT
4442.0984	4442.1217	-5.24	416	454	LQVANYGVGGQYEPHFDFSRNDERDTFKHLGTGNRVATF
4555.1820	4555.2058	-5.23	416	455	LQVANYGVGGQYEPHFDFSRNDERDTFKHLGTGNRVATFL
4656.3880	4656.4082	-4.34	375	415	TVASYRVSKSSWLEEDDDPVVARVNRRMQHITGLTVKTAEL
4832.2861	4832.3120	-5.37	416	457	LQVANYGVGGQYEPHFDFSRNDERDTFKHLGTGNRVATFLNY
5299.6083	5299.6394	-5.88	117	163	SVQRQFFPTDEDEIGAAKALMRLQDTYRLDPGTISRGELPGTKYQAM
6095.1628	6095.1830	-3.32	323	374	LIAPFKEEDEWDSPHIVRYYDVMSDEEIERIKEIAKPKLARATVRDPKTGVL

β-subunit

Experimental mass (Da)	Theoretical mass (Da)	Error (ppm)	From	To	Sequence
452.1591	452.1577	2.99	425	428	MDST
528.3253	528.3271	-3.33	419	423	NIVIA
604.3565	604.3584	-3.13	155	160	VAVIGF
645.3310	645.3334	-3.70	198	203	LDKDGV
982.4719	982.4794	-7.65	324	331	MTKYKPES
1021.5158	1021.5193	-3.44	459	467	GERTLDGFK
1098.6244	1098.6298	-4.90	127	135	NWLKKRTGP
1111.5178	1111.5220	-3.74	324	332	MTKYKPESE
1122.4821	1122.4863	-3.75	424	433	KMDSTANEVE
1176.5469	1176.5510	-3.48	80	90	AKVDATEESDL
1198.6668	1198.6710	-3.54	35	45	AEALAAHKYLL
1253.6275	1253.6292	-1.32	383	392	DEKKNVFVEF
1302.7616	1302.7660	-3.42	229	239	IKHNQLPLVIE
1303.5710	1303.5681	2.26	289	299	IFIDSDHTDNQ
1325.7317	1325.7343	-1.95	432	443	VEAVKVHSFPTL
1363.7297	1363.7347	-3.69	194	205	SKYQLDKDGVVL
1372.7821	1372.7867	-3.36	434	445	AVKVHSFPTLKF
1426.7781	1426.7820	-2.75	35	47	AEALAAHKYLLVE
1438.8137	1438.8184	-3.30	276	288	KTAAESFKGKILF
1449.8281	1449.8344	-4.35	228	239	FIKHNQLPLVIE
1495.6810	1495.6865	-3.66	17	29	MDAPEEEDHVLVL
1510.7973	1510.8031	-3.87	193	205	FSKYQLDKDGVVL
1532.6782	1532.6842	-3.90	496	508	EEDDDQKAVKDEL
1558.8135	1558.8203	-4.36	77	90	IRLAKVDATEESDL
1573.8452	1573.8504	-3.30	35	48	AEALAAHKYLLVEF
1586.7557	1586.7617	-3.76	377	389	FEDVAFDEKKNVF
1626.8475	1626.8518	-2.67	91	104	AQQYGVRGYPTIKF
1632.7982	1632.8029	-2.91	419	433	NIVIAKMDSTANEVE
1677.9369	1677.9454	-5.09	226	239	LDFIKHNQLPLVIE
1711.8375	1711.8417	-2.44	161	175	FKDVESDSAKQFLQA
1961.9432	1961.9483	-2.60	289	304	IFIDSDHTDNQRILEF
2031.9884	2031.9961	-3.80	72	90	AEGSEIRLAKVDATEESDL
2095.1614	2095.1677	-3.00	241	259	TEQTAPKIFGGEIKTHILL
2128.0189	2128.0259	-3.28	17	34	MDAPEEEDHVLVLRKSNF
2196.0351	2196.0447	-4.36	446	465	FPASADRTVIDYNGERTLDG
2204.9178	2204.9267	-4.03	490	508	AEEPDMEEDDDQKAVKDEL
2206.1827	2206.1899	-3.26	30	48	RKSNFAEALAAHKYLLVEF
2242.2272	2242.2362	-4.00	240	259	FTEQTAPKIFGGEIKTHILL
2256.9565	2256.9659	-4.16	466	486	FKKFLESGGQDGAGDDDDLED
2400.1611	2400.1710	-4.14	206	225	FKKFDEGRNNFEGEVTKENL
2439.2370	2439.2468	-4.00	356	376	MSQELPEDWDKQPVKVLVGKN
2463.0015	2463.0119	-4.21	488	508	EEAEEPDMEEDDDQKAVKDEL
2471.1991	2471.2081	-3.66	444	465	KFFPASADRTVIDYNGERTLDG
2576.0862	2576.0960	-3.79	487	508	LEEAEEPDMEEDDDQKAVKDEL
2628.1216	2628.1351	-5.13	466	489	FKKFLESGGQDGAGDDDDLEDLEE

2628.2701	2628.2820	-4.54	206	227	FKKFDEGRNNFEGEVTKENLLD
2711.2446	2711.2576	-4.79	105	128	FRNGDTASPKEYTAGREADDIVNW
2785.3960	2785.3923	1.34	80	104	AKVDATEESDLAQQYGVRGYPTIKF
2946.4011	2946.4157	-4.96	49	76	YAPWCGHCKALAPEYAKAAGKLKAEGSE (Cys53-Cys56)
3085.4055	3085.4215	-5.18	393	418	YAPWCGHCKQLAPIWDKLGETYKDHE (Cys397-Cys400)
3093.4678	3093.4841	-5.27	48	76	FYAPWCGHCKALAPEYAKAAGKLKAEGSE (Cys53-Cys56)
3167.6483	3167.6615	-4.18	77	104	IRLAKVDATEESDLAQQYGVRGYPTIKF
3236.7001	3236.7122	-3.75	260	288	FLPKSVSDYDGKLSNFKTAAESFKGKILF
3255.6027	3255.6187	-4.92	176	205	AEAIDDIPFGITSNSDVFSKYQLDKDGVVL
3308.6699	3308.6864	-4.98	17	45	MDAPEEEDHVLVLRKSNFAEALAAHKYLL
3321.5781	3321.5951	-5.13	46	76	VEFYAPWCGHCKALAPEYAKAAGKLKAEGSE (Cys53-Cys56)
3328.6704	3328.6849	-4.37	49	79	YAPWCGHCKALAPEYAKAAGKLKAEGSEIRL (Cys397-Cys400)
3349.7820	3349.7962	-4.25	259	288	LFLPKSVSDYDGKLSNFKTAAESFKGKILF
3460.5851	3460.6009	-4.56	390	418	VEFYAPWCGHCKQLAPIWDKLGETYKDHE (Cys397-Cys400)
3475.7393	3475.7533	-4.03	48	79	FYAPWCGHCKALAPEYAKAAGKLKAEGSEIRL (Cys53-Cys56)
3524.6863	3524.7010	-4.16	393	422	YAPWCGHCKQLAPIWDKLGETYKDHENIVI (Cys397-Cys400)
3536.7839	3536.7974	-3.81	17	47	MDAPEEEDHVLVLRKSNFAEALAAHKYLLVE
3550.8086	3550.8209	-3.45	434	465	AVKVHSFPTLKFFPASADRTVIDYNGERTLDG
3595.7199	3595.7381	-5.06	393	423	YAPWCGHCKQLAPIWDKLGETYKDHENIVIA (Cys397-Cys400)
3683.8481	3683.8658	-4.80	17	48	MDAPEEEDHVLVLRKSNFAEALAAHKYLLVEF
3703.8436	3703.8643	-5.58	46	79	VEFYAPWCGHCKALAPEYAKAAGKLKAEGSEIRL (Cys53-Cys56)
3899.8572	3899.8804	-5.95	390	422	VEFYAPWCGHCKQLAPIWDKLGETYKDHENIVI (Cys397-Cys400)
3970.8954	3970.9175	-5.56	390	423	VEFYAPWCGHCKQLAPIWDKLGETYKDHENIVIA (Cys397-Cys400)
4007.9773	4007.9979	-5.15	356	389	MSQELPEDWDKQPVKVLVGKNFEDVAFDEKKNVF
4040.1106	4040.1299	-4.78	240	275	FTEQTAPKIFGGEIKTHILLFLPKSVSDYDGKLSNF
4060.0863	4060.1058	-4.80	206	239	FKKFDEGRNNFEGEVTKENLLDFIKHNQLPLVIE
4089.0071	4089.0291	-5.37	116	154	YTAGREADDIVNWLKKRTGPAATTLPDGAAAESLVESSE
4207.1513	4207.1742	-5.44	206	240	FKKFDEGRNNFEGEVTKENLLDFIKHNQLPLVIEF
4434.9704	4435.0000	-6.67	446	486	FPASADRTVIDYNGERTLDGFKKFLESGGQDGAGDDDDLED
4502.2356	4502.2555	-4.41	35	76	AEALAAHKYLLVEFYAPWCGHCKALAPEYAKAAGKLKAEGSE (Cys53-Cys56)
4710.1327	4710.1634	-6.51	444	486	KFFPASADRTVIDYNGERTLDGFKKFLESGGQDGAGDDDDLED
4760.3590	4760.3794	-4.28	105	149	FRNGDTASPKEYTAGREADDIVNWLKKRTGPAATTLPDGAAAESL
4806.1415	4806.1693	-5.78	446	489	FPASADRTVIDYNGERTLDGFKKFLESGGQDGAGDDDDLEDLEE
4815.0307	4815.0513	-4.27	466	508	FKKFLESGGQDGAGDDDDLEDLEEAEEPDMEEDDDQKAVKDEL
4949.4111	4949.4499	-7.85	161	205	FKDVESDSAKQFLQAAEAIDDIPFGITSNSDVFSKYQLDKDGVVL
5291.5700	5291.5971	-5.12	105	154	FRNGDTASPKEYTAGREADDIVNWLKKRTGPAATTLPDGAAAESL VESSE
5460.9011	5460.9378	-6.73	240	288	FTEQTAPKIFGGEIKTHILLFLPKSVSDYDGKLSNFKTAAESFKGKIL F
5718.7094	5718.7391	-5.19	435	486	VKVHSFPTLKFFPASADRTVIDYNGERTLDGFKKFLESGGQDGAG DDDDLED
5789.7375	5789.7762	-6.68	434	486	AVKVHSFPTLKFFPASADRTVIDYNGERTLDGFKKFLESGGQDGA GDDDDLED
6043.0625	6043.0652	-0.45	35	90	AEALAAHKYLLVEFYAPWCGHCKALAPEYAKAAGKLKAEGSEIRLA KVDATEESDL (Cys53-Cys56)
6096.0192	6096.0666	-7.78	49	104	YAPWCGHCKALAPEYAKAAGKLKAEGSEIRLAKVDATEESDLAQQ YGVRGYPTIKF (Cys53-Cys56)
6160.9049	6160.9454	-6.58	434	489	AVKVHSFPTLKFFPASADRTVIDYNGERTLDGFKKFLESGGQDGA GDDDDLEDLEE
6243.1246	6243.1350	-1.67	48	104	FYAPWCGHCKALAPEYAKAAGKLKAEGSEIRLAKVDATEESDLAQ QYGVRGYPTIKF (Cys53-Cys56)

C-P4H-II, DTT reduced sample
α-subunit is residues 14-535 (residues 14-20 includes the 6x-His-tag residues)
β-subunit is residues 17-508
α-subunit: 496 / 522 amino acids, sequence coverage 95 %
β-subunit: 492 / 492 amino acids, sequence coverage 100 %

α-subunit

Experimental mass (Da)	Theoretical mass (Da)	Error (ppm)	From	To	Sequence
804.3840	804.3840	-0.05	188	193	WMEQVL
883.4547	883.4552	-0.53	79	86	YLAHPVNA
1019.5285	1019.5288	-0.30	103	112	LVLQDSAAGF
1120.5218	1120.5223	-0.49	24	33	FTSIGHMTDL
1216.6968	1216.6968	-0.03	475	485	AIWPKKGTAVF
1222.5651	1222.5652	-0.07	525	535	FLRPCGSTEVD (Cys529, free thiol)
1378.5565	1378.5560	0.39	14	23	MHHHHHHMEF
1382.7986	1382.7994	-0.59	362	374	ARATVRDPKTGVL
1555.7997	1555.7995	0.11	90	102	VKRLNTDWPALED
1580.8671	1580.8675	-0.24	216	229	AVFQLGDLHRALEL
1597.7681	1597.7685	-0.23	310	322	FCRYHHGNRAPQL (Cys311, free thiol)
1754.8028	1754.8033	-0.30	342	355	YDVMSDEEIERIKE
1896.0947	1896.0952	-0.27	294	309	CRGEGVKLTPRRQKRL (Cys294, free thiol)
1918.9111	1918.9120	-0.45	254	269	LEEEREKTLTNQTEAE
1932.9054	1932.9053	0.06	510	524	CKWVSNKWFHERGQE (Cys510, free thiol)
1960.0419	1960.0418	0.03	87	102	YKLVKRLNTDWPALED
1992.1829	1992.1844	-0.76	359	377	PKLARATVRDPKTGVLTVA
2039.9367	2039.9377	-0.49	416	433	LQVANYGVGGQYEPHFDF
2088.9954	2088.9952	0.09	508	524	VGCKWVSNKWFHERGQE (Cys510, free thiol)
2108.0166	2108.0175	-0.41	117	135	SVQRQFFPTDEDEIGAAKA
2221.1016	2221.1015	0.06	117	136	SVQRQFFPTDEDEIGAAKAL
2267.0652	2267.0654	-0.11	486	504	WYNLLRSGEGDYRTRHAAC (Cys504, free thiol)
2273.1248	2273.1261	-0.56	434	453	SRNDERDTFKHLGTGNRVAT
2310.2302	2310.2305	-0.14	230	249	TRRLLSLDPSHERAGGNLRY
2343.1535	2343.1536	-0.06	323	341	LIAPFKEEDEWDSPHIVRY
2352.1417	2352.1420	-0.11	117	137	SVQRQFFPTDEDEIGAAKALM
2405.2507	2405.2512	-0.20	342	361	YDVMSDEEIERIKEIAKPKL
2436.1661	2436.1656	0.20	250	269	FEQLLEEEREKTLTNQTEAE
2453.2369	2453.2373	-0.18	142	163	TYRLDPGTISRGELPGTKYQAM
2533.2786	2533.2786	0.00	434	455	SRNDERDTFKHLGTGNRVATFL
2535.2207	2535.2215	-0.30	432	453	DFSRNDERDTFKHLGTGNRVAT
2557.3162	2557.3117	1.77	90	112	VKRLNTDWPALEDLVLQDSAAGF
2562.2954	2562.2973	-0.75	387	408	LEEDDDPVVARVNRRMQHITGL
2566.3214	2566.3214	0.00	142	164	TYRLDPGTISRGELPGTKYQAML
2623.2790	2623.2806	-0.62	270	291	LATPEGIYERPVDYLPERDVYE
2632.3491	2632.3497	-0.22	113	136	IANLSVQRQFFPTDEDEIGAAKAL
2710.3121	2710.3126	-0.20	270	292	LATPEGIYERPVDYLPERDVYES
2827.4800	2827.4842	-1.47	230	253	TRRLLSLDPSHERAGGNLRYFEQL
2834.4305	2834.4313	-0.30	458	485	MSDVEAGGATVFPDLGAAIWPKKGTAVF

2864.4088	2864.4127	-1.37	117	141	SVQRQFFPTDEDEIGAAKALMRLQD
2961.5596	2961.5600	-0.15	87	112	YKLVKRLNTDWPALEDLVLQDSAAGF
2965.4569	2965.4604	-1.19	117	142	SVQRQFFPTDEDEIGAAKALMRLQDT
2973.6573	2973.6573	0.01	299	322	VKLTPRRQKRLFCRYHHGNRAPQL (Cys294, free thiol)
3096.5476	3096.5485	-0.29	137	163	MRLQDTYRLDPGTISRGELPGTKYQAM
3111.5377	3111.5376	0.04	456	485	NYMSDVEAGGATVFPDLGAAIWPKKGTAVF
3209.6324	3209.6325	-0.03	137	164	MRLQDTYRLDPGTISRGELPGTKYQAML
3224.6215	3224.6216	-0.04	455	485	LNYMSDVEAGGATVFPDLGAAIWPKKGTAVF
3293.5489	3293.5499	-0.29	508	535	VGCKWVSNKWFHERGQEFLRPCGSTEVD (Cys510, Cys529, free thiols)
3372.8368	3372.8439	-2.11	295	322	RGEGVKLTPRRQKRLFCRYHHGNRAPQL (Cys311, free thiol)
3475.8515	3475.8531	-0.46	294	322	CRGEGVKLTPRRQKRLFCRYHHGNRAPQL (Cys294, Cys311, free thiols)
3600.9013	3600.8947	1.82	397	428	RVNRRMQHITGLTVKTAELLQVANYGVGGQYE
3675.9685	3675.9692	-0.20	292	322	SLCRGEGVKLTPRRQKRLFCRYHHGNRAPQL (Cys294, Cys311, free thiols)
3805.0105	3805.0118	-0.35	291	322	ESLCRGEGVKLTPRRQKRLFCRYHHGNRAPQL (Cys294, Cys311, free thiols)
3873.0850	3873.0874	-0.62	216	249	AVFQLGDLHRALELTRRLLSLDPSHERAGGNLRY
4079.9454	4079.9463	-0.22	323	355	LIAPFKEEDEWDSPHIVRYYDVMSDEEIERIKE
4295.0526	4295.0533	-0.17	416	453	LQVANYGVGGQYEPHFDFSRNDERDTFKHLGTGNRVAT
4442.1212	4442.1217	-0.11	416	454	LQVANYGVGGQYEPHFDFSRNDERDTFKHLGTGNRVATF
4555.2029	4555.2058	-0.64	416	455	LQVANYGVGGQYEPHFDFSRNDERDTFKHLGTGNRVATFL
5299.6369	5299.6394	-0.47	117	163	SVQRQFFPTDEDEIGAAKALMRLQDTYRLDPGTISRGELPGTKYQAM
5412.7198	5412.7235	-0.69	117	164	SVQRQFFPTDEDEIGAAKALMRLQDTYRLDPGTISRGELPGTKYQAML
6021.1930	6021.1971	-0.68	362	415	ARATVRDPKTGVLTVASYRVSKSSWLEEDDDPVVARVNRRMQHITGLTVKTAEL
6114.1514	6114.1585	-1.16	24	78	FTSIGHMTDLIYAEKELVQSLKEYILVEEAKLSKIKSWANKMEALTSKSAADAEG
6281.2340	6281.2392	-0.83	270	322	LATPEGIYERPVDYLPERDVYESLCRGEGVKLTPRRQKRLFCRYHHGNRAPQL (Cys294, Cys311, free thiols)
6314.2822	6314.2836	-0.22	194	249	KQLDAGEEATTTKSQVLDYLSYAVFQLGDLHRALELTRRLLSLDPSHERAGGNLRY
6979.5986	6979.6032	-0.65	24	86	FTSIGHMTDLIYAEKELVQSLKEYILVEEAKLSKIKSWANKMEALTSKSAADAEGYLAHPVNA

β-subunit

Experimental mass (Da)	Theoretical mass (Da)	Error (ppm)	From	To	Sequence
1026.5858	1026.5862	-0.44	435	443	VKVHSFPTL
1097.6232	1097.6233	-0.14	434	443	AVKVHSFPTL
1122.4862	1122.4863	-0.12	424	433	KMDSTANEVE
1192.7234	1192.7220	1.19	283	292	KGKILFIFID
1193.5232	1193.5234	-0.17	423	433	AKMDSTANEVE
1198.6704	1198.6710	-0.50	35	45	AEALAAHKYLL
1301.7494	1301.7496	-0.17	435	445	VKVHSFPTLKF
1363.7350	1363.7347	0.20	194	205	SKYQLDKDGVVL
1372.7868	1372.7867	0.04	434	445	AVKVHSFPTLKF
1426.7822	1426.7820	0.12	35	47	AEALAAHKYLLVE
1436.8421	1436.8391	2.12	250	262	GGEIKTHILLFLP
1438.8179	1438.8184	-0.36	276	288	KTAAESFKGKILF
1488.8119	1488.8123	-0.24	305	317	FGLKKEECPAVRL (Cys312, free thiol)
1495.6865	1495.6865	-0.01	17	29	MDAPEEEDHVLVL
1508.6896	1508.6896	0.02	116	128	YTAGREADDIVNW

1510.8028	1510.8031	-0.17	193	205	SKYQLDKDGVVLF
1573.8507	1573.8504	0.18	35	48	AEALAAHKYLLVEF
1586.7615	1586.7617	-0.16	377	389	FEDVAFDEKKNVF
1626.8525	1626.8518	0.44	91	104	AQQYGVRGYPTIKF
1632.8024	1632.8029	-0.30	419	433	NIVIAKMDSTANEVE
1711.8414	1711.8417	-0.20	161	175	FKDVESDSAKQFLQA
1723.9340	1723.9345	-0.27	342	355	FCHRFLEGKIKPHL (Cys343, free thiol)
1928.9875	1928.9884	-0.49	259	275	LFLPKSVSDYDGKLSNF
1961.9457	1961.9483	-1.32	289	304	IFIDSDHTDNQRILEF
1982.0830	1982.0837	-0.37	241	258	TEQTAPKIFGGEIKTHIL
2128.0261	2128.0259	0.11	17	34	MDAPEEEDHVLVLRKSNF
2129.1525	2129.1521	0.19	240	258	FTEQTAPKIFGGEIKTHIL
2196.0441	2196.0447	-0.27	446	465	FPASADRTVIDYNGERTLDG
2202.0985	2202.0991	-0.26	424	443	KMDSTANEVEAVKVHSFPTL
2204.9264	2204.9267	-0.15	490	508	AEEPDMEEDDDQKAVKDEL
2206.1896	2206.1899	-0.13	30	48	RKSNFAEALAAHKYLLVEF
2242.2357	2242.2362	-0.21	240	259	FTEQTAPKIFGGEIKTHILL
2439.2468	2439.2468	0.01	356	376	MSQELPEDWDKQPVKVLVGKN
2471.2075	2471.2081	-0.26	444	465	KFFPASADRTVIDYNGERTLDG
2477.2620	2477.2625	-0.21	424	445	KMDSTANEVEAVKVHSFPTLKF
2524.3372	2524.3373	-0.06	335	355	TAERITEFCHRFLEGKIKPHL (Cys343, free thiol)
2548.2990	2548.2996	-0.22	423	445	AKMDSTANEVEAVKVHSFPTLKF
2785.3919	2785.3923	-0.15	80	104	AKVDATEESDLAQQYGVRGYPTIKF
3087.4334	3087.4371	-1.19	393	418	YAPWCGHCKQLAPIWDKLGETYKDHE (Cys397, Cys400, free thiols)
3167.6612	3167.6615	-0.11	77	104	IRLAKVDATEESDLAQQYGVRGYPTIKF
3236.7122	3236.7122	0.01	260	288	FLPKSVSDYDGKLSNFKTAAESFKGKILF
3255.6177	3255.6187	-0.30	176	205	AEAIDDIPFGITSNSDVFSKYQLDKDGVVL
3308.6856	3308.6864	-0.25	17	45	MDAPEEEDHVLVLRKSNFAEALAAHKYLL
3330.7003	3330.7006	-0.10	49	79	YAPWCGHCKALAPEYAKAAGKLKAEGSEIRL (Cys53, Cys56, free thiols)
3349.7951	3349.7962	-0.34	259	288	LFLPKSVSDYDGKLSNFKTAAESFKGKILF
3432.7492	3432.7500	-0.25	289	317	IFIDSDHTDNQRILEFFGLKKEECPAVRL (Cys312, free thiol)
3456.6549	3456.6573	-0.68	161	192	FKDVESDSAKQFLQAAEAIDDIPFGITSNSDV
3462.6149	3462.6166	-0.50	390	418	VEFYAPWCGHCKQLAPIWDKLGETYKDHE (Cys397, Cys400, free thiols)
3526.7160	3526.7166	-0.16	393	422	YAPWCGHCKQLAPIWDKLGETYKDHENIVI (Cys397, Cys400, free thiols)
3536.7965	3536.7974	-0.24	17	47	MDAPEEEDHVLVLRKSNFAEALAAHKYLLVE
3597.7507	3597.7537	-0.84	393	423	YAPWCGHCKQLAPIWDKLGETYKDHENIVIA (Cys397, Cys400, free thiols)
3683.8644	3683.8658	-0.37	17	48	MDAPEEEDHVLVLRKSNFAEALAAHKYLLVEF
3901.8885	3901.8960	-1.92	390	422	VEFYAPWCGHCKQLAPIWDKLGETYKDHENIVI (Cys397, Cys400, free thiols)
3972.9311	3972.9331	-0.51	390	423	VEFYAPWCGHCKQLAPIWDKLGETYKDHENIVIA (Cys397, Cys400, free thiols)
4007.9966	4007.9979	-0.33	356	389	MSQELPEDWDKQPVKVLVGKNFEDVAFDEKKNVF
4060.1059	4060.1058	0.01	206	239	FKKFDEGRNNFEGEVTKENLLDFIKHNQLPLVIE
4089.0271	4089.0291	-0.48	116	154	YTAGREADDIVNWLKKRTGPAATTLPDGAAAESLVESSE
4207.1723	4207.1742	-0.44	206	240	FKKFDEGRNNFEGEVTKENLLDFIKHNQLPLVIEF
4247.1037	4247.1031	0.14	321	355	EEEMTKYKPESEELTAERITEFCHRFLEGKIKPHL (Cys343, free thiol)
4383.1762	4383.1773	-0.25	356	392	MSQELPEDWDKQPVKVLVGKNFEDVAFDEKKNVFVEF

4434.9966	4435.0000	-0.77	446	486	FPASADRTVIDYNGERTLDGFKKFLESGGQDGAGDDDDLED
4504.2703	4504.2712	-0.20	35	76	AEALAAHKYLLVEFYAPWCGHCKALAPEYAKAAGKLKAEGSE
4574.3168	4574.3189	-0.46	318	355	ITLEEEMTKYKPESEELTAERITEFCHRFLEGKIKPHL (Cys343, free thiol)
4675.3797	4675.3770	0.58	116	160	YTAGREADDIVNWLKKRTGPAATTLPDGAAAESLVESSEVAVIGF
4710.1623	4710.1634	-0.23	444	486	KFFPASADRTVIDYNGERTLDGFKKFLESGGQDGAGDDDDLED
4760.3794	4760.3794	0.01	105	149	FRNGDTASPKEYTAGREADDIVNWLKKRTGPAATTLPDGAAAESL
4806.1669	4806.1693	-0.50	446	489	FPASADRTVIDYNGERTLDGFKKFLESGGQDGAGDDDDLEDLEE
4815.0523	4815.0513	0.20	466	508	FKKFLESGGQDGAGDDDDLEDLEEAEEPDMEEDDDQKAVKDEL
4949.4457	4949.4499	-0.86	161	205	FKDVESDSAKQFLQAAEAIDDIPFGITSNSDVFSKYQLDKDGVVL
5081.3237	5081.3327	-1.78	444	489	KFFPASADRTVIDYNGERTLDGFKKFLESGGQDGAGDDDDLEDLE E
5291.5969	5291.5971	-0.04	105	154	FRNGDTASPKEYTAGREADDIVNWLKKRTGPAATTLPDGAAAESL VESSE
5877.9385	5877.9450	-1.10	105	160	FRNGDTASPKEYTAGREADDIVNWLKKRTGPAATTLPDGAAAESL VESSEVAVIGF
6098.0728	6098.0823	-1.56	49	104	YAPWCGHCKALAPEYAKAAGKLKAEGSEIRLAKVDATEESDLAQQ YGVRGYPTIKF (Cys53, Cys60, free thiols)
6171.2446	6171.2474	-0.45	206	258	FKKFDEGRNNFEGEVTKENLLDFIKHNQLPLVIEFTEQTAPKIFGGE IKTHIL
6245.1549	6245.1507	0.67	48	104	FYAPWCGHCKALAPEYAKAAGKLKAEGSEIRLAKVDATEESDLAQ QYGVRGYPTIKF (Cys53, Cys60, free thiols)
6614.2821	6614.2865	-0.67	17	76	MDAPEEEDHVLVLRKSNFAEALAAHKYLLVEFYAPWCGHCKALAP EYAKAAGKLKAEGSE (Cys53, Cys60, free thiols)
6996.5555	6996.5558	-0.04	17	79	MDAPEEEDHVLVLRKSNFAEALAAHKYLLVEFYAPWCGHCKALAP EYAKAAGKLKAEGSEIRL (Cys53, Cys60, free thiols)
7268.2538	7268.2488	0.68	444	508	KFFPASADRTVIDYNGERTLDGFKKFLESGGQDGAGDDDDLEDLE EAEEPDMEEDDDQKAVKDEL
7653.9239	7653.9222	0.23	35	104	AEALAAHKYLLVEFYAPWCGHCKALAPEYAKAAGKLKAEGSEIRLA KVDATEESDLAQQYGVRGYPTIKF (Cys53, Cys60, free thiols)
9763.9393	9763.9375	0.18	17	104	MDAPEEEDHVLVLRKSNFAEALAAHKYLLVEFYAPWCGHCKALAP EYAKAAGKLKAEGSEIRLAKVDATEESDLAQQYGVRGYPTIKF (Cys53, Cys60, free thiols)

C-P4H-II, NEM treated sample
α-subunit is residues 14-535 (residues 14-20 includes the 6x-His-tag residues)
β-subunit is residues 17-508
α-subunit: 469 / 522 amino acids, sequence coverage 90 %
β-subunit: 492 / 492 amino acids, sequence coverage 100 %

α-subunit

Experimental mass (Da)	Theoretical mass (Da)	Error (ppm)	From	To	Sequence
535.2641	535.2642	-0.11	250	253	FEQL
594.2801	594.2802	-0.25	486	489	WYNL
883.4549	883.4552	-0.36	79	86	YLAHPVNA
1019.5288	1019.5288	-0.02	103	112	LVLQDSAAGF
1120.5223	1120.5223	0.01	24	33	FTSIGHMTDL
1216.6969	1216.6968	0.04	475	485	AIWPKKGTAVF
1222.5653	1222.5652	0.06	525	535	FLRPCGSTEVD (Cys529, free thiol)
1347.6136	1347.6129	0.50	525	535	FLRPCGSTEVD + N-ethylmaleimide (Cys529+NEM)
1378.5563	1378.5560	0.21	14	23	MHHHHHHMEF
1382.7994	1382.7994	0.03	362	374	ARATVRDPKTGVL
1430.7775	1430.7769	0.41	103	116	LVLQDSAAGFIANL
1580.8675	1580.8675	0.03	216	229	AVFQLGDLHRALEL
1669.9190	1669.9192	-0.11	470	485	PDLGAAIWPKKGTAVF
1754.8032	1754.8033	-0.05	342	355	YDVMSDEEIERIKE
1777.8419	1777.8424	-0.30	416	431	LQVANYGVGGQYEPHF
1918.9116	1918.9120	-0.22	254	269	LEEEREKTLTNQTEAE
1960.0425	1960.0418	0.34	87	102	YKLVKRLNTDWPALED
2033.2479	2033.2473	0.30	356	374	IAKPKLARATVRDPKTGVL
2039.9377	2039.9377	0.00	416	433	LQVANYGVGGQYEPHFDF
2108.0175	2108.0175	0.02	117	135	SVQRQFFPTDEDEIGAAKA
2214.0432	2214.0429	0.14	508	524	VGCKWVSNKWFHERGQE + N-ethylmaleimide (Cys510+NEM)
2221.1019	2221.1015	0.16	117	136	SVQRQFFPTDEDEIGAAKAL
2310.2309	2310.2305	0.15	230	249	TRRLLSLDPSHERAGGNLRY
2343.1537	2343.1536	0.03	323	341	LIAPFKEEDEWDSPHIVRY
2352.1422	2352.1420	0.07	117	137	SVQRQFFPTDEDEIGAAKALM
2420.1938	2420.1945	-0.29	434	454	SRNDERDTFKHLGTGNRVATF
2436.1648	2436.1656	-0.32	250	269	FEQLLEEEREKTLTNQTEAE
2453.2375	2453.2373	0.09	142	163	TYRLDPGTISRGELPGTKYQAM
2557.3181	2557.3177	0.16	90	112	VKRLNTDWPALEDLVLQDSAAGF
2562.2962	2562.2973	-0.44	387	408	LEEDDDPVVARVNRRMQHITGL
2623.2799	2623.2806	-0.26	270	291	LATPEGIYERPVDYLPERDVYE
2632.3499	2632.3497	0.09	113	136	IANLSVQRQFFPTDEDEIGAAKAL
2827.4856	2827.4842	0.48	230	253	TRRLLSLDPSHERAGGNLRYFEQL
2834.4308	2834.4313	-0.18	458	485	MSDVEAGGATVFPDLGAAIWPKKGTAVF
2836.2085	2836.2109	-0.84	164	187	LSVDDCFGMGRSAYNEGDYYHTVL + N-ethylmaleimide (Cys169+NEM)
2864.4113	2864.4127	-0.48	117	141	SVQRQFFPTDEDEIGAAKALMRLQD
2961.5598	2961.5600	-0.06	87	112	YKLVKRLNTDWPALEDLVLQDSAAGF
2965.4568	2965.4604	-1.20	117	142	SVQRQFFPTDEDEIGAAKALMRLQDT
3096.5473	3096.5485	-0.39	137	163	MRLQDTYRLDPGTISRGELPGTKYQAM

3098.7050	3098.7050	-0.01	299	322	VKLTPRRQKRLFCRYHHGNRAPQL + N-ethylmaleimide (Cys311+NEM)
3111.5374	3111.5376	-0.06	456	485	NYMSDVEAGGATVFPDLGAAIWPKKGTAVF
3209.6321	3209.6325	-0.12	136	163	LMRLQDTYRLDPGTISRGELPGTKYQAM
3224.6219	3224.6216	0.10	455	485	LNYMSDVEAGGATVFPDLGAAIWPKKGTAVF
3371.6883	3371.6900	-0.52	454	485	FLNYMSDVEAGGATVFPDLGAAIWPKKGTAVF
3372.8073	3372.8082	-0.28	87	116	YKLVKRLNTDWPALEDLVLQDSAAGFIANL
3392.7366	3392.7372	-0.19	380	408	RVSKSSWLEEDDDPVVARVNRRMQHITGL
3418.5966	3418.5976	-0.30	508	535	VGCKWVSNKWFHERGQEFLRPCGSTEVD + N-ethylmaleimide (Cys510+NEM, Cys529 free thiol)
3873.0854	3873.0874	-0.52	216	249	AVFQLGDLHRALELTRRLLSLDPSHERAGGNLRY
3913.9842	3913.9857	-0.39	375	408	TVASYRVSKSSWLEEDDDPVVARVNRRMQHITGL
4079.9458	4079.9463	-0.12	323	355	LIAPFKEEDEWDSPHIVRYYDVMSDEEIERIKE
4135.1593	4135.1597	-0.09	380	415	RVSKSSWLEEDDDPVVARVNRRMQHITGLTVKTAEL
4295.0521	4295.0533	-0.27	416	453	LQVANYGVGGQYEPHFDFSRNDERDTFKHLGTGNRVAT
4442.1218	4442.1217	0.03	416	454	LQVANYGVGGQYEPHFDFSRNDERDTFKHLGTGNRVATF
4555.2032	4555.2058	-0.58	416	455	LQVANYGVGGQYEPHFDFSRNDERDTFKHLGTGNRVATFL
4656.4087	4656.4082	0.12	375	415	TVASYRVSKSSWLEEDDDPVVARVNRRMQHITGLTVKTAEL
4730.3932	4730.3942	-0.21	323	361	LIAPFKEEDEWDSPHIVRYYDVMSDEEIERIKEIAKPKL
4832.3072	4832.3120	-1.00	416	457	LQVANYGVGGQYEPHFDFSRNDERDTFKHLGTGNRVATFLNY
5299.6366	5299.6394	-0.54	117	163	SVQRQFFPTDEDEIGAAKALMRLQDTYRLDPGTISRGELPGTKYQAM
5412.7174	5412.7235	-1.13	117	164	SVQRQFFPTDEDEIGAAKALMRLQDTYRLDPGTISRGELPGTKYQAML
5929.2164	5929.2225	-1.03	356	408	IAKPKLARATVRDPKTGVLTVASYRVSKSSWLEEDDDPVVARVNRRMQHITGL
6021.1950	6021.1971	-0.35	362	415	ARATVRDPKTGVLTVASYRVSKSSWLEEDDDPVVARVNRRMQHITGLTVKTAEL
6979.5974	6979.6032	-0.83	24	86	FTSIGHMTDLIYAEKELVQSLKEYILVEEAKLSKIKSWANKMEALTSKSAADAEGYLAHPVNA
7666.0076	7666.0152	-1.00	342	408	YDVMSDEEIERIKEIAKPKLARATVRDPKTGVLTVASYRVSKSSWLEEDDDPVVARVNRRMQHITGL
9991.1601	9991.1582	0.19	323	408	LIAPFKEEDEWDSPHIVRYYDVMSDEEIERIKEIAKPKLARATVRDPKTGVLTVASYRVSKSSWLEEDDDPVVARVNRRMQHITGL

β-subunit

Experimental mass (Da)	Theoretical mass (Da)	Error (ppm)	From	To	Sequence
400.2798	400.2798	-0.07	77	80	IRL
726.3225	726.3225	-0.07	377	382	FEDVAF
1026.5862	1026.5862	0.03	435	443	VKVHSFPTL
1097.6242	1097.6233	0.83	434	443	AVKVHSFPTL
1122.4861	1122.4863	-0.14	424	433	KMDSTANEVE
1176.5509	1176.5510	-0.05	80	90	AKVDATEESDL
1192.7235	1192.7220	1.27	283	292	KGKILFIFID
1193.5235	1193.5234	0.05	423	433	AKMDSTANEVE
1198.6707	1198.6710	-0.29	35	45	AEALAAHKYLL
1220.5785	1220.5786	-0.11	105	115	FRNGDTASPKE
1301.7498	1301.7496	0.12	435	445	VKVHSFPTLKF
1363.7352	1363.7347	0.34	194	205	SKYQLDKDGVVL
1372.7869	1372.7867	0.17	434	445	AVKVHSFPTLKF
1438.8183	1438.8184	-0.08	276	288	KTAAESFKGKILF
1495.6871	1495.6865	0.37	17	29	MDAPEEEDHVLVL
1510.8030	1510.8031	-0.07	193	205	FSKYQLDKDGVVL
1573.8509	1573.8504	0.29	35	48	AEALAAHKYLLVEF

1586.7618	1586.7617	0.08	377	389	FEDVAFDEKKNVF
1613.8604	1613.8600	0.23	305	317	FGLKKEECPAVRL + N-ethylmaleimide (Cys312+NEM)
1626.8536	1626.8518	1.08	91	104	AQQYGVRGYPTIKF
1632.8032	1632.8029	0.16	419	433	NIVIAKMDSTANEVE
1711.8421	1711.8417	0.21	161	175	FKDVESDSAKQFLQA
1722.8164	1722.8173	-0.53	451	465	DRTVIDYNGERTLDG
1848.9819	1848.9819	-0.01	342	355	FCHRFLEGKIKPHL + N-ethylmaleimide (Cys343+NEM)
1928.9886	1928.9884	0.11	259	275	LFLPKSVSDYDGKLSNF
1961.9482	1961.9483	-0.04	289	304	IFIDSDHTDNQRILEF
1982.0835	1982.0837	-0.10	241	258	TEQTAPKIFGGEIKTHIL
2031.9962	2031.9961	0.07	72	90	AEGSEIRLAKVDATEESDL
2128.0265	2128.0259	0.27	17	34	MDAPEEEDHVLVLRKSNF
2129.1528	2129.1521	0.33	240	258	FTEQTAPKIFGGEIKTHIL
2144.0210	2144.0223	-0.63	44	62	LLVEFYAPWCGHCKALAPE (Cys53-Cys56)
2196.0447	2196.0447	-0.01	446	465	FPASADRTVIDYNGERTLDG
2202.0995	2202.0991	0.16	424	443	KMDSTANEVEAVKVHSFPTL
2204.9270	2204.9267	0.12	490	508	AEEPDMEEDDDQKAVKDEL
2206.1904	2206.1899	0.21	30	48	RKSNFAEALAAHKYLLVEF
2242.2360	2242.2362	-0.09	241	260	TEQTAPKIFGGEIKTHILLF
2273.1361	2273.1362	-0.07	423	443	AKMDSTANEVEAVKVHSFPTL
2439.2469	2439.2468	0.05	356	376	MSQELPEDWDKQPVKVLVGKN
2471.2078	2471.2081	-0.14	444	465	KFFPASADRTVIDYNGERTLDG
2477.2623	2477.2625	-0.08	424	445	KMDSTANEVEAVKVHSFPTLKF
2548.2995	2548.2996	-0.05	423	445	AKMDSTANEVEAVKVHSFPTLKF
2576.0959	2576.0960	-0.03	487	508	LEEAEEPDMEEDDDQKAVKDEL
2628.2820	2628.2820	-0.02	206	227	FKKFDEGRNNFEGEVTKENLLD
2711.2562	2711.2576	-0.53	105	128	FRNGDTASPKEYTAGREADDIVNW
2712.4155	2712.4157	-0.08	419	443	NIVIAKMDSTANEVEAVKVHSFPTL
2785.3920	2785.3923	-0.10	80	104	AKVDATEESDLAQQYGVRGYPTIKF
2946.4148	2946.4157	-0.30	49	76	YAPWCGHCKALAPEYAKAAGKLKAEGSE (Cys53-Cys56)
2982.5000	2982.5022	-0.75	294	317	DHTDNQRILEFFGLKKEECPAVRL + N-ethylmaleimide (Cys312+NEM)
3085.4209	3085.4215	-0.20	393	418	YAPWCGHCKQLAPIWDKLGETYKDHE (Cys397-Cys400)
3147.5573	3147.5587	-0.46	356	382	MSQELPEDWDKQPVKVLVGKNFEDVAF
3167.6612	3167.6615	-0.11	77	104	IRLAKVDATEESDLAQQYGVRGYPTIKF
3236.7122	3236.7122	0.00	260	288	FLPKSVSDYDGKLSNFKTAAESFKGKILF
3255.6183	3255.6187	-0.12	176	205	AEAIDDIPFGITSNSDVFSKYQLDKDGVVL
3308.6856	3308.6864	-0.25	17	45	MDAPEEEDHVLVLRKSNFAEALAAHKYLL
3328.6837	3328.6849	-0.37	49	79	YAPWCGHCKALAPEYAKAAGKLKAEGSEIRL (Cys53-Cys56)
3349.7951	3349.7962	-0.33	259	288	LFLPKSVSDYDGKLSNFKTAAESFKGKILF
3460.6005	3460.6009	-0.12	390	418	VEFYAPWCGHCKQLAPIWDKLGETYKDHE (Cys397-Cys400)
3475.7526	3475.7533	-0.22	48	79	FYAPWCGHCKALAPEYAKAAGKLKAEGSEIRL (Cys53-Cys56)
3524.7012	3524.7010	0.05	393	422	YAPWCGHCKQLAPIWDKLGETYKDHENIVI (Cys397-Cys400)
3536.7964	3536.7974	-0.28	17	47	MDAPEEEDHVLVLRKSNFAEALAAHKYLLVE
3595.7370	3595.7381	-0.30	393	423	YAPWCGHCKQLAPIWDKLGETYKDHENIVIA (Cys397-Cys400)
3683.8645	3683.8658	-0.37	17	48	MDAPEEEDHVLVLRKSNFAEALAAHKYLLVEF
3703.8636	3703.8643	-0.19	46	79	VEFYAPWCGHCKALAPEYAKAAGKLKAEGSEIRL (Cys53-Cys56)
3899.8783	3899.8804	-0.55	390	422	VEFYAPWCGHCKQLAPIWDKLGETYKDHENIVI (Cys397-Cys400)

3970.9150	3970.9175	-0.62	390	423	VEFYAPWCGHCKQLAPIWDKLGETYKDHENIVIA (Cys397-Cys400)
4007.9963	4007.9979	-0.39	356	389	MSQELPEDWDKQPVKVLVGKNFEDVAFDEKKNVF
4060.1059	4060.1058	0.01	206	239	FKKFDEGRNNFEGEVTKENLLDFIKHNQLPLVIE
4089.0267	4089.0291	-0.58	116	154	YTAGREADDIVNWLKKRTGPAATTLPDGAAAESLVESSE
4207.1725	4207.1742	-0.40	206	240	FKKFDEGRNNFEGEVTKENLLDFIKHNQLPLVIEF
4383.1767	4383.1773	-0.13	356	392	MSQELPEDWDKQPVKVLVGKNFEDVAFDEKKNVFVEF
4502.2551	4502.2555	-0.10	35	76	AEALAAHKYLLVEFYAPWCGHCKALAPEYAKAAGKLKAEGSE (Cys53-Cys56)
4574.3168	4574.3189	-0.45	318	355	ITLEEEMTKYKPESEELTAERITEFCHRFLEGKIKPHL + N-ethylmaleimide (Cys343+NEM)
4675.3779	4675.3770	0.20	116	160	YTAGREADDIVNWLKKRTGPAATTLPDGAAAESLVESSEVAVIGF
4700.2074	4700.2138	-1.36	393	433	YAPWCGHCKQLAPIWDKLGETYKDHENIVIAKMDSTANEVE (Cys397-Cys400)
4710.1599	4710.1634	-0.74	444	486	KFFPASADRTVIDYNGERTLDGFKKFLESGGQDGAGDDDDLED
4760.3813	4760.3794	0.40	105	149	FRNGDTASPKEYTAGREADDIVNWLKKRTGPAATTLPDGAAAESL
4806.1701	4806.1693	0.16	446	489	FPASADRTVIDYNGERTLDGFKKFLESGGQDGAGDDDDLEDLEE
4884.5261	4884.5248	0.27	35	79	AEALAAHKYLLVEFYAPWCGHCKALAPEYAKAAGKLKAEGSEIRL (Cys53-Cys56)
4949.4455	4949.4499	-0.89	161	205	FKDVESDSAKQFLQAAEAIDDIPFGITSNSDVFSKYQLDKDGVVL
5081.3251	5081.3327	-1.49	444	489	KFFPASADRTVIDYNGERTLDGFKKFLESGGQDGAGDDDDLEDLE E
5134.5923	5134.5950	-0.54	30	76	RKSNFAEALAAHKYLLVEFYAPWCGHCKALAPEYAKAAGKLKAEG SE (Cys53-Cys56)
5291.5968	5291.5971	-0.06	105	154	FRNGDTASPKEYTAGREADDIVNWLKKRTGPAATTLPDGAAAESL VESSE
5516.8661	5516.8642	0.34	30	79	RKSNFAEALAAHKYLLVEFYAPWCGHCKALAPEYAKAAGKLKAEG SEIRL (Cys53-Cys56)
5779.8240	5779.8266	-0.45	393	443	YAPWCGHCKQLAPIWDKLGETYKDHENIVIAKMDSTANEVEAVKV HSFPTL (Cys397-Cys400)
5877.9364	5877.9450	-1.46	105	160	FRNGDTASPKEYTAGREADDIVNWLKKRTGPAATTLPDGAAAESL VESSEVAVIGF
6096.0597	6096.0666	-1.14	49	104	YAPWCGHCKALAPEYAKAAGKLKAEGSEIRLAKVDATEESDLAQQ YGVRGYPTIKF (Cys53-Cys56)
6154.9981	6155.0060	-1.28	390	443	VEFYAPWCGHCKQLAPIWDKLGETYKDHENIVIAKMDSTANEVEA VKVHSFPTL (Cys397-Cys400)
6171.2421	6171.2474	-0.86	206	258	FKKFDEGRNNFEGEVTKENLLDFIKHNQLPLVIEFTEQTAPKIFGGE IKTHIL
6243.1369	6243.1350	0.30	48	104	FYAPWCGHCKALAPEYAKAAGKLKAEGSEIRLAKVDATEESDLAQ QYGVRGYPTIKF (Cys53-Cys56)
6284.3259	6284.3314	-0.88	206	259	FKKFDEGRNNFEGEVTKENLLDFIKHNQLPLVIEFTEQTAPKIFGGE IKTHILL
6471.2369	6471.2461	-1.42	46	104	VEFYAPWCGHCKALAPEYAKAAGKLKAEGSEIRLAKVDATEESDL AQQYGVRGYPTIKF (Cys53-Cys56)
6612.2699	6612.2709	-0.15	17	76	MDAPEEEDHVLVLRKSNFAEALAAHKYLLVEFYAPWCGHCKALAP EYAKAAGKLKAEGSE (Cys53-Cys56)
6994.5395	6994.5401	-0.08	17	79	MDAPEEEDHVLVLRKSNFAEALAAHKYLLVEFYAPWCGHCKALAP EYAKAAGKLKAEGSEIRL (Cys53-Cys56)
7268.2596	7268.2488	1.49	444	508	KFFPASADRTVIDYNGERTLDGFKKFLESGGQDGAGDDDDLEDLE EAEEPDMEEDDDQKAVKDEL
7571.7831	7571.7761	0.92	105	175	FRNGDTASPKEYTAGREADDIVNWLKKRTGPAATTLPDGAAAESL VESSEVAVIGFFKDVESDSAKQFLQA
7651.9099	7651.9065	0.44	35	104	AEALAAHKYLLVEFYAPWCGHCKALAPEYAKAAGKLKAEGSEIRLA KVDATEESDLAQQYGVRGYPTIKF (Cys53-Cys56)
8284.2522	8284.2459	0.77	30	104	RKSNFAEALAAHKYLLVEFYAPWCGHCKALAPEYAKAAGKLKAEG SEIRLAKVDATEESDLAQQYGVRGYPTIKF (Cys53-Cys56)
9761.9239	9761.9218	0.21	17	104	MDAPEEEDHVLVLRKSNFAEALAAHKYLLVEFYAPWCGHCKALAP EYAKAAGKLKAEGSEIRLAKVDATEESDLAQQYGVRGYPTIKF (Cys53-Cys56)
